# Measures of Daily Activities Associated With Mental Health (Things You Do Questionnaire): Development of a Preliminary Psychometric Study and Replication Study

**DOI:** 10.2196/38837

**Published:** 2022-07-05

**Authors:** Nickolai Titov, Blake F Dear, Madelyne A Bisby, Olav Nielssen, Lauren G Staples, Rony Kayrouz, Shane Cross, Eyal Karin

**Affiliations:** 1 eCentreClinic School of Psychological Sciences Macquarie University Sydney Australia

**Keywords:** anxiety, depression, satisfaction with life, COVID-19, behavior, habits, cognitions, survey, mechanisms, psychological well-being

## Abstract

**Background:**

A large body of research has identified modifiable cognitions and behaviors (actions) associated with psychological health. However, little is known regarding the actions that are most strongly associated with psychological health or the frequency with which they should be performed.

**Objective:**

This paper described 2 studies that used survey methodology to create the Things You Do Questionnaire (TYDQ), which aims to identify and rank actions (items) and domains of actions (factors) most strongly associated with psychological health.

**Methods:**

We used digital marketing strategies to recruit Australian adult participants, who were asked to complete 2 web-based surveys comprising versions of the TYDQ; validated measures of depression, anxiety, and satisfaction with life; and demographic questions. In study 1, a total of 3040 participants rated how often they performed each of the 96 items comprising the TYDQ. This design was replicated in study 2, in which a 59-item version of the TYDQ was completed by 3160 participants. In both studies, the factor structure and validity were examined, as were the associations between individual TYDQ items and 3 mental health outcomes: depression, anxiety, and satisfaction with life.

**Results:**

In study 1, factor analyses revealed that a 5-factor model comprising 27 items achieved an optimum balance between brevity and variance and accounted for 38.1%, 31.4%, and 33.2% of the variance in scores on measures of depression, anxiety, and satisfaction with life, respectively. The factors were interpreted as realistic thinking, meaningful activities, goals and plans, healthy habits, and social connections. These 5 factors were more strongly associated with psychological health than those such as practicing kindness, exercising gratitude, and practicing spirituality. This pattern of results was replicated across gender, age groups, and depression severity. The 5-factor solution found in study 1 was replicated in study 2. Analyses revealed that a 21-item version accounted for 46.8%, 38.2%, and 38.1% of the variance in scores on measures of depression, anxiety, and satisfaction with life, respectively.

**Conclusions:**

These findings indicate that some actions are more strongly associated with psychological health than others and that these activities fall within 5 broad domains, which represent skills often taught in psychological treatments. Subsequent studies are planned to explore the reliability of these items and results in other samples and to examine patterns of change in scores during treatment for anxiety and depression. If replicated, these efforts will assist in the development of new psychological interventions and provide an evidence base for public mental health campaigns designed to promote good mental health and prevent the emergence of common mental disorders.

## Introduction

Depressive and anxiety disorders are highly prevalent conditions associated with significant distress, disability, and reduced quality of life [1,2]. These conditions exist along a continuum of severity, and it is now recognized that subclinical or subsyndromal variants can also cause functional disability [3] and considerable economic costs [4]. These disorders are characterized by maladaptive patterns of cognition, behavior, habits, and interpersonal and social engagement, which form the basis for accepted diagnostic criteria [5,6]. Maladaptive patterns of thinking and behavior trigger symptoms and impede recovery. Although the specific mechanisms of change during psychotherapy are not well understood [7-9], the premise that these maladaptive patterns can be modified or changed underlies the teaching of psychological skills within several treatment approaches, including cognitive therapy [10], behavior therapy [11,12], mindfulness [13], and interpersonal therapy [14].

Therefore, identifying adaptive and maladaptive patterns is a priority during treatment, and several approaches have been used to deconstruct these patterns into measurable and modifiable cognitions and behaviors (*actions*). One approach involves creating questionnaires to measure the quality and frequency of use of cognitive and behavioral skills (cognitive behavioral therapy) taught in psychological treatments [15-21]. Examples of items in these questionnaires include *I made an effort to evaluate my negative thoughts by considering just the facts* [19] and *I worked on a project that was meaningful to me* [21]. Consistent with psychological models, these and other studies have found small to moderate relationships between the frequency and quality of cognitive behavioral therapy–related actions and symptom severity over treatment. Although useful in clinical settings, disadvantages with many of these questionnaires include their tendency to focus solely on skills taught in therapy, their frequent use of highly technical language to describe cognitions and behaviors, and the exclusion of a large range of other activities that might be linked to mental health outcomes and that might represent important targets for intervention.

Other researchers surveyed consumers and mental health professionals to generate lists of actions believed to affect the symptoms of anxiety and depression [22-26]. Examples of actions identified in these studies include *focusing on the positive* [25] and *doing more things you enjoy* [22]. Many of the actions identified in this research are consistent with the skills taught and promoted in psychological treatments. Unsurprisingly, studies have also shown that prompting these types of actions is associated with symptom reduction [27]. However, a weakness of many of these measures is that they fail to inquire about the frequency of performing such actions. Such information can be important for developing interventions to improve mental health or prevent mental ill health.

An additional and parallel body of work has sought to examine the relationship between mental health and quality of life. This has led to the development of numerous measures, such as the Quality of Life Inventory [28] and the World Health Organization Quality of Life Scale [29], which have been demonstrated to reliably and validly measure satisfaction across multiple life domains (eg, physical health, psychological health, and relationships) while being reliable across both general and clinical populations. More recent quality of life measures, such as the Brunnsviken Brief Quality of Life Scale [30], have aimed to create freely accessible and brief versions that maintain strong validity and reliability profiles. Although helpful in determining satisfaction levels across different life domains, quality of life measures have not been designed to inquire about the frequency with which people engage in modifiable activities, which limits the utility of these tools.

Several research domains have identified actions that are associated with psychological health; however, several important questions remain unanswered. First, we do not know how often actions need to be performed each day or week for a person to experience an improvement in psychological health. Second, we do not know which actions or domains of actions are most strongly associated with improved mental health. Third, research has not yet explored whether particular actions are more helpful for different people according to their clinical presentation, personality, or demographic characteristics.

The studies presented in this paper are part of a research program, the Things You Do Project, designed to examine these questions with the overall aim of identifying and confirming the everyday actions that are most strongly associated with good mental health. The potential value of this information is not only in the treatment of mental disorder but also in the evidence-based prevention of mental disorder and promotion of mental health at the population level. Consistent with the World Health Organization’s definition of health as not only the absence of symptoms but also the presence of a positive state of well-being [31], we used the term *psychological health* in this paper to describe not only the absence or reduction in symptoms of anxiety or depression but also an improvement in well-being, measured based on self-reported satisfaction with life.

In this paper, we have described 2 studies that used survey methodology to create the Things You Do Questionnaire (TYDQ), which aims to identify and rank actions and domains of actions most strongly associated with psychological health. Recognizing the complexity of this objective and the range of potential methods, we opted for a broad and systematic approach to scale development [32].

In the first study, participants rated the frequency with which they completed a range of actions previously identified to be associated with psychological health. Responses were analyzed to assess the factor structure, reliability, validity, and strength of association of TYDQ items with psychological health. In the second study, participants rated a shorter version of the TYDQ. In both studies, participants also completed validated measures of depression, anxiety, and satisfaction with life. Formal hypotheses were not proposed, but we expected that the process of ranking actions and domains of actions would generate a parsimonious list of psychological health items.

## Study 1

The aims of study 1 were to (1) generate a large list of actions previously associated with psychological health and explore the relationship between the weekly frequency of performing these actions and symptoms of anxiety, depression, and life satisfaction; (2) explore the underlying factor structures of the TYDQ and rank the association between the actions and factors and psychological health; (3) explore the importance of different actions across different subgroups; and (4) develop a parsimonious list of items.

### Methods

#### Ethics Approval

A web-based survey design was used for data collection. The study was reviewed and approved by the Macquarie University Human Research Ethics Committee (MQ HREC: 5201700988), and informed consent was obtained from all participants. All methods were performed in accordance with the relevant guidelines and regulations.

The data sets used and analyzed during this study are available from the corresponding author (NT) upon reasonable request.

#### Questionnaire and Item Generation

An initial list of items was derived from a review of the literature on actions associated with psychological skills identified through multiple sources. These included reviews of (1) skills typically taught in psychological therapies (eg, cognitive skills, behavioral activation, exposure, goal setting, problem solving, social skills training, applied relaxation, mindfulness, acceptance, gratitude, and kindness); (2) actions associated with psychological health, such as healthy daily routines (eg, sleep hygiene, nutrition, and moderated use of electronic devices), physical health (eg, exercise, yoga, stretching, and walking), social activity (eg, talking to friends and spending time with people), meaningful and satisfying activities (eg, doing fun activities), and spiritual and religious activities; (3) symptoms of high prevalence mental disorders as described in psychiatric diagnostic systems [5,6]; (4) previous questionnaires developed by the authors [20], other questionnaires, or similar lists [17-19,22,23]; and (5) consultations with colleagues.

Our research team, consisting of psychiatrists, clinical psychologists, and data analysts with experience in questionnaire development, created a preliminary list comprising >500 actions. Each action was phrased as a verb, and references to psychological techniques or orientations, such as practicing mindfulness, were avoided. During the planning phase, we met regularly to identify, review, edit, and categorize the list, which was reduced to 272 items after duplicates and overlapping items were removed. The items were categorized according to the mechanisms or processes associated with each item; that is, a priori attempts were made to identify theoretical clusters. For example, some items were categorized as primarily concerned with cognitive actions (primary cluster), and within this cluster, they were subdivided into actions associated with challenging unhelpful thoughts, focusing on the future (secondary clusters). In instances where items belonged to multiple clusters, categorization was based on consensus. To capture a broad range of items, the list was shared with colleagues outside the research group, who made further recommendations. The final list of 96 items was based on the following criteria: (1) actions that can be performed daily; (2) measurable, that is, a person can be expected to reliably estimate the frequency of actions over a week; (3) can be completed by most adults; (4) described as a positive action, that is, an action that can be considered a strength; (5) modifiable, that is, can be changed by the person; and (6) did not duplicate another item.

A 5-point Likert rating scale was used to ask participants how often they performed these behaviors over the past week by using the following scoring system: *0=Not at all (0 days per week)*; *1=1 or 2 days per week*; *2=3 to 4 days per week*; *3=5 to 6 days per week*; *and 4=every day (7 days per week)*. None of the items were reverse scored. After completing the TYDQ, participants were also invited to nominate other actions that were not included in the 96 items they used to improve their mental health.

#### Participants and Procedure

The survey was promoted to adults across Australia via websites and web-based newsletters from Australian mental health services and Facebook posts and advertisements. Advertisements invited people to participate in a study that aimed to *identify activities and habits that affect mental health*. Consenting participants provided demographic details and completed web-based questionnaires. Participants were required to be aged ≥18 years. No other exclusion criteria were applied. Details of the sample are included in the *Results* section.

#### Measures

Three standardized outcome measures were used to evaluate and rank the association of the items with psychological well-being.

#### Patient Health Questionnaire 9-Item Scale

The Patient Health Questionnaire 9-item (PHQ-9) scale measures the occurrence of Diagnostic and Statistical Manual IV–congruent depressive symptoms over the past 2 weeks by using a 4-point Likert scale ranging from 0 (*not at all*) to 3 (*nearly every day*). Higher scores indicate greater symptom severity [33]. The PHQ-9 has good internal consistency and is sensitive to change [34], with a total score of ≥10 usually but not always associated with a diagnosis of major depressive disorder [35,36]. The Cronbach α in this study was .90.

#### Generalized Anxiety Disorder 7-Item Scale

The Generalized Anxiety Disorder 7-item (GAD-7) scale measures the occurrence of general anxiety symptoms over the past 2 weeks by using a 4-point Likert scale ranging from 0 (*not at all*) to 3 (*nearly every day*). The GAD-7 is sensitive to Diagnostic and Statistical Manual IV–congruent GAD, social phobia, and panic disorder, with increasing scores indicating greater severity of symptoms [34,37]. The GAD-7 has been shown to demonstrate sound psychometric properties, with a total score ≥10 usually indicating a likely diagnosis of an anxiety disorder [38]. The Cronbach α in this study was .92.

#### Satisfaction With Life Scale

The Satisfaction With Life Scale (SWLS) is a 5-item scale that measures attitudes toward life satisfaction by using a 7-point Likert scale ranging from 1 (*strongly disagree*) to 7 (*strongly agree*), with higher scores indicating greater life satisfaction. The SWLS has demonstrated good psychometric properties [39,40] and has been used extensively as a measure of life satisfaction in mental health research [41]. The Cronbach α of the SWLS in this study was .90.

#### Statistical Analyses

The examination of the relationship between the TYDQ items and the standardized scales was operationalized in three detailed and comprehensive steps: (1) item-level analysis, (2) factor analysis, and (3) generalizability of factor solutions.

#### Item-Level Analysis

First, a series of univariate regression models (ANOVA) was used to quantify and statistically test the association between each of the TYDQ item scores and the PHQ-9, GAD-7, and SWLS outcome scores. The strength of the association between items and outcomes was summarized with an *R*^2^ metric, noting the proportion of the variance explained by each TYDQ item and each outcome. The *R*^2^ values were used to weigh and rank the 96 items.

To further visualize the relative importance of different TYDQ items, each possible TYDQ item score, ranging from 0 (*not at all*) to 4 (daily), was tabulated with its corresponding PHQ-9, GAD-7, and SWLS outcomes to form a heat map [42]. Comparing the corresponding outcome scores with the range of TYDQ values enabled the identification of the minimal weekly behavioral frequency score needed to achieve optimal PHQ-9, GAD-7, and SWLS outcome scores for each TYDQ item. The optimal TYDQ score was defined as the minimum weekly behavioral frequency beyond which no additional statistically significant improvement can be observed.

#### Factor Formation and Analysis

Second, a series of exploratory factor analyses (EFAs) was used to examine how different TYDQ items formed composite latent factors of behavior. An initial EFA was used to identify the factors based on a full list of 96 items. An additional 3 EFAs were conducted to identify latent factors among the subsets of TYDQ items that were associated more closely with the PHQ-9, GAD-7, and SWLS. These EFAs analyzed the subsets of items based on a minimum of 5%, 10%, and 15% *R*^2^ relationship to any one mental health outcome with the aim of identifying more parsimonious lists of factors closely related to psychological health. A factor was considered viable when the item set comprised a composite eigenvalue greater than 1. Additional diagnostic analyses were used to assess each of the identified factors, including item reliability analysis, mean item intercorrelation, and the ability of the identified factors to replicate each item (ie, commonalities).

All identified factors were tested and ranked for their strength of association with the 3 outcomes by using the same analytics and visualization approach used to evaluate TYDQ items. To further evaluate the importance of different behavioral TYDQ factors for mental health, a series of binary logistic regressions was conducted to test the association between the factor scores and the probability of an individual presenting with PHQ-9 or GAD-7 scores that would be considered within the clinical range.

Each factor was assigned a proposed label, which aimed to represent the overall theme of those items. Consensus regarding the factor label was achieved through discussion and debate, considering other labels could have been used.

#### Generalizability of Factors Solutions Across Different Subgroups

A series of confirmatory factor analyses (CFAs) were conducted to examine the reliability of the EFAs within a sample cross-validation analysis (5 randomized subsamples) and the validity of the factor structure along with key subgroups that differed based on age (<30, 30-44, 45-60, >60 years), gender (male, female, and other), the severity of depressive or anxiety symptoms (minimal-mild, moderate, and severe), an indication of employment, and tertiary education. These CFA tests followed the methodology and reporting conventions outlined by Putnick and Bornstein [43] for the examination of measurement invariance, including the examination of configural invariance, metric invariance, scalar invariance, and strict invariance. From the viewpoint of scale development, this step sought to evaluate the reliability and generalizability of the identified factor solutions along relevant subgroups in clinical research (dimensionality).

All analyses were performed using R statistical software version 4.1.1 [44] and the Lavaan package [45]. A conservative *P* value of .01 was considered the threshold for statistical significance, reflecting the large sample and the multiple number of tests conducted. In all analyses, participants with missing data were not included in the analyses owing to difficulty imputing unbiased missing outcomes in a high-dimensional analysis and cross-sectional settings. All EFAs and CFAs were based on weighted least-squares estimators to account for the ordinal categorical TYDQ item score range. For all EFAs, with the aim of identifying unique subsets of items, a varimax factor rotation was adopted to maximize the number of factors identified and minimize item-factor cross-loadings [46].

Items with factor loadings <0.50, a threshold that aimed to achieve a conservative balance between recommendations in the literature [32,47], were suppressed as were items with suboptimal association with their corresponding factor (accounting for <25% of the factor variance) [48].

### Results

#### Item and Sample Description

Participants in study 1 were recruited from March to June 2018, during which time 3755 people consented to participate and 3040 (80.96%) completed the questionnaire. Sample characteristics are presented in Table 1. Participants had a broad range of both demographics (age, gender, location, education, and employment) and mental health symptoms. Approximately half of the sample scored above the clinical cutoffs on the PHQ-9 (n=1710, 48.1%), GAD-7 (n=2020, 57.9%), and SWLS (n=2188, 64%), allowing the testing of the association of everyday behaviors for those above and below recognized clinical cutoffs on the PHQ-9 and GAD-7.

Multimedia Appendix 1 includes each of the 96 items ordered based on the a priori primary and secondary theoretical clusters created during item development and based on the observed average weekly score. Multimedia Appendix 1 also uses a heatmap to indicate the frequency with which participants reported performing each item each week. For example, the first item in Multimedia Appendix 1 was the 13th item presented in the survey and read I read, listened, or watched something I enjoyed. A priori, this item was categorized as an Activity/Meaning (primary and secondary clusters), and the average weekly score was 2.72 (mean TYDQ weekly score), indicating that the mean frequency reported was between 4 and 6 days each week. The final column indicates that 37.4% (n=1404) of the sample reported doing this action daily, whereas 3.7% (n=139) reported not doing this action in the previous 7 days.

As shown in Multimedia Appendix 1, the frequency of actions (TYDQ mean scores) ranged across and within clusters. For example, 2 of the highest frequency scores were observed within the *Healthy Routine* cluster, with daily showers or baths reported by 69.3% (n=2602) of the sample, and avoidance of illicit drugs or misuse of medication reported by 83.3% (n=3128).

**Table 1:** Sample characteristics of studies 1 and 2.

Variable and subgroup			Study 1 (year 2018; N=3755)		Study 2 (year 2020; N=3756)		Test of sample differences
**Survey completion, n (%)**							χ^2^_1,7510_=13.1, *P*<.001
	Completed survey	3040 (81)		3160 (84.1)			
	Incomplete survey	715 (19)		596 (15.9)			
**Sex, n (%)**							χ^2^_2,7509_=303.8, *P*<.001
	Male	1806 (48.1)		1072 (28.5)			
	Female	1924 (51.2)		2651 (70.6)			
	Other	25 (0.7)		33 (0.9)			
**Age (years)**							*t*_7509_=16.13, *P*<.001
	Value, mean (SD)	41.9 (13.9)		47.7 (17.1)			
	<30, n (%)	976 (26)		944 (25.1)			
	30-45, n (%)	1416 (37.7)		1430 (38.1)			
	45-60, n (%)	1137 (30.3)		925 (24.6)			
	>60, n (%)	226 (6)		456 (12.1)			
**PHQ-9^a^ category, n (%)**							χ^2^_1,7095_=159.1, *P*<.001
	Below cutoff (≥10)	1710 (48.1)		2227 (62.9)			
	Above cutoff (<10)	1848 (51.9)		1311 (37.1)			
**GAD-7^b^ category, n (%)**							χ^2^_1,6961_=151.3, *P*<.001
	Below cutoff (≥10)	1471 (42.1)		2497 (71.9)			
	Above cutoff (<10)	2020 (57.9)		974 (28.1)			
**SWLS^c^ category, n (%)**							χ^2^_1,6858_=98.4, *P*<.001
	Below cutoff (≥16)	2188 (64)		2578 (75)			
	Above cutoff (<16)	1233 (36)		860 (25)			
Depression symptoms (PHQ-9), mean (SD)			10.7 (6.8)		8.5 (6.5)		*t*_7095_=14.3, *P*<.001
Anxiety symptoms (GAD-7), mean (SD)			8.8 (5.8)		6.8 (5.6)		*t*_6961_=14.17, *P*<.001
Satisfaction with life (SWLS), mean (SD)			18.4 (7.7)		20.8 (7.8)		*t*_6858_=13.42, *P*<.001
**Australian born, n (%)**							χ^2^_1,6049_=32.9, *P*<.001
	No	632 (16.8)		829 (22.1)			
	Yes	3123 (83.2)		2927 (77.9)			
**Location, n (%)**							χ^2^_2,7392_=4.9, *P*=.09
	Capital city	1921 (51.2)		2015 (54.5)			
	Other urban region	934 (24.9)		898 (24.3)			
	Rural or remote	841 (22.4)		785 (21.2)			
**Education attained, n (%)**							χ^2^_3,7391_=75.9, *P*<.001
	High school or less	744 (19.8)		589 (15.9)			
	Other tertiary qualification or certificate	1200 (32)		1100 (29.7)			
	Postgraduate degree	735 (19.6)		1040 (28.1)			
	Undergraduate degree	1017 (27.1)		969 (26.2)			
**Employment status, n (%)**							χ^2^_5,7389_=245.9, *P*<.001
	Paid employment	2335 (62.2)		2031 (54.9)			
	Student	383 (10.2)		285 (7.7)			
	Home duties or parenting	177 (4.7)		154 (4.2)			
	Disability support	196 (5.2)		188 (5.1)			
	Unemployed or seeking employment	318 (8.5)		289 (7.8)			
	Retired	287 (7.6)		751 (20.3)			
**Marital status, n (%)**							χ^2^_3,7391_=119.4, *P*<.001
	Single or ever married	996 (26.5)		1125 (30.4)			
	Widowed	55 (1.5)		145 (3.9)			
	Separated or divorced	378 (10.1)		555 (15)			
	Married or defacto	2267 (60.4)		1873 (50.6)			
**Seeing mental health professional about anxiety or depression? n (%)**							χ^2^_3,7394_=60.0, *P*<.001
	Never	747 (19.9)		999 (27)			
	Previously	1737 (46.3)		1714 (46.3)			
	Currently	1212 (32.3)		985 (26.6)			
**Taking medication for anxiety or depression? n (%)**							χ^2^_1,7451_=18.5, *P*<.001
	No	2356 (62.7)		2496 (67.5)			
	Yes	1399 (37.3)		1202 (32.5)			

^a^PHQ-9: Patient Health Questionnaire 9-item.

^b^GAD-7: Generalized Anxiety Disorder 7 item.

^c^SWLS: Satisfaction With Life Scale.

#### Item-Level Analysis

The 96 items were then subjected to tests of the strength of association with each of the PHQ-9, GAD-7, and SWLS outcomes. The results from this first series of analyses are presented in Multimedia Appendix 2 for each item, with items grouped around the a priori clusters. The columns of the table report the mean PHQ-9, GAD-7, and SWLS scores observed with differing rates of weekly behavioral frequency. The PHQ-9, GAD-7, and SWLS scores were further incorporated into a heat map visualization to highlight the TYDQ items associated with the highest symptom scores among the 96 TYDQ items, where red hues indicate deterioration of psychological health (ie, symptom scores and satisfaction with life) and blue hues indicate improved psychological health.

The proportion of symptom variance explained (*R*^2^) was reported as a summary measure of the association between each item and outcome. In Multimedia Appendix 2, the presentation order of the 96 items illustrates the relative ability of TYDQ items to account for the cumulative variance of the 3 outcomes, with the strongest items reported at the top of each a priori cluster. Together, the 96 items were significantly associated with each of the 3 outcomes (TYD outcome regression β test resulting in *P*<.001), except item TYDQ 57 (*I fulfilled my responsibilities even though I didn’t want to*) and its association with GAD-7 outcomes. Most items showed a linear relationship between higher frequency and lower symptom scores, with a minority of items showing a flattened, convexed, or positive relationship with PHQ-9 and GAD-7 outcomes. However, the strength of the association between each of the items and each outcome varied substantially, both within and across clusters. For example, TYDQ 16, *I treated myself with respect*, illustrated the strongest association with depressive symptoms (accounting for 22.5% of all PHQ-9 scores); TYDQ 70, *I kept a realistic perspective on things*, was the strongest correlate of anxiety symptoms (accounting for 21.1% of all GAD-7 scores); and TYDQ 54, *I had something to look forward to*, was the strongest correlate of satisfaction with life (accounting for 25.9% of all SWLS scores). Across the items, the threshold of activity associated with optimal well-being varied from half a week to daily, with the majority of items requiring frequent (*almost every day*) but not necessarily daily activity.

#### Factor Formation and Analysis

EFAs were conducted to identify underlying groupings among the items and to explore a brief, latent factor structure of behaviors associated with psychological health. The results from the EFA of the complete 96-item list are presented in Multimedia Appendix 3. In total, 16 factors were identified, with 56 of the items demonstrating factor loadings above 0.5 for any 1 factor. Factors with loadings of ≤0.5 were suppressed. Across the TYDQ list, the factors appeared to form along their a priori clusters, with most items grouped within their clusters. No item-factor cross-loadings were identified above the loading of 0.5.

The results of the tests of association between the resulting factor scores and each of the PHQ-9, GAD-7, and SWLS outcomes are further reported in Multimedia Appendix 3, which describes the outcomes of a series of 4 EFAs, each testing a different number of items (see Multimedia Appendix 3 for items). This shows that the complete list of 16 factors, based on 56 items, accounted for 41%, 37%, and 32% of the outcome *R*^2^, respectively. The resulting 16 factors can also be seen as varying in their strength of association with the 3 outcomes, with estimates of *R*^2^ ranging from <0.1% to 24%.

The EFA was repeated with another subset of items (68 items), selected based on the individual item’s ability to account for at least 5% of any of the 3 symptom outcomes (*R*^2^>5%), which provided an opportunity to examine patterns among TYDQ items more closely related to psychological health. The analysis identified 11 factors, with only 45 items demonstrating factor loadings >0.5 for any one factor. The items were clustered within their respective a priori clusters, and no item-factor cross-loadings were identified. A test of the association between the resulting factor scores and each of the PHQ-9, GAD-7, and SWLS outcomes illustrated that a complete list of 11 factors based on 45 items accounted for 33%, 29%, and 32% of the outcome *R*^2^, respectively, with each of the individual factors varying in their strength of association from 5% to 21% (Multimedia Appendix 4).

In a third EFA, an even briefer subset of items (35 items) was selected based on the item’s ability to account for a minimum of 10% of any of the 3 symptom outcomes. This resulted in 5 factors and 27 items with a factor loading above 0.5 for any 1 factor. No item-factor cross-loadings were identified. A test of association between the resulting factor scores and each of the PHQ-9, GAD-7, and SWLS outcomes revealed a list of 5 factors based on 27 items, which accounted for 38%, 31%, and 33% of the outcomes *R*^2^, respectively (Multimedia Appendix 4). The formation of factors largely followed the a priori clusters, and the resulting factors varied 3-fold in their strength of association with each of the outcomes (9%-28%).

A fourth EFA, which included items, identified a stringent *R*^2^>15% criterion. This resulted in a brief set of items (8 items) found to represent a single composite factor combining key items from several domains. Although this result is interesting, the PHQ-9, GAD-7, and SWLS variance explained by a single factor (Multimedia Appendix 4) was suboptimal (*R*^2^=36.6%, *R*^2^=26.7%, and *R*^2^=32.9%, respectively), and the representation of multiple domains, including activity planning, self-representation, and values through a single nonspecific factor, would inevitably limit the ability to assess and interpret the type of behavior.

Together, these EFAs illustrate that the 96-item list could be reduced to a more parsimonious and selective item list, with an optimal 5-factor, 27-item solution that retained >90% of the strength of association with each of the symptom outcomes, with only a quarter of the items (27/96, 28%). Each factor solution was further assessed for item reliability, mean item intercorrelation within each factor, and the ability of the identified factors to replicate each item (commonalities), as collated in Multimedia Appendix 5.

#### Generalizability of Factor Solutions Across Different Subgroups

A series of CFAs were conducted to examine the measurement invariance of the 5-factor solution as a brief but sensitive measure of psychological well-being across 5 categories, namely age groups, gender, depression and anxiety symptoms, education, and employment. The results from the CFAs are collated in Multimedia Appendix 6. The identification of TYDQ differences in the intercept and residual scores indicated that gender, age, and baseline symptoms were associated with different frequencies of weekly activity, although the TYDQ scores formed the same latent patterns across these groups. To check for the potential of alternate models, we reran the EFA within each of the age, sex, and PHQ-9 subgroups. The results identified the 5-factor solution reliably replicated as the most prominent latent solution when no parameter restraints were made or when the rotation methodology was changed (Multimedia Appendix 7). Together, the results from these CFAs illustrate that the latent 5-factor structure identified was statistically reliable, generalized across subgroups of clinical interest, and replicated across differing aspects of the statistical methodology (rotation and parameter constraint).

### Discussion

Study 1 involved a comprehensive series of methodological and statistical steps designed to create a large list of actions associated with psychological health, test their strength of association with defined mental health outcomes, and develop a parsimonious list of items. To begin with, 96 items previously associated with psychological health were identified and administered to a large and diverse adult sample, along with measures of depression, anxiety, and satisfaction with life.

Several factor solutions were explored, and through a systematic process of dimension reduction, EFA solutions based on 56 items, 45 items, 27 items, and 8 items were examined. The 27-item solution, which comprised 5 factors, was found to be optimal, as it represented 38%, 31%, and 33% of the variance in scores of measures of depression, anxiety, and satisfaction with life, respectively. A series of CFAs indicated that the factor structure was robust across different subgroups, with these 5 factors interpreted as (1) realistic thinking, (2) meaningful activities, (3) goals and plans, (4) healthy habits, and (5) social connections.

Surprisingly, some items and factors that were expected to show strong relationships with psychological health, such as actions associated with kindness, gratitude, and spirituality, were not represented in the 5-factor solution. In contrast, items associated with skills commonly taught in psychological treatments, such as challenging unhelpful thoughts and engaging in pleasant activities, were very strongly associated with psychological health. Importantly, the results of study 1 not only identified items and factors that are strongly associated with psychological health but also found that the frequency of performing those actions was associated with improved well-being.

In summary, the results of study 1 provided preliminary psychometric evidence for the TYDQ as a measure of modifiable actions associated with psychological health. The reliability of the strong relationship between the 5-factor 27-item list found in study 1 needs to be replicated in further studies, which is one of the aims of study 2. Given the surprising finding that some items and factors were not strongly associated with psychological health, such items should also be retested.

## Study 2

### Overview

The objective of study 2 was to explore the reliability of the results of study 1 by replicating the design used in study 1 using a briefer version of the TYDQ. The aims were to (1) generate a shortened list of items based on the results of study 1 and explore the relationship between the weekly frequency of performing these items and outcomes of psychological health; (2) explore the underlying factor structures and rank the association between the items, factors, and psychological health; (3) explore the importance of different actions across different subgroups; and (4) develop a parsimonious list of items.

It should be noted that study 2 was conducted when major cities across Australia were locked down in response to the COVID-19 pandemic, which, on the one hand, represents a significantly changed context to that of study 1 but, on the other hand, provides an opportunity to test the items and the questionnaire in a psychologically challenging context.

### Methods

#### Ethics Approval

The design of study 2 replicated that used in study 1. Ethics approval for data collection was obtained from the Macquarie University Human Research Ethics Committee (MQ HREC: 5201700988), and informed consent was obtained from all participants.

#### Questionnaire

Study 2 evaluated a version of the TYDQ comprising 59 of the 96 original items (Multimedia Appendix 8), with each item using the same 5-point rating scale used in study 1. This brief list was constructed in several steps. First, items that accounted for at least 5% of the PHQ-9, GAD-7, and SWLS were identified, resulting in an initial list of 67 items. Second, this list was edited to remove items with similar wording or duplicated items, resulting in the removal of 23 items. Third, 15 additional items from the original 96 item list were added to test the association between items, such as kindness to others and gratitude, which were considered clinically important and expected in study 1 to be associated with the outcomes but were not. We reviewed each of these steps.

#### Participants and Procedure

The procedure used in study 1 was repeated in study 2. As shown in Table 1, a total of 3756 participants consented to participate in the study and started filling the questionnaires, which were available between June and August 2020. These dates coincided with social and travel restrictions across Australia owing to the impact of the COVID-19 pandemic.

#### Other Measures

The outcome measures used in study 1 were also used in study 2.

#### Statistical Analyses

The three analytical steps in study 1 were repeated: (1) item-level analyses, (2) factor formation and analyses, and (3) analyses that explored the generalizability of factor solutions across different subgroups. Additional analyses were conducted to compare study 1 and study 2 samples on TYDQ scores, the strength of association between the items and symptom outcomes, and factors. For example, the comparison of TYDQ scores from the 2 studies was operationalized by treating the studies as a group variable and regressing this variable on the TYDQ item scores. Similarly, the association between TYDQ items and symptom outcomes was tested by regressing the TYDQ item score, group, and item-by-group interaction on outcome measures.

### Results

#### Item-Level Analysis

A total of 3756 people consented to participate in study 2, and 3160 (84.13%) completed all the questionnaires. Participant characteristics and comparison with the study 1 sample are presented in Multimedia Appendix 8. These results indicate significant differences between the samples across most demographic variables, symptom severity, satisfaction with life, use of medications for anxiety or depression, and mental health service use.

The TYDQ score for each item, the association of each TYDQ item with the 3 symptom outcomes, and testing of differences between the 2 study samples are included in Multimedia Appendix 8. The results indicated a significant increase in the average score of most TYDQ items from study 1 to study 2. The results also indicate that the strength of association between most TYDQ items and each of the 3 outcomes was greater in study 2 than in study 1. However, the relative strength of association across and between the items and the outcomes was similar in magnitude across the samples, as seen in the heat map.

#### Factor Formation, Item Reduction, and Analysis

In the second step, the 59 items were subjected to a dimension-reduction analysis. The complete list of 59 items identified a 10-factor solution when no parameter constraints were imposed on the data, with 9 of these factors overlapping with the study 1 EFA solution (Multimedia Appendix 9). Using the inclusion criterion of *R*^2^>10%, a 26-item solution was identified using the data from study 2, replicating the pattern of the 5 factors identified in study 1. Using the inclusion criteria of *R*^2^>10% and reanalyzing the results of study 1 resulted in an even more parsimonious 21-item solution (Multimedia Appendix 9) that reliably retained the factor structure. The 5 factors and the 21-item solution identified in the reanalysis of study 1 results (Multimedia Appendix 9; inclusion criteria of *R*^2^>10%) were then compared with the same 21 items from the study 2 results, and associations between the 5 factors and the 3 outcome variables were compared across samples (Multimedia Appendix 10). This demonstrated close similarities in strength, directionality, significance, and optimal frequency cutoffs between the 2 samples.

A set of 3 EFAs were then conducted for item subsets with no selection criteria, inclusion criteria of *R*^2^>10%, and inclusion criteria of *R*^2^>15%. The 5-factor 21-item solution associated with the *R*^2^>10% criteria in study 2 accounted for the highest amount of PHQ-9, GAD-7, and SWLS outcome variance (*R*^2^=46.8%, *R*^2^=38.2%, and *R*^2^=38.1%, respectively). Consistent with this, a reanalysis of the data from study 1 using the same *R*^2^>10% criteria demonstrated that this 5-factor 21-item solution accounted for the highest amount of PHQ-9, GAD-7, and SWLS outcome variance (*R*^2^=37.1%, *R*^2^=30.0%, and *R*^2^=30.5%, respectively).

#### Generalizability of Factor Solutions Across Different Subgroups

CFAs seeking to verify a 5-factor 21-item solution across the study 1 and study 2 samples resulted in metric invariance (similarities in factors and item loading) but not scalar or strict invariance (Multimedia Appendix 11). This result is consistent with the interpretation that participants from the 2 studies differed in their means but not item or factor importance.

In brief, the overall results of study 2 replicated the results and 5-factor model obtained in study 1, albeit with a 21- rather than a 27-item solution. These results confirm the strength of the relationship between key items in the TYDQ and psychological health outcomes and the underlying factor structure.

### Discussion

Study 2 examined the performance of a shortened version of the TYDQ. The survey was completed by 3160 people, and although the sample was significantly different from that of study 1, the overall patterns of results obtained in study 1 were replicated. The 5-factor structure observed in study 1 was supported and accounted for 46.8%, 38.2%, and 38.1% of the variance in the symptoms of depression, anxiety, and satisfaction with life, respectively. These solutions were achieved using a parsimonious 21-item list. Importantly, and again consistent with the results of study 1, the observations that some items and factors expected to show strong relationships with psychological health, such as showing kindness to others and expressing gratitude, were not found to be associated with well-being. The implications and limitations of these results are discussed in the subsequent section.

## General Discussion

This paper describes 2 studies reporting on the initial development and evaluation of the TYDQ, which aims to measure modifiable actions that are strongly associated with psychological health. The detailed and systematic approach followed accepted scale development methods [32] and attempted to address the limitations of previous work by including a broad range of modifiable actions, exploring their frequency over a 1-week timeframe, identifying the actions most strongly related to the target outcomes, and testing the generalizability of results across age, gender, and baseline symptom severity.

### Principal Findings

Study 1 examined the performance of a 96-item version of TYDQ. A 27-item 5-factor solution achieved an optimal balance between accounting for sufficient variance in outcome measures of depression, anxiety, and satisfaction with life and parsimony. These five factors were interpreted as actions concerned with (1) realistic thinking, (2) meaningful activities, (3) goals and plans, (4) healthy habits, and (5) social connections. There was a strong relationship between the frequency of performing the actions associated with these factors each week and improved psychological health, with results indicating that they should be performed at least half a week or more to optimize their psychological health. Importantly, the factor structure was consistent across demographic variables, including gender and age groups.

Overall, these findings were replicated in study 2, which evaluated a shortened 59-item version by using the same systematic analytic strategy. Study 2 was conducted during a period when participants were mostly in lockdown owing to the impact of the COVID-19 pandemic in Australia. However, the strength of the relationship between the frequency of target actions and outcomes increased relative to study 1. The same 5-factor solution found in study 1 was also found in study 2, although slight differences in the item loadings were observed across the 2 samples. An item-reduction exercise revealed that a 5-factor 21-item version solution achieved an optimal balance between item parsimony and accounting for sufficient variance across the 3 outcome measures. These results appear to confirm the robustness of the 5 factors but also suggest that the 21-item list (Multimedia Appendix 9) should not be considered final, and additional survey design efforts could further improve the brevity and validity of the items identified. In any case, the 96-item and 59-item lists are provided in Multimedia Appendix 11.

### Comparison With Prior Work

The overall results are consistent with previous research regarding key items associated with psychological health, including cognitions and actions such as, *I did something enjoyable, I kept a realistic perspective on things, I had something to look forward to, and I treated myself with respect*. These 5 factors confirm the importance of many of the skills taught in psychological interventions, including cognitive therapy [10], behavior therapy [12,49], and others. Various combinations of these skills and actions have been recognized as contributing to psychological health [50-52] and are frequently included in public mental health campaigns. Thus, the contribution of our preliminary study is not to identify the actions and factors associated with psychological health. Instead, these studies extend the literature by comparing the relative benefits of different types of actions on psychological health and the frequency with which these factors are associated with psychological health. The findings indicate that performing these actions for at least half the days of the week is important for psychological health across age, gender and demographic groups, as well as in different severity levels of mood symptoms. This proposed frequency needs to be carefully evaluated using a longitudinal study; however, if supported, it would indicate a key target for psychological interventions as well as for public health interventions designed to promote psychological health and prevent common mood disorders in the community.

An additional and important finding was that some actions were less strongly associated with psychological health than expected. For example, acts of kindness, practicing gratitude, and spirituality are widely considered important for psychological health; however, in this study, these actions were not found to be as important as those relevant to the 5 factors. This lack of findings is broadly consistent with the results of empirical studies [53-59].

For example, in a systematic review and meta-analysis of 27 experimental studies examining the relationships between acts of kindness and different measures of subjective well-being, including measures of psychological health, Curry et al [53] found the overall effect of kindness to be small to medium (δ=0.28); however, they noted that most of the reviewed studies were underpowered to detect small differences. In a series of meta-analyses examining the findings of 38 studies, Dickens [54] showed that when compared with waitlist, inactive, or no-treatment control groups, gratitude interventions were associated with decreased depressive symptoms and improved positive affect, well-being, happiness, and life satisfaction, albeit with small effect sizes (Cohen *d*=0.13−0.30). In a more recent review of the effect of gratitude traits and interventions, Jans-Beken et al [60] concluded that gratitude interventions were not found to reliably improve symptoms, such as depression and anxiety, but they were associated with improved happiness and life satisfaction. In a meta-analysis of 48 longitudinal studies, Garssen et al [56] reported an overall positive effect of religion or spirituality on mental health; however, similar to studies examining the relationships between mental health and acts of kindness and practicing gratitude, the effects were small, with a random weighted average effect size of *r*=0.08.

Given the large number and broad cross-section of people completing the 2 surveys, these findings appear to be robust. However, given the preliminary nature of the 2 studies reported here, we propose that the findings should not be interpreted as such actions are not helpful; rather, it is likely that different actions are important at different stages of psychological health and for particular individuals and age groups. For example, it is commonly recognized in clinical practice that people who are severely depressed are more likely to benefit from actions involving increasing reinforcement and pleasure than from practicing gratitude, but once their mood has lifted, actions such as practicing gratitude and showing kindness to others may help maintain psychological health. In addition, it should be noted that the results obtained here are relevant to the outcomes of interest, namely, symptoms of depression, anxiety, and satisfaction with life, and that choosing other outcome measures may result in other factors and items ascending in importance. This raises the possibility of future research exploring the relationship between everyday actions and a much broader range of mental health outcomes, including the general p factor, which extends beyond internalizing disorders to include externalizing and thought or psychotic disorders [61].

### Limitations

The preliminary and cross-sectional nature of the 2 studies reported here are associated with several limitations. First, despite the large sample sizes and consistent results across subsamples, the work should be considered preliminary and tested in other samples, including people with different cultural and demographic backgrounds. In addition, the items and factors identified here need to be tested using longitudinal or interventional research designs to gauge their sensitivity to change and reliability over time. The robustness of the 5 factors should also be tested across differing statistical methods for identifying dimensionality, for example, using different statistical criteria for selecting items or using latent profiling.

We also acknowledge that the initial selection of items used to develop the TYDQ, despite the breadth of our inquiries, involved some subjective choices. We have shared lists to assist independent replication and further development (Multimedia Appendix 12). Related to these limitations, we also acknowledge that the terms we generated to describe the factors were based on our clinical and psychometric decisions, and that other research groups may have generated other labels for each of the factors. We also acknowledge that although most of the actions were relatively simple to understand by participants, some actions were more difficult for participants to measure; for example, the item *I treated myself with respect*. This raises important questions about how people interpreted some of the items, which may in turn affect both the reliability and validity of the results. This is further complicated by the observation that a person’s interpretation of the questions may change over time and with their experience of the action. Finally, we acknowledge that the focus of this study is primarily on the relationship between actions and symptoms and may not be generalizable to other concepts of psychological well-being, happiness, or thriving [62].

### Strengths

The strengths of this study include the development of a questionnaire containing items that were not obviously associated with any specific psychological model or approach, evaluated through a systematic and detailed analytic procedure that included multiple tests of generalizability by using 2 large sample sizes. This procedure contributes to the literature by comparing the relative strengths of several groups of modifiable actions and the minimum frequency required for such actions to affect psychological health. An additional strength is that the results were mostly replicated although the second sample was obtained during a challenging period for the community characterized by social restrictions related to the impacts of the COVID-19 pandemic.

### Conclusions

In conclusion, these studies provide preliminary evidence for actions and factors strongly associated with psychological health and the frequency with which they should be performed. Future studies are planned to further explore the patterns of change in brief versions of the TYDQ, including samples of people seeking treatment for mental health conditions. We hope that these efforts will assist in the development of new psychological interventions and provide an evidence base for public mental health campaigns designed to promote good mental health and prevent the emergence of common mental disorders.

## Appendix

Multimedia Appendix 1 Items, primary and secondary cluster, item wording, and weekly score dispersion by frequency.

Multimedia Appendix 2 A heat map of the strength of association of Things You Do Questionnaire item weekly frequency with Patient Health Questionnaire 9-item, Generalized Anxiety Disorder 7-item, and Satisfaction With Life Scale outcomes, and the behavioral frequency thresholds associated with optimal mental health gains.

Multimedia Appendix 3 Exploratory factor analysis solution and assigned factor labels for items grouped with no item selection criteria (96 items), and item grouping under R2>5%, 10%, and 15% outcomes association criteria.

Multimedia Appendix 4 A heat map of the identified factor association with Patient Health Questionnaire 9-item, Generalized Anxiety Disorder 7-item, and Satisfaction With Life Scale outcomes, and the behavioral frequency thresholds associated with optimal mental health gains.

Multimedia Appendix 5 Exploratory factor analysis diagnostic statistics: item commonalities, factor eigenvalues, factor Cronbach α, and item mean intercorrelations.

Multimedia Appendix 6 Invariance test solutions examining dimensionality in study 1 sample.

Multimedia Appendix 7 Exploratory factor analysis parameter solution replications across key sample dimensions and methods of rotation methods.

Multimedia Appendix 8 Means, study 1 and study 2 sample group differences, and association of Things You Do Questionnaire items with each of the Patient Health Questionnaire 9-item, Generalized Anxiety Disorder 7-item, and Satisfaction With Life Scale outcomes.

Multimedia Appendix 9 Exploratory factor analysis solution and assigned factor labels for items grouped with no item selection criteria (96 items), and under R2>10% outcomes association criteria.

Multimedia Appendix 10 A heat map of the identified factor association with Patient Health Questionnaire 9-item, Generalized Anxiety Disorder 7-item, and Satisfaction With Life Scale outcomes across study 1 and study 2 samples.

Multimedia Appendix 11 Invariance test solutions examining dimensionality in study 2 sample.

Multimedia Appendix 12 Things You Do Questionnaire item lists.

## Abbreviations

CFA: confirmatory factor analysis
EFA: exploratory factor analysis
GAD-7: Generalized Anxiety Disorder 7-item
PHQ-9: Patient Health Questionnaire 9-item
SWLS: Satisfaction With Life Scale
TYDQ: Things You Do Questionnaire
