# Supplementary material for: Measures of Daily Activities Associated With Mental Health (Things You Do Questionnaire): Development of a Preliminary Psychometric Study and Replication Study
Source: JMIR Form Res. 2022 Jul 5;6(7):e38837. doi: 10.2196/38837 (PMC9297144; doi:10.2196/38837)
Supplement: Multimedia Appendix 1 [file formative_v6i7e38837_app1.docx]

**Multimedia Appendix 1.**

| **Table 1.** Items, primary and secondary cluster, item wording, and weekly score dispersion by frequency | | | | | | | | |
| --- | --- | --- | --- | --- | --- | --- | --- | --- |
| **Item order** | **Primary and secondary cluster** | **Item** | **TYDQ ^a^ weekly score, mean (SD)** | **Not at all (%)** | **One or two days/week (%)** | **Half the days/week (%)** | **Most days of the week (%)** | **Everyday (%)** |
| TYD^b^13 | Activity and meaning | I read, listened, or watched something I enjoyed | 2.72 (1.2) | 3.7 | 15.9 | 22.9 | 20 | 37.4 |
| TYD39 | Activity and meaning | I spent time doing something I think is important | 2.13 (1.3) | 11.9 | 25 | 23.3 | 17.9 | 21.8 |
| TYD88^c^ | Activity and enjoyable | I did something enjoyable | 2 (1.2) | 8.2 | 32.7 | 28.1 | 13.1 | 18 |
| TYD35^c^ | Activity and meaning | I spent time doing something I believed in | 1.89 (1.4) | 18.4 | 27.2 | 20.6 | 14.4 | 19.5 |
| TYD72^c^ | Activity and achieve goal | I did something to help me achieve my goals | 1.68 (1.3) | 20.8 | 31 | 21.6 | 12.5 | 14.1 |
| TYD44^c^ | Activity and laugh, fun | I had a good laugh or did something that was fun | 1.67 (1.2) | 14.1 | 37.4 | 26.9 | 10.8 | 10.9 |
| TYD34^c^ | Activity and interesting | I did a hobby or something that was of interest to me | 1.66 (1.3) | 20.6 | 32.5 | 21.8 | 10.5 | 14.6 |
| TYD66^c^ | Activity and satisfying | I did something that was very satisfying to me | 1.66 (1.2) | 16.3 | 36.6 | 23.2 | 11.9 | 11.9 |
| TYD78^c^ | Activity and improve quality of life | I did something to improve or maintain the quality of my life | 1.62 (1.3) | 20.5 | 34.4 | 21 | 10.6 | 13.5 |
| TYD89^c^ | Activity and avoid stagnant | I avoided being *stagnant* | 1.58 (1.4) | 29.8 | 24.7 | 18.5 | 11.6 | 15.4 |
| TYD23^c^ | Activity and meaning | I put effort and time into something I wanted to change | 1.55 (1.2) | 21 | 35.9 | 21.6 | 10.2 | 11.3 |
| TYD59 | Activity and meaning | I worked on an activity that was meaningful to me | 1.48 (1.3) | 30.6 | 26.7 | 19.3 | 11.2 | 12.2 |
| TYD71^c^ | Activity and learn new | I tried to learn something new | 1.48 (1.4) | 29.8 | 30 | 17.1 | 8.2 | 14.8 |
| TYD10 | Activity and meaning | I practiced a skill or did a hobby | 1.39 (1.3) | 31.3 | 29.7 | 18.4 | 9.6 | 11 |
| TYD32 | Cognitive and perspective | I tried to keep things in perspective | 2.49 (1.3) | 7.1 | 20.5 | 22.4 | 16.7 | 33.3 |
| TYD61^c^ | Cognitive and perspective | I allowed myself to be less than perfect | 2.38 (1.5) | 13.6 | 19.3 | 18.4 | 12.5 | 36.2 |
| TYD70^c^ | Cognitive and perspective | I kept a realistic perspective on things | 2.32 (1.3) | 8.7 | 20.7 | 25.4 | 19.7 | 25.4 |
| TYD17^c^ | Cognitive and future | Instead of worrying about the past, I focused on my preferred future | 1.86 (1.4) | 18.8 | 26.6 | 22.1 | 14.6 | 17.9 |
| TYD27 | Cognitive and challenging | I identified unhelpful thoughts and tried to replace them with more helpful ones | 1.71 (1.3) | 20.6 | 30.1 | 22.4 | 11.4 | 15.4 |
| TYD68^c^ | Cognitive and challenging | I stopped myself from thinking unhelpful or unrealistic thoughts | 1.69 (1.3) | 20.9 | 30.3 | 21.8 | 12.6 | 14.4 |
| TYD38 | Cognitive and problem solving | Instead of thinking about my worries, I focused on doing something about them | 1.59 (1.3) | 21.8 | 32.3 | 23 | 10.6 | 12.2 |
| TYD55 | Cognitive and challenging | I talked myself out of negative thinking | 1.56 (1.3) | 22.7 | 34.1 | 20.7 | 9.5 | 13 |
| TYD57 | Emotion regulation and coping | I fulfilled my responsibilities even though I did not want to | 2.76 (1.3) | 4.9 | 14.5 | 20 | 20.5 | 40.1 |
| TYD52 | Emotion regulation and realistic promises | I did not promise or commit to doing things I could not do | 2.4 (1.5) | 16.5 | 15.8 | 15.2 | 16 | 36.5 |
| TYD15 | Emotion regulation and pushing through | I pushed myself to do things that I did not feel like doing | 2.16 (1.3) | 9 | 27.1 | 25.2 | 16 | 22.7 |
| TYD20 | Emotion regulation and avoid chaos | I avoided chaos in my life | 2.14 (1.4) | 18 | 18.6 | 20.7 | 16.7 | 26.1 |
| TYD12 | Emotion regulation and no excuses | I did not make excuses | 2.13 (1.4) | 14.3 | 22.1 | 23.4 | 16.8 | 23.4 |
| TYD41 | Emotion regulation and patience | I practiced being patient | 2.1 (1.5) | 18.8 | 21.6 | 18.5 | 12.9 | 28.2 |
| TYD60^c^ | Emotion regulation and pushing through | I pushed myself to do things that were difficult or triggered some stress | 2.04 (1.3) | 11.8 | 27.6 | 25.1 | 15.4 | 20.1 |
| TYD43^c^ | Emotion regulation and pushing through | I made myself do something because I knew it would be beneficial | 1.96 (1.3) | 11.6 | 30.1 | 26.6 | 13.6 | 18 |
| TYD95 | Emotion regulation and expression | I was able to say no when I did not want to do something | 1.81 (1.4) | 21.1 | 29.2 | 17.9 | 11.4 | 20.4 |
| TYD92^c^ | Emotion regulation and expression | I expressed my feelings honestly, instead of suppressing them | 1.76 (1.3) | 19.1 | 31.1 | 21 | 12.5 | 16.3 |
| TYD22^c^ | Emotion regulation and coping | I dealt with feelings of frustration or impatience in a healthy way | 1.69 (1.3) | 20.9 | 29 | 23.4 | 13.6 | 13.1 |
| TYD25^c^ | Emotion regulation and coping | I dealt with things that were creating stress | 1.65 (1.3) | 18.7 | 34.4 | 23.5 | 10.2 | 13.1 |
| TYD28^c^ | Emotion regulation and pushing through | I faced a situation that was unpleasant but necessary | 1.63 (1.3) | 18.3 | 37.3 | 21.1 | 10.2 | 13.2 |
| TYD80^c^ | Environment | I did something to improve the quality of the physical environment | 1.45 (1.3) | 25.5 | 34.3 | 20.9 | 8.8 | 10.5 |
| TYD08^c^ | Gratitude and acceptance | I accepted a situation for what it is | 2.4 (1.3) | 8.2 | 21.8 | 22.7 | 16.6 | 30.7 |
| TYD65 | Gratitude and acceptance | I tried to accept things that I could not control or change | 2.35 (1.4) | 10.7 | 21.9 | 20.6 | 15.9 | 30.9 |
| TYD01^c^ | Gratitude and acceptance | I thought about things that I am grateful for | 1.87 (1.4) | 17.8 | 29.5 | 20.9 | 11.8 | 20 |
| TYD94^c^ | Gratitude and acceptance | I accepted my symptoms by allowing them to peak and pass | 1.85 (1.4) | 21.5 | 24.6 | 21.2 | 12.1 | 20.6 |
| TYD19 | Healthy routine and substance | I avoided illicit drugs and did not misuse medications | 3.57 (1.1) | 6.4 | 2.7 | 2.3 | 4.9 | 83.8 |
| TYD67^c^ | Healthy routine and hygiene | I had a bath or shower | 3.45 (1) | 1 | 5.5 | 10.3 | 13.9 | 69.3 |
| TYD03^c^ | Healthy routine and substance | I had an alcohol-free day | 2.86 (1.3) | 8.3 | 10.3 | 13.2 | 24.1 | 44.1 |
| TYD73^c^ | Healthy routine and chores | I did work or chores around where I live (eg, house, apartment) | 2.5 (1.3) | 5.1 | 24.4 | 21.6 | 13.5 | 35.4 |
| TYD85^c^ | Healthy routine and sunlight | I got regular exposure to sunlight (eg, 15 to 30 min) | 2.49 (1.4) | 8.3 | 20.4 | 21 | 14.9 | 35.4 |
| TYD96^c^ | Healthy routine and silence, solitude | I spent time in silence or solitude | 2.41 (1.5) | 12.8 | 21.1 | 16 | 12 | 38.1 |
| TYD26 | Healthy routine and hydration | I made sure I drank a healthy amount of water | 2.34 (1.4) | 15.1 | 16.8 | 18.2 | 18.7 | 31.2 |
| TYD30^c^ | Healthy routine and outside | I spent time outside | 2.34 (1.3) | 7.1 | 26.2 | 22.2 | 14 | 30.5 |
| TYD06^c^ | Healthy routine and excesses | I avoided unhealthy habits (eg, I chose not to have a drink or gamble) | 2.27 (1.5) | 19.2 | 15.6 | 14.8 | 19.8 | 30.6 |
| TYD64^c^ | Healthy routine and nutrition | I prepared and ate a healthy meal | 2.16 (1.3) | 12.1 | 20.6 | 26.4 | 21 | 19.9 |
| TYD24^c^ | Healthy routine and organized | I kept my home, living space, or workspace clean and organized | 1.92 (1.4) | 19.3 | 23.9 | 22.3 | 14.3 | 20.1 |
| TYD48^c^ | Healthy routine and mental well-being | I did things which are good for my mental well-being | 1.92 (1.3) | 13.4 | 30.2 | 25.7 | 12.6 | 18.1 |
| TYD05^c^ | Healthy routine and sleep | I went to bed and woke up at a regular time | 1.86 (1.5) | 27.5 | 15.7 | 18.7 | 19.4 | 18.7 |
| TYD09 | Healthy routine and finances | I created and stuck to my budget | 1.75 (1.6) | 35.1 | 13.2 | 15.3 | 14.5 | 21.9 |
| TYD36 | Healthy routine and social | I kept my use of social media and entertainment to a healthy level | 1.72 (1.5) | 29.2 | 21.1 | 18.2 | 11.5 | 20.1 |
| TYD02^c^ | Healthy routine and general | I kept a healthy daily routine | 1.71 (1.3) | 24.6 | 22.4 | 23.7 | 16.3 | 13.1 |
| TYD18^c^ | Healthy routine and electronics | I kept my use of electronic devices or games to a healthy level | 1.7 (1.5) | 30.1 | 21.1 | 17.8 | 10.6 | 20.5 |
| TYD91^c^ | Healthy routine and physical health | I did something to improve or maintain my physical health | 1.63 (1.4) | 25 | 28 | 21 | 10.7 | 15.4 |
| TYD84 | Healthy routine and nutrition | I avoided foods that caused emotional or physical problems, such as anxiety or indigestion | 1.55 (1.5) | 39.8 | 14.2 | 14.7 | 13.4 | 17.8 |
| TYD74^c^ | Healthy routine and exercise | I did some form of exercise (eg, swimming, going for a walk) | 1.55 (1.4) | 28.4 | 27.2 | 19.4 | 11 | 14 |
| TYD81 | Healthy routine and general satisfaction | I did something to improve my satisfaction with my life | 1.54 (1.3) | 21.8 | 35 | 22.1 | 9.5 | 11.6 |
| TYD49^c^ | Healthy routine and relax | I did something to help me relax (eg, slow breathing and stretching) | 1.52 (1.4) | 29 | 28.1 | 18.9 | 9.7 | 14.4 |
| TYD83 | Healthy routine and finances | I did something to improve or maintain my financial health | 1.46 (1.4) | 31 | 28.8 | 16 | 11.1 | 13.1 |
| TYD50 | Healthy routine and exercise | I did 30 min of exercise | 1.4 (1.4) | 33.9 | 25.9 | 18.3 | 10.3 | 11.6 |
| TYD46 | Healthy routine and electronics | I spent some time without the phone, television, or internet on | 1.23 (1.4) | 43.2 | 25.5 | 11.4 | 5.4 | 14.5 |
| TYD07 | Healthy routine and exercise | I planned or stuck to an exercise routine | 1.06 (1.3) | 50.9 | 18.1 | 14 | 7.9 | 9 |
| TYD21^c^ | Healthy routine and sleep | I kept a relaxing bedtime routine, that did not involve watching videos or checking social media | 1 (1.4) | 55.7 | 16.7 | 10.3 | 6.8 | 10.5 |
| TYD45^c^ | Plan and personal responsibility | I took responsibility for the direction of my life | 2.4 (1.5) | 13.1 | 19.8 | 17.5 | 13 | 36.6 |
| TYD04^c^ | Plan and organize | I took steps to organize what I did each day | 2.01 (1.4) | 18.6 | 21.5 | 21.9 | 16.9 | 21.1 |
| TYD54^c^ | Plan and future | I had something to look forward to | 1.86 (1.4) | 16.5 | 33.1 | 19.3 | 9.5 | 21.5 |
| TYD14 | Plan and future | I gave myself things to look forward to | 1.77 (1.4) | 19.1 | 30.9 | 21.6 | 11.2 | 17.3 |
| TYD40^c^ | Plan and realistic goals | I set realistic and achievable goals | 1.68 (1.4) | 23.6 | 26.7 | 22.2 | 13 | 14.5 |
| TYD47^c^ | Plan and execute | I made a plan and stuck to it | 1.68 (1.3) | 19.9 | 29.3 | 25.1 | 14.7 | 11 |
| TYD11 | Problem solving | I took steps to solve a problem that was affecting me | 1.71 (1.3) | 17.2 | 33.6 | 24.7 | 10.4 | 14.1 |
| TYD42 | Problem solving | I broke a large problem into smaller, more manageable steps | 1.41 (1.3) | 31.6 | 28.1 | 19.8 | 8.7 | 11.9 |
| TYD53 | Respect and others | I treated others with respect | 3.55 (0.8) | 0.8 | 3.4 | 7.8 | 16.5 | 71.6 |
| TYD16^c^ | Respect and self | I treated myself with respect | 2.17 (1.4) | 14.7 | 22.3 | 20.9 | 15 | 27.1 |
| TYD90^c^ | Respect and reflection | I took time to reflect on myself and how I felt | 1.95 (1.5) | 19.6 | 25.7 | 18.8 | 11.7 | 24.2 |
| TYD69^c^ | Respect and self | I praised myself when I did something well | 1.34 (1.3) | 33 | 30.5 | 17 | 8.4 | 11 |
| TYD75 | Social and social media | I sent a personal email, text message, or made a post on social media to someone | 2.66 (1.3) | 5.9 | 18.2 | 20.2 | 15.8 | 39.9 |
| TYD51^c^ | Social and praise others | I encouraged or praised someone | 2.55 (1.2) | 4.8 | 17.6 | 25.4 | 22.5 | 29.8 |
| TYD37^c^ | Social and help others | I did something to help others | 2.28 (1.3) | 7.3 | 23.9 | 27.2 | 16.5 | 25 |
| TYD56^c^ | Social and kindness to others | I did something kind for someone else | 2.23 (1.2) | 5.6 | 26.7 | 28.9 | 16.6 | 22.2 |
| TYD79 | Social and help others | I did something to improve the quality of other people’s lives | 2.14 (1.3) | 10.2 | 25.6 | 25.7 | 16.4 | 22.1 |
| TYD31^c^ | Social and talking | I talked about my day with a friend or a family member | 2.11 (1.4) | 14.7 | 26.7 | 18.7 | 12.7 | 27.1 |
| TYD33^c^ | Social and talking | I had a meaningful conversation with someone | 1.88 (1.3) | 11.7 | 34.1 | 25.4 | 11.9 | 16.9 |
| TYD87 | Social and improve relationships | I did something to improve my relationships with people who are important to me | 1.8 (1.3) | 15.1 | 32.5 | 25.2 | 12.1 | 15.1 |
| TYD62 | Social and positive people | I aimed to spend time with positive people | 1.76 (1.4) | 22.3 | 26.1 | 22 | 12.1 | 17.5 |
| TYD29^c^ | Social and positive people | I socialized with positive people | 1.68 (1.2) | 16 | 34.2 | 26.8 | 11.6 | 11.3 |
| TYD63 | Social and talking | I talked with a friend or family member on the phone | 1.53 (1.3) | 21.4 | 39.2 | 18.1 | 7.4 | 13.9 |
| TYD93 | Social and improve belonging | I did something to improve my sense of belonging | 1.13 (1.2) | 38.2 | 31.1 | 16.8 | 7.1 | 6.9 |
| TYD58 | Social and positive people | I arranged to see friends | 1.03 (1) | 31.5 | 44.3 | 16.9 | 4.7 | 2.6 |
| TYD77^c^ | Values and spiritual | I acted with integrity and dignity | 3.17 (1.1) | 2.9 | 6.8 | 14.2 | 22.4 | 53.7 |
| TYD76^c^ | Values and spiritual | I acted in a way that is consistent with my personal values | 2.99 (1.2) | 3.8 | 10.3 | 16 | 22.8 | 47.2 |
| TYD82^c^ | Values and spiritual | I did something to help me live my *ideal* life | 1.42 (1.3) | 28.7 | 32.4 | 18.2 | 9.3 | 11.4 |
| TYD86^c^ | Values and spiritual | I did something to improve or maintain my spiritual well-being | 1.13 (1.4) | 46 | 23.8 | 12.6 | 6.4 | 11.2 |
| ^a^ TYDQ: Things You Do Questionnaire.  ^b^ TYD: Things You Do.  ^c^ Items included in Study 2. | | | | | | | | |
