# Supplementary material for: Measures of Daily Activities Associated With Mental Health (Things You Do Questionnaire): Development of a Preliminary Psychometric Study and Replication Study
Source: JMIR Form Res. 2022 Jul 5;6(7):e38837. doi: 10.2196/38837 (PMC9297144; doi:10.2196/38837)
Supplement: Multimedia Appendix 2 [file formative_v6i7e38837_app2.docx]

**Multimedia Appendix 2**

**Table 1.** A heat map of the strength of association of TYDQ item weekly frequency with PHQ-9, GAD-7 and SWLS outcomes, and the behavioural frequency thresholds associated with optimal mental health gains.

| Item  order | Primary/Secondary Clusters | Item | TYD Mean |  | Not at  all | 1-2 day \| Week | Half the week | Most  days | Daily |  |  | Not at  all | 1-2 day \| Week | Half the week | Most  days | Daily |  | |  | | Not at  all | 1-2 day \| Week | Half the week | Most  days | Daily | |  | |  |
| --- | --- | --- | --- | --- | --- | --- | --- | --- | --- | --- | --- | --- | --- | --- | --- | --- | --- | --- | --- | --- | --- | --- | --- | --- | --- | --- | --- | --- | --- |
|  |  |  |  |  | (0) | (1) | (2) | (3) | (4) |  |  | (0) | (1) | (2) | (3) | (4) |  | |  | | (0) | (1) | (2) | (3) | (4) | |  | |  |
|  |  |  |  |  | Item → PHQ-9 mean scores | | | | | *R^2^* |  | Item → GAD-7 mean scores | | | | | *R^2^* |  | | Item → SWLS mean scores | | | | | | *R^2^* | |  |  |
|  |  |  |  |  |  | | | | |  |  |  | | | | |  |  | |  | | | | | |  | |  |  |
| TYD88* | Activity/Enjoyable | I did something enjoyable | 2 |  | 17.1 | 12.9 | 10.4 | 8.4 | 6.5 | 18.8% |  | 13.1 | 10.4 | 8.8 | 7.1 | 5.2 | 14.6% | |  | | 12.4 | 16 | 18.6 | 20.7 | 23.8 | | 17.5% | |  |
| TYD66* | Activity/Satisfying | I did something that was very satisfying to me | 1.66 |  | 15.6 | 12.1 | 9.3 | 8 | 6 | 17.8% |  | 11.9 | 9.9 | 7.8 | 7 | 5.1 | 12.1% | |  | | 13.4 | 17 | 19.9 | 21.6 | 23.8 | | 16.3% | |  |
| TYD44* | Activity/Laugh, fun | I had a good laugh or did something that was fun | 1.67 |  | 15.4 | 11.9 | 9.8 | 7.7 | 6.4 | 14.3% |  | 12 | 9.8 | 8.1 | 6.4 | 5.2 | 11.7% | |  | | 13.5 | 16.9 | 19.6 | 22.1 | 23.4 | | 14.4% | |  |
| TYD35* | Activity/Meaning | I spent time doing something I believed in | 1.89 |  | 14.5 | 12.2 | 10.2 | 8.5 | 7.6 | 12.5% |  | 11 | 9.8 | 8.5 | 7.4 | 6.5 | 7.4% | |  | | 14.4 | 16.6 | 19 | 21.1 | 22.5 | | 13.5% | |  |
| TYD89* | Activity/Avoid stagnant | I avoided being 'stagnant' | 1.58 |  | 13.4 | 12.2 | 9.9 | 8.4 | 6.6 | 13.0% |  | 10.3 | 10 | 8.2 | 7.4 | 5.8 | 7.6% | |  | | 16 | 17.1 | 19.2 | 20.6 | 23 | | 10.0% | |  |
| TYD39 | Activity/Meaning | I spent time doing something I think is important | 2.13 |  | 14.9 | 12.7 | 10.6 | 8.7 | 8.1 | 11.1% |  | 10.9 | 10 | 8.9 | 7.5 | 7 | 5.5% | |  | | 13.3 | 16.1 | 18.6 | 20.9 | 21.9 | | 13.2% | |  |
| TYD78* | Activity/Improve quality of life | I did something to improve or maintain the quality of my life | 1.62 |  | 13.7 | 12 | 9.5 | 8.2 | 7.3 | 10.4% |  | 10.6 | 9.8 | 8 | 7 | 6.2 | 6.9% | |  | | 14.6 | 17.3 | 20 | 21.5 | 22.5 | | 11.6% | |  |
| TYD72* | Activity/Achieve goal | I did something to help me achieve my goals | 1.68 |  | 13.8 | 11.8 | 10.1 | 8.2 | 7.6 | 9.6% |  | 10.4 | 9.4 | 8.5 | 7.1 | 7 | 4.3% | |  | | 14.5 | 17.4 | 19.3 | 21.9 | 22.4 | | 11.9% | |  |
| TYD59 | Activity/Meaning | I worked on an activity that was meaningful to me | 1.48 |  | 13.4 | 11.4 | 9.4 | 8.2 | 7.4 | 9.9% |  | 10.4 | 9.3 | 8 | 7.3 | 6.5 | 5.4% | |  | | 15.4 | 18 | 19.9 | 21.4 | 22.1 | | 9.4% | |  |
| TYD34* | Activity/Interesting | I did a hobby or something that was of interest to me | 1.66 |  | 14.3 | 11.3 | 9.7 | 8.5 | 7.7 | 9.7% |  | 11.1 | 9.5 | 8.1 | 7 | 6.3 | 7.3% | |  | | 15.7 | 17.7 | 19.5 | 20.1 | 21.3 | | 5.5% | |  |
| TYD13 | Activity/Meaning | I read, listened, or watched something I enjoyed | 2.72 |  | 15.8 | 13.6 | 11.5 | 10.3 | 8.9 | 7.8% |  | 12.2 | 11.2 | 9.5 | 8.5 | 7.2 | 6.9% | |  | | 13.5 | 15.8 | 17.3 | 19 | 20.4 | | 6.3% | |  |
| TYD10 | Activity/Meaning | I practiced a skill or did a hobby | 1.39 |  | 12.9 | 11 | 9.5 | 9.1 | 8.1 | 5.7% |  | 10.3 | 9.1 | 8.1 | 7.4 | 6.6 | 4.5% | |  | | 16.3 | 18.5 | 19.7 | 19.5 | 21.2 | | 3.9% | |  |
| TYD23* | Activity/Meaning | I put effort and time into something I wanted to change | 1.55 |  | 12.6 | 11.6 | 10.1 | 7.9 | 8.8 | 4.4% |  | 10 | 9.1 | 8.6 | 7.2 | 7.5 | 2.2% | |  | | 15.7 | 17.7 | 19.6 | 21.6 | 20.6 | | 5.2% | |  |
| TYD71* | Activity/Learn new | I tried to learn something new | 1.48 |  | 12.5 | 10.4 | 9.9 | 9.5 | 10 | 1.8% |  | 9.9 | 8.6 | 8.3 | 8 | 8 | 1.2% | |  | | 16.4 | 18.7 | 19.8 | 19.7 | 20 | | 2.6% | |  |
|  |  |  |  |  |  |  |  |  |  |  |  |  |  |  |  |  |  | |  | |  |  |  |  |  | |  | |  |
| TYD70* | Cognitive/Perspective | I kept a realistic perspective on things | 2.32 |  | 16.4 | 14 | 11.2 | 8.7 | 7.5 | 18.1% |  | 13.6 | 11.8 | 9.4 | 6.9 | 5.6 | 21.1% | |  | | 13.3 | 15.5 | 18.1 | 20.2 | 21.7 | | 12.2% | |  |
| TYD17* | Cognitive/Future | Instead of worrying about the past, I focused on my preferred future | 1.86 |  | 14.5 | 12.3 | 10.4 | 8.4 | 7.1 | 13.8% |  | 11.5 | 10.2 | 8.7 | 6.9 | 5.5 | 12.9% | |  | | 14.4 | 16.4 | 18.9 | 21.5 | 22.6 | | 14.2% | |  |
| TYD32 | Cognitive/Perspective | I tried to keep things in perspective | 2.49 |  | 16 | 13.4 | 11.3 | 9.1 | 8.4 | 12.1% |  | 12.5 | 11.3 | 9.4 | 7.5 | 6.7 | 11.6% | |  | | 12.5 | 15.9 | 17.4 | 20.2 | 21.2 | | 11.4% | |  |
| TYD68* | Cognitive/Challenging | I stopped myself from thinking unhelpful or unrealistic thoughts | 1.69 |  | 14.2 | 11.9 | 9.8 | 8.2 | 7.5 | 11.2% |  | 11.4 | 9.8 | 8.3 | 6.7 | 5.6 | 11.1% | |  | | 15.5 | 17.3 | 19.2 | 21.4 | 21.4 | | 7.7% | |  |
| TYD38 | Cognitive/Problem solving | Instead of thinking about my worries, I focused on doing something about them | 1.59 |  | 13.6 | 11.8 | 9.8 | 7.6 | 7.3 | 10.2% |  | 10.5 | 9.9 | 8.2 | 6.4 | 6.2 | 7.2% | |  | | 15 | 17.3 | 19.8 | 22.3 | 21.9 | | 10.0% | |  |
| TYD61* | Cognitive/Perspective | I allowed myself to be less than perfect | 2.38 |  | 14.7 | 12.2 | 11 | 9.1 | 9.1 | 7.7% |  | 12.5 | 10.3 | 9.2 | 7.4 | 6.9 | 11.0% | |  | | 15.5 | 17.1 | 18.3 | 19.7 | 19.9 | | 4.1% | |  |
| TYD55 | Cognitive/Challenging | I talked myself out of negative thinking | 1.56 |  | 13.3 | 11.3 | 9.9 | 8.3 | 8.5 | 5.9% |  | 10.4 | 9.3 | 8.2 | 7.1 | 7.1 | 4.0% | |  | | 15.8 | 17.8 | 19.6 | 21.2 | 21 | | 5.5% | |  |
| TYD27 | Cognitive/Challenging | I identified unhelpful thoughts and tried to replace them with more helpful ones | 1.71 |  | 12.8 | 11.9 | 10 | 9.1 | 8.3 | 5.5% |  | 10 | 9.8 | 8.2 | 7.7 | 6.9 | 3.8% | |  | | 15.8 | 17.4 | 19.4 | 20.9 | 20.9 | | 5.6% | |  |
|  |  |  |  |  |  |  |  |  |  |  |  |  |  |  |  |  |  | |  | |  |  |  |  |  | |  | |  |
| TYD22* | Emotion Regulation/Coping | I dealt with feelings of frustration or impatience in a healthy way | 1.69 |  | 14.7 | 11.6 | 10 | 8 | 7.3 | 12.6% |  | 11.5 | 9.7 | 8.3 | 6.8 | 5.6 | 10.7% | |  | | 14.6 | 17.3 | 19.7 | 21.4 | 21.6 | | 9.8% | |  |
| TYD12 | Emotion Regulation/No excuses | I didn't make excuses | 2.13 |  | 14.7 | 12.1 | 10.8 | 8.8 | 8.7 | 8.7% |  | 11.3 | 10.2 | 9 | 7.2 | 7 | 7.2% | |  | | 14.9 | 17 | 18.4 | 20.9 | 20.2 | | 5.6% | |  |
| TYD92* | Emotion Regulation/Expression | I expressed my feelings honestly, instead of suppressing them | 1.76 |  | 13.5 | 11.4 | 10.5 | 8.7 | 8.6 | 5.7% |  | 10.8 | 9.3 | 8.6 | 7.3 | 7 | 4.7% | |  | | 15 | 17.5 | 19.2 | 21.3 | 21.3 | | 8.0% | |  |
| TYD20 | Emotion Regulation/Avoid chaos | I avoided chaos in my life | 2.14 |  | 13.3 | 12 | 11.1 | 9.4 | 8.9 | 5.5% |  | 10.9 | 10.3 | 9.1 | 7.6 | 6.9 | 6.8% | |  | | 15.9 | 17.7 | 18 | 19.9 | 20 | | 3.7% | |  |
| TYD95 | Emotion Regulation/Expression | I was able to say no when I did not want to do something | 1.81 |  | 13.4 | 11.1 | 10.3 | 9.4 | 8.9 | 5.0% |  | 10.8 | 9.3 | 8.5 | 8 | 6.8 | 5.2% | |  | | 15.7 | 17.9 | 19.2 | 20.4 | 20.4 | | 4.6% | |  |
| TYD25* | Emotion Regulation/Coping | I dealt with things that were creating stress | 1.65 |  | 13.9 | 11.1 | 9.7 | 8.6 | 9.2 | 4.9% |  | 10.5 | 9.2 | 8.1 | 7.5 | 7.9 | 2.3% | |  | | 14.6 | 17.8 | 19.9 | 21.4 | 20.6 | | 6.7% | |  |
| TYD41 | Emotion Regulation/Patience | I practiced being patient | 2.1 |  | 12.5 | 12.1 | 10.3 | 8.8 | 9.8 | 2.9% |  | 10.3 | 9.8 | 8.8 | 7.3 | 7.7 | 3.3% | |  | | 16 | 17.1 | 18.9 | 20.7 | 19.7 | | 3.5% | |  |
| TYD43* | Emotion Regulation/Pushing through | I made myself do something because I knew it would be beneficial | 1.96 |  | 13.5 | 12 | 9.9 | 8.9 | 9.7 | 3.6% |  | 10.4 | 9.5 | 8.2 | 7.6 | 8.3 | 1.6% | |  | | 15.1 | 17.1 | 19.1 | 20.4 | 20.3 | | 4.6% | |  |
| TYD52 | Emotion Regulation/Realistic promises | I didn't promise or commit to doing things I couldn't do | 2.4 |  | 12.1 | 12 | 10.9 | 10.5 | 9.7 | 2.0% |  | 9.7 | 10.1 | 9.2 | 8.8 | 7.7 | 2.3% | |  | | 16.5 | 17.9 | 18.6 | 19.1 | 19.2 | | 1.4% | |  |
| TYD28* | Emotion Regulation/Pushing through | I faced a situation that was unpleasant but necessary | 1.63 |  | 10.8 | 10.4 | 10.6 | 10.9 | 12.2 | 0.4% |  | 8.1 | 8.3 | 8.9 | 9.5 | 10.4 | 1.6% | |  | | 17.5 | 18.8 | 19.1 | 19 | 17.5 | | 0.0% | |  |
| TYD60* | Emotion Regulation/Pushing through | I pushed myself to do things that were difficult or triggered some stress | 2.04 |  | 11.6 | 10.6 | 10.2 | 10.2 | 11.7 | 0.0% |  | 8.7 | 8.2 | 8.4 | 8.6 | 10.4 | 1.1% | |  | | 16.6 | 18 | 19.2 | 19.2 | 18.8 | | 0.7% | |  |
| TYD57 | Emotion Regulation/Coping | I fulfilled my responsibilities even though I didn't want to | 2.76 |  | 11.8 | 11.5 | 11.1 | 10.2 | 10.6 | 0.3% |  | 8.5 | 8.7 | 9.1 | 8.6 | 8.9 | <0.1%* | |  | | 15.3 | 17.3 | 18.3 | 18.7 | 19.2 | | 1.3% | |  |
| TYD15 | Emotion Regulation/Pushing through | I pushed myself to do things that I didn’t feel like doing | 2.16 |  | 12.5 | 11.2 | 9.5 | 10 | 11.5 | 0.1% |  | 9.1 | 8.9 | 8.1 | 8.3 | 9.8 | 0.2% | |  | | 15.9 | 17.5 | 19.5 | 19.5 | 18.5 | | 0.8% | |  |
|  |  |  |  |  |  |  |  |  |  |  |  |  |  |  |  |  |  | |  | |  |  |  |  |  | |  | |  |
| TYD80* | Environment | I did something to improve the quality of the physical environment | 1.45 |  | 12.6 | 11.3 | 9.8 | 8.3 | 9 | 3.9% |  | 9.8 | 9.1 | 8.2 | 7.2 | 7.8 | 1.8% | |  | | 16.1 | 18 | 19.9 | 21 | 20.9 | | 4.8% | |  |
|  |  |  |  |  |  |  |  |  |  |  |  |  |  |  |  |  |  | |  | |  |  |  |  |  | |  | |  |
| TYD65 | Gratitude/Acceptance | I tried to accept things that I couldn’t control or change | 2.35 |  | 14.6 | 12.6 | 11.1 | 9.3 | 8.8 | 7.9% |  | 11.7 | 10.7 | 9.3 | 7.6 | 6.8 | 9.2% | |  | | 14.5 | 16.1 | 18.3 | 20.3 | 20.6 | | 7.6% | |  |
| TYD08* | Gratitude/Acceptance | I accepted a situation for what it is | 2.4 |  | 15.3 | 12.7 | 11 | 9 | 9.2 | 7.4% |  | 12.8 | 10.7 | 9.3 | 7.3 | 7 | 9.6% | |  | | 13.4 | 16.2 | 18.5 | 20.4 | 20.3 | | 7.3% | |  |
| TYD01* | Gratitude/Acceptance | I thought about things that I am grateful for | 1.87 |  | 12.9 | 12.4 | 10.4 | 8.9 | 8.3 | 6.8% |  | 10.1 | 10 | 8.7 | 7.6 | 7 | 4.2% | |  | | 14.9 | 16.6 | 18.5 | 21.5 | 22.4 | | 12.3% | |  |
| TYD94* | Gratitude/Acceptance | I accepted my symptoms by allowing them to peak and pass | 1.85 |  | 11.9 | 11 | 11 | 9.9 | 9.8 | 1.2% |  | 9.4 | 9.1 | 9 | 8.4 | 7.8 | 1.0% | |  | | 16.9 | 18.2 | 18.4 | 20 | 19.6 | | 1.6% | |  |
|  |  |  |  |  |  |  |  |  |  |  |  |  |  |  |  |  |  | |  | |  |  |  |  |  | |  | |  |
| TYD48* | Healthy Routine/Mental wellbeing | I did things which are good for my mental wellbeing | 1.92 |  | 14.8 | 12.6 | 10.5 | 8 | 7.1 | 14.1% |  | 11.6 | 10.2 | 8.9 | 6.5 | 5.9 | 10.8% | |  | | 14.4 | 16.3 | 18.8 | 21.5 | 22.3 | | 12.1% | |  |
| TYD02* | Healthy Routine/General | I kept a healthy daily routine | 1.71 |  | 14.7 | 12.1 | 9.8 | 8.2 | 6.8 | 15.8% |  | 10.9 | 9.9 | 8.3 | 7.3 | 6 | 8.2% | |  | | 14.6 | 17.2 | 19.6 | 21.1 | 22.2 | | 11.8% | |  |
| TYD81 | Healthy Routine/General satisfaction | I did something to improve my satisfaction with my life | 1.54 |  | 13.6 | 11.8 | 9.6 | 7.8 | 7 | 10.4% |  | 10.4 | 9.6 | 8 | 7.1 | 6.1 | 6.0% | |  | | 14.9 | 17.3 | 19.7 | 22.6 | 22.8 | | 12.0% | |  |
| TYD05* | Healthy Routine/Sleep | I went to bed and woke up at a regular time | 1.86 |  | 14.4 | 11.5 | 9.8 | 8.9 | 7.9 | 12.5% |  | 11.1 | 9.5 | 8 | 7.8 | 7 | 6.6% | |  | | 15.3 | 17.5 | 19.4 | 20.3 | 20.9 | | 7.4% | |  |
| TYD64* | Healthy Routine/Nutrition | I prepared and ate a healthy meal | 2.16 |  | 14.7 | 13 | 10.7 | 9.1 | 8 | 10.8% |  | 11 | 10.4 | 8.7 | 8.1 | 6.8 | 5.7% | |  | | 14.7 | 16.9 | 18.2 | 20.1 | 20.9 | | 6.4% | |  |
| TYD91* | Healthy Routine/Physical health | I did something to improve or maintain my physical health | 1.63 |  | 13.4 | 11.6 | 10.1 | 8.5 | 7.9 | 8.2% |  | 10.4 | 9.5 | 8.5 | 7.1 | 6.7 | 5.1% | |  | | 15.7 | 18.1 | 19.5 | 20.2 | 21 | | 5.3% | |  |
| TYD30* | Healthy Routine/Outside | I spent time outside | 2.34 |  | 14.5 | 12.8 | 10.7 | 9.8 | 8.7 | 7.3% |  | 11.3 | 10.2 | 8.8 | 8.5 | 7.2 | 5.2% | |  | | 15.4 | 16.6 | 18.7 | 19.3 | 20.2 | | 4.2% | |  |
| TYD85* | Healthy Routine/Sunlight | I got regular exposure to sunlight (e.g., 15-30 mins) | 2.49 |  | 14.7 | 12.8 | 10.9 | 10.2 | 9 | 6.9% |  | 11.5 | 10.2 | 8.8 | 8.7 | 7.4 | 4.8% | |  | | 14.8 | 16.8 | 18.1 | 19 | 20.2 | | 4.7% | |  |
| TYD74* | Healthy Routine/Exercise | I did some form of exercise (e.g. swimming, went for a walk, etc) | 1.55 |  | 13.2 | 11.3 | 9.3 | 8.8 | 8.5 | 6.5% |  | 10.3 | 9.3 | 7.9 | 7.4 | 7.3 | 3.8% | |  | | 16 | 18.4 | 19.7 | 20.1 | 20.6 | | 4.2% | |  |
| TYD07 | Healthy Routine/Exercise | I planned or stuck to an exercise routine | 1.06 |  | 12.4 | 10.7 | 9 | 7.8 | 8 | 6.4% |  | 9.8 | 9 | 7.6 | 6.8 | 7.1 | 3.5% | |  | | 16.9 | 19 | 20.5 | 20.4 | 21.1 | | 4.2% | |  |
| TYD18* | Healthy Routine/Electronics | I kept my use of electronic devices or games to a healthy level | 1.7 |  | 13 | 10.8 | 10.2 | 9.8 | 8.5 | 5.4% |  | 10.3 | 9 | 8.5 | 8.1 | 7.1 | 3.8% | |  | | 16.2 | 18.4 | 18.9 | 20 | 20.5 | | 4.1% | |  |
| TYD67* | Healthy Routine/Hygiene | I had a bath or shower | 3.45 |  | 16.2 | 15.4 | 13.1 | 11.8 | 9.8 | 6.1% |  | 11.7 | 11.5 | 10.1 | 9.3 | 8.3 | 2.6% | |  | | 13 | 15.1 | 16.2 | 16.7 | 19.5 | | 4.0% | |  |
| TYD50 | Healthy Routine/Exercise | I did 30 minutes of exercise | 1.4 |  | 12.7 | 11.1 | 9.5 | 8.7 | 8.3 | 5.6% |  | 9.9 | 9.2 | 8 | 7.5 | 7 | 3.2% | |  | | 16.4 | 18.5 | 20 | 19.5 | 20.8 | | 3.8% | |  |
| TYD73* | Healthy Routine/Chores | I did work or chores around where I live (e.g., house, apartment, etc) | 2.5 |  | 14.6 | 12.9 | 10.4 | 9.8 | 9.4 | 4.9% |  | 11.4 | 10 | 8.3 | 8.1 | 8.2 | 2.1% | |  | | 13.2 | 16.4 | 18.7 | 19.2 | 20.2 | | 5.3% | |  |
| TYD24* | Healthy Routine/Organised | I kept my home, living space, or workspace clean and organised | 1.92 |  | 13.8 | 11.5 | 9.6 | 9.5 | 9.3 | 4.9% |  | 10.6 | 9.2 | 7.9 | 8.2 | 8.1 | 2.1% | |  | | 15.2 | 17.4 | 19.5 | 20.5 | 20.2 | | 5.2% | |  |
| TYD83 | Healthy Routine/Finances | I did something to improve or maintain my financial health | 1.46 |  | 12.5 | 11.2 | 9.5 | 9.3 | 8.8 | 4.0% |  | 9.7 | 9.2 | 7.9 | 8 | 7.6 | 1.9% | |  | | 16 | 18.3 | 19.8 | 20.4 | 21.3 | | 5.6% | |  |
| TYD36 | Healthy Routine/Social | I kept my use of social media and entertainment to a healthy level | 1.72 |  | 12.8 | 11.1 | 9.9 | 9.6 | 8.9 | 4.4% |  | 10.1 | 9.4 | 8.2 | 7.9 | 7.3 | 3.4% | |  | | 16.3 | 18.1 | 19.7 | 19.4 | 20.2 | | 3.4% | |  |
| TYD26 | Healthy Routine/Hydration | I made sure I drank a healthy amount of water | 2.34 |  | 13.4 | 12 | 10.7 | 10.3 | 9.3 | 4.2% |  | 10.4 | 9.6 | 9.1 | 8.7 | 7.5 | 2.9% | |  | | 15.5 | 17.4 | 19.2 | 19.2 | 19.6 | | 2.9% | |  |
| TYD21* | Healthy Routine/Sleep | I kept a relaxing bedtime routine, that did not involve watching videos or checking social media | 1 |  | 11.9 | 10.4 | 9.7 | 8.4 | 8.2 | 4.0% |  | 9.6 | 8.6 | 8.3 | 7.2 | 6.5 | 3.2% | |  | | 17.4 | 18.6 | 20.1 | 20.5 | 20.6 | | 2.6% | |  |
| TYD49* | Healthy Routine/Relax | I did something to help me relax (e.g., slow breathing, stretching etc) | 1.52 |  | 12.2 | 11.4 | 10 | 9.1 | 9.1 | 3.0% |  | 9.5 | 9.3 | 8.3 | 7.7 | 7.7 | 1.6% | |  | | 16.5 | 17.8 | 19.5 | 21 | 20.4 | | 3.9% | |  |
| TYD46 | Healthy Routine/Electronics | I spent some time without the phone, TV, or internet on | 1.23 |  | 11.7 | 11.1 | 10 | 9.9 | 8.3 | 2.9% |  | 9.5 | 9.1 | 8.2 | 8.1 | 7 | 2.2% | |  | | 17.2 | 18.4 | 19.5 | 19.6 | 20.9 | | 2.8% | |  |
| TYD84 | Healthy Routine/Nutrition | I avoided foods that caused emotional or physical problems, such as anxiety or indigestion | 1.55 |  | 11.8 | 11.9 | 10.4 | 9.2 | 9.3 | 2.6% |  | 9.4 | 9.7 | 8.9 | 7.7 | 7.5 | 1.8% | |  | | 17.6 | 17.6 | 19.6 | 19.9 | 19.1 | | 1.0% | |  |
| TYD06* | Healthy Routine/Excesses | I avoided unhealthy habits (e.g., I chose not to have a drink, or gamble, etc) | 2.27 |  | 13.2 | 11.5 | 9.9 | 9.8 | 10.2 | 2.5% |  | 10.3 | 9.3 | 8.2 | 8.3 | 8.3 | 1.5% | |  | | 16.3 | 17.9 | 19.2 | 19.8 | 18.9 | | 1.6% | |  |
| TYD19 | Healthy Routine/Substance | I avoided illicit drugs, and did not misuse medications | 3.57 |  | 13.8 | 13.9 | 12.8 | 12.7 | 10.3 | 2.6% |  | 10.8 | 11 | 10.2 | 10.2 | 8.5 | 1.6% | |  | | 16.1 | 16.5 | 17.1 | 16.6 | 18.8 | | 1.1% | |  |
| TYD09 | Healthy Routine/Finances | I created and stuck to my budget | 1.75 |  | 11.9 | 11.6 | 10.4 | 9.5 | 9.8 | 2.0% |  | 9.5 | 9.4 | 8.8 | 8 | 8.1 | 1.0% | |  | | 17.1 | 17.9 | 19.3 | 19.6 | 19.6 | | 1.9% | |  |
| TYD03* | Healthy Routine/Substance | I had an alcohol free day | 2.86 |  | 11.1 | 10.2 | 9.5 | 9.9 | 11.8 | 0.6% |  | 9.2 | 8 | 7.8 | 8.4 | 9.6 | 0.5% | |  | | 17.8 | 18.8 | 20 | 19.7 | 17.3 | | 0.3% | |  |
| TYD96* | Healthy Routine/Silence, solitude | I spent time in Silence/ solitude | 2.41 |  | 11.1 | 10.1 | 10.1 | 11.1 | 11.3 | 0.3% |  | 9.6 | 8.3 | 8.5 | 9.3 | 8.8 | 0.0% | |  | | 17.8 | 19.5 | 18.9 | 18.5 | 17.9 | | 0.2% | |  |
|  |  |  |  |  |  |  |  |  |  |  |  |  |  |  |  |  |  | |  | |  |  |  |  |  | |  | |  |
| TYD54* | Plan/Future | I had something to look forward to | 1.86 |  | 15.9 | 12.5 | 10 | 7.5 | 6.4 | 22.5% |  | 12.1 | 10.1 | 8.5 | 6.6 | 5.6 | 14.4% | |  | | 12.8 | 16.1 | 19.2 | 22.2 | 24.2 | | 25.9% | |  |
| TYD14 | Plan/Future | I gave myself things to look forward to | 1.77 |  | 14.7 | 12.6 | 9.7 | 8 | 6.5 | 17.4% |  | 11.1 | 10.2 | 8.2 | 7 | 5.7 | 10.8% | |  | | 13.7 | 16.4 | 19.8 | 21.9 | 23.3 | | 18.7% | |  |
| TYD40* | Plan/Realistic goals | I set realistic and achievable goals | 1.68 |  | 13.6 | 12.3 | 9.6 | 8 | 7.4 | 11.6% |  | 10.5 | 10.1 | 8.2 | 6.8 | 6.3 | 7.5% | |  | | 14.8 | 16.6 | 19.8 | 22.3 | 22.3 | | 13.5% | |  |
| TYD45* | Plan/Personal responsibility | I took responsibility for the direction of my life | 2.4 |  | 14.2 | 12.9 | 11.2 | 9.7 | 8.6 | 9.4% |  | 11 | 10.5 | 9.5 | 8.1 | 7 | 7.2% | |  | | 13.8 | 16 | 17.7 | 20 | 21.2 | | 11.8% | |  |
| TYD47* | Plan/Execute | I made a plan and stuck to it | 1.68 |  | 13.8 | 12 | 10 | 7.8 | 7.7 | 10.0% |  | 10.5 | 9.7 | 8.4 | 6.9 | 6.6 | 5.6% | |  | | 14.7 | 17 | 19.6 | 21.5 | 21.9 | | 10.1% | |  |
| TYD04* | Plan/Organise | I took steps to organise what I did each day | 2.01 |  | 13.5 | 12.4 | 10.7 | 8.8 | 8.7 | 7.3% |  | 10.2 | 9.8 | 8.7 | 7.7 | 7.8 | 2.6% | |  | | 15 | 16.5 | 18.6 | 20.7 | 21.5 | | 9.7% | |  |
|  |  |  |  |  |  |  |  |  |  |  |  |  |  |  |  |  |  | |  | |  |  |  |  |  | |  | |  |
| TYD42 | Problem Solving | I broke a large problem into smaller, more manageable steps | 1.41 |  | 12.5 | 11.2 | 9.9 | 8.6 | 8.1 | 5.2% |  | 9.8 | 9.4 | 8.2 | 7.1 | 6.8 | 3.5% | |  | | 15.7 | 18.1 | 19.6 | 21.9 | 22 | | 8.4% | |  |
| TYD11 | Problem Solving | I took steps to solve a problem that was affecting me | 1.71 |  | 12.6 | 11.9 | 10.1 | 9.1 | 8.5 | 4.4% |  | 9.5 | 9.7 | 8.5 | 7.7 | 7.3 | 2.0% | |  | | 15.6 | 17.1 | 19.6 | 20.7 | 21.3 | | 6.5% | |  |
|  |  |  |  |  |  |  |  |  |  |  |  |  |  |  |  |  |  | |  | |  |  |  |  |  | |  | |  |
| TYD16* | Respect/Self | I treated myself with respect | 2.17 |  | 16.7 | 13.4 | 10.8 | 8.8 | 6.6 | 25.5% |  | 12.7 | 10.9 | 9.3 | 7.3 | 5.4 | 19.8% | |  | | 13.3 | 15.6 | 18.4 | 20.6 | 22.3 | | 17.1% | |  |
| TYD69* | Respect/Self | I praised myself when I did something well | 1.34 |  | 13.5 | 11.2 | 8.9 | 8 | 6.8 | 11.7% |  | 10.7 | 9.3 | 7.4 | 6.9 | 5.6 | 8.6% | |  | | 15.5 | 18 | 20.4 | 21.4 | 23.2 | | 11.1% | |  |
| TYD53 | Respect/Others | I treated others with respect | 3.55 |  | 15.2 | 14.3 | 12.1 | 10.7 | 10.5 | 1.4% |  | 12 | 11.2 | 9.9 | 9.1 | 8.5 | 1.3% | |  | | 13.2 | 14.1 | 16.2 | 18.5 | 18.9 | | 2.1% | |  |
| TYD90* | Respect/Reflection | I took time to reflect on myself and how I felt | 1.95 |  | 12.1 | 11.2 | 9.9 | 10 | 10.6 | 0.6% |  | 9.5 | 9.1 | 8.1 | 8.8 | 8.5 | 0.3% | |  | | 16.4 | 17.7 | 19.4 | 19.7 | 19.6 | | 2.3% | |  |
|  |  |  |  |  |  |  |  |  |  |  |  |  |  |  |  |  |  | |  | |  |  |  |  |  | |  | |  |
| TYD29* | Social/Positive People | I socialised with positive people | 1.68 |  | 14.4 | 11.8 | 9.7 | 8.8 | 7.4 | 9.4% |  | 11.3 | 9.5 | 8.1 | 7.5 | 6.2 | 6.6% | |  | | 14.1 | 16.9 | 19.7 | 21.4 | 23.4 | | 13.2% | |  |
| TYD62 | Social/Positive People | I aimed to spend time with positive people | 1.76 |  | 13.3 | 12.2 | 10.3 | 9 | 7.4 | 9.5% |  | 10.3 | 9.9 | 8.7 | 7.6 | 6.4 | 5.7% | |  | | 15.1 | 16.6 | 19.2 | 20.9 | 22.7 | | 12.3% | |  |
| TYD33* | Social/Talking | I had a meaningful conversation with someone | 1.88 |  | 14.4 | 12.1 | 9.8 | 9.1 | 8 | 8.4% |  | 11.1 | 9.6 | 8.2 | 8 | 6.9 | 4.5% | |  | | 13.6 | 16.7 | 19.3 | 21.3 | 22.1 | | 11.6% | |  |
| TYD93 | Social/Improve belonging | I did something to improve my sense of belonging | 1.13 |  | 12.7 | 11.3 | 8.8 | 7.6 | 6.5 | 8.8% |  | 10 | 9.2 | 7.5 | 6.8 | 5.4 | 5.8% | |  | | 16.2 | 18 | 20.9 | 22.6 | 22.9 | | 8.9% | |  |
| TYD31* | Social/Talking | I talked about my day with a friend or a family member | 2.11 |  | 13.7 | 12.2 | 10.2 | 9.2 | 8.8 | 6.7% |  | 10.6 | 9.8 | 8.3 | 7.6 | 7.7 | 3.5% | |  | | 13.9 | 16.4 | 19 | 20.3 | 21.7 | | 12.4% | |  |
| TYD87 | Social/Improve relationships | I did something to improve my relationships with people who are important to me | 1.8 |  | 13.4 | 12 | 10.3 | 8.9 | 8.1 | 6.7% |  | 10.5 | 9.6 | 8.6 | 7.6 | 6.9 | 4.0% | |  | | 15.1 | 16.7 | 19.1 | 21.6 | 22 | | 9.6% | |  |
| TYD58 | Social/Positive People | I arranged to see friends | 1.03 |  | 12.6 | 10.6 | 9 | 8.2 | 8.2 | 4.2% |  | 10.1 | 8.7 | 7.4 | 7.2 | 7.2 | 2.7% | |  | | 16.2 | 18.6 | 20.9 | 22 | 21.4 | | 5.4% | |  |
| TYD63 | Social/Talking | I talked with a friend or family member on the phone | 1.53 |  | 12.7 | 10.9 | 10 | 10 | 9.1 | 2.5% |  | 10.1 | 8.9 | 8.1 | 8.6 | 7.7 | 1.4% | |  | | 16.2 | 18 | 19.8 | 19.5 | 21.1 | | 3.9% | |  |
| TYD37* | Social/Help others | I did something to help others | 2.28 |  | 13.5 | 11.5 | 10.7 | 9.7 | 10 | 1.6% |  | 10 | 9.2 | 8.7 | 8.2 | 8.5 | 0.4% | |  | | 14.3 | 16.8 | 18.5 | 20 | 20.1 | | 4.5% | |  |
| TYD51* | Social/Praise others | I encouraged or praised someone | 2.55 |  | 13.9 | 11.5 | 10.7 | 10.5 | 10.2 | 1.0% |  | 10.6 | 9.2 | 8.7 | 8.7 | 8.4 | 0.5% | |  | | 13.6 | 16.4 | 18 | 19.2 | 20.2 | | 4.8% | |  |
| TYD79 | Social/Help others | I did something to improve the quality of other people's lives | 2.14 |  | 13.4 | 11.5 | 10.2 | 10 | 10.1 | 1.7% |  | 10.2 | 8.9 | 8.6 | 8.4 | 8.5 | 0.4% | |  | | 14.7 | 17.2 | 18.9 | 20.2 | 19.9 | | 4.1% | |  |
| TYD75 | Social/Social media | I sent a personal email, text message, or made a post on social media to someone | 2.66 |  | 13.3 | 12.2 | 10.8 | 10.3 | 10 | 2.1% |  | 10.4 | 9.7 | 8.7 | 8.4 | 8.3 | 1.1% | |  | | 15.8 | 16.6 | 18.3 | 18.4 | 19.8 | | 2.7% | |  |
| TYD56* | Social/Kindness others | I did something kind for someone else | 2.23 |  | 13.4 | 11.3 | 10.4 | 10.4 | 10.3 | 0.7% |  | 9.9 | 9 | 8.5 | 8.7 | 8.8 | 0.1% | |  | | 14.6 | 17.2 | 18.7 | 19.6 | 19.8 | | 2.7% | |  |
|  |  |  |  |  |  |  |  |  |  |  |  |  |  |  |  |  |  | |  | |  |  |  |  |  | |  | |  |
| TYD82* | Values/Spiritual | I did something to help me live my "ideal" life | 1.42 |  | 13.6 | 11.6 | 9.1 | 7.3 | 7 | 12.1% |  | 10.5 | 9.4 | 7.9 | 6.5 | 6.2 | 6.7% | |  | | 14.4 | 17.6 | 20.6 | 23.5 | 23.4 | | 17.6% | |  |
| TYD76* | Values/Spiritual | I acted in a way that is consistent with my personal values | 2.99 |  | 17.4 | 15.1 | 12 | 10.3 | 9.1 | 10.6% |  | 13.6 | 11.9 | 9.8 | 8.6 | 7.5 | 8.1% | |  | | 11.8 | 14.1 | 16.7 | 18.9 | 20.3 | | 9.6% | |  |
| TYD77* | Values/Spiritual | I acted with integrity and dignity | 3.17 |  | 16.6 | 14.9 | 12.1 | 10.8 | 9.6 | 6.6% |  | 12.9 | 11.8 | 10 | 8.9 | 7.8 | 5.3% | |  | | 11.2 | 14.1 | 17.3 | 18.3 | 19.8 | | 6.3% | |  |
| TYD86* | Values/Spiritual | I did something to improve or maintain my spiritual wellbeing | 1.13 |  | 12.1 | 11.1 | 9.4 | 8.8 | 7.7 | 5.1% |  | 9.6 | 9.2 | 8 | 7.4 | 6.5 | 3.1% | |  | | 16.9 | 18.4 | 19.7 | 21.2 | 22.1 | | 5.5% | |  |
|  | *Items also included in Study 2; non-flagged items were explored in Study 1 only; TYD – Things You Do; Colours indicate the relationship between frequency of responses per item with scores on each outcome measure, with dark blue indicating the frequency most observed with desirable scores and dark red indicating the frequently most observed with undesirable scores. Table cells with bolded outline denote the weekly frequency threshold beyond which there is no statistically significant improvement in scores; *R* denotes a correlation coefficient and % variance explained (*R^2^*) | | | | | | | | | | | | | | | | | | | | | | | | | | | | |
