# Supplementary material for: Measures of Daily Activities Associated With Mental Health (Things You Do Questionnaire): Development of a Preliminary Psychometric Study and Replication Study
Source: JMIR Form Res. 2022 Jul 5;6(7):e38837. doi: 10.2196/38837 (PMC9297144; doi:10.2196/38837)
Supplement: Multimedia Appendix 3 [file formative_v6i7e38837_app3.docx]

**Multimedia Appendix 3**

| **Table 1.** Exploratory factor analysis solution and assigned factor labels for items grouped with no item selection criteria (96 items), and item grouping under *R^2^*>5%/10%/15% outcomes association criteria | | | | | | | | | | | | |
| --- | --- | --- | --- | --- | --- | --- | --- | --- | --- | --- | --- | --- |
| Item order | Primary/Secondary Cluster | | Item | Item→PHQ9 *R^2^* | Item→GAD7 *R^2^* | Item→SWLS *R^2^* | EFA Solution \| No Item Selection Criteria (Factor label \| item loading) | EFA Solution \| Item *R^2^*>5%  (Factor label \| item loading) | EFA Solution \| Item *R^2^*>10% (Factor label \| item loading) | EFA Solution \| Item *R^2^*>15% (Factor label \| item loading) |  |  |
| TYD88* | Activity/Enjoyable | | I did something enjoyable | 18.8% | 14.6% | 17.5% | Meaningful Activity \| 0.605 | Meaningful Activity \| 0.61 | Meaningful Activity \| 0.728 | Composite \| 0.766 |  |  |
| TYD66* | Activity/Satisfying | | I did something that was very satisfying to me | 17.8% | 12.1% | 16.3% | Meaningful Activity \| 0.541 | Meaningful Activity \| 0.593 | Meaningful Activity \| 0.679 | Composite \| 0.785 |  |  |
| TYD44* | Activity/Laugh, fun | | I had a good laugh or did something that was fun | 14.3% | 11.7% | 14.4% | λ≥0.5 | Meaningful Activity \| 0.52 | Meaningful Activity \| 0.651 | -- |  |  |
| TYD35* | Activity/Meaning | | I spent time doing something I believed in | 12.5% | 7.4% | 13.5% | Meaningful Activity \| 0.516 | Meaningful Activity \| 0.533 | Meaningful Activity \| 0.535 | -- |  |  |
| TYD89* | Activity/Avoid stagnant | | I avoided being 'stagnant' | 13.0% | 7.6% | 10.0% | λ≥0.5 | λ≥0.5 | λ≥0.5 | -- |  |  |
| TYD39∆ | Activity/Meaning | | I spent time doing something I think is important | 11.1% | 5.5% | 13.2% | λ≥0.5 | λ≥0.5 | Meaningful Activity \| 0.545 | -- |  |  |
| TYD78* | Activity/Improve quality of life | | I did something to improve or maintain the quality of my life | 10.4% | 6.9% | 11.6% | Meaningful Activity \| 0.637 | Meaningful Activity \| 0.578 | Meaningful Activity \| 0.681 | -- |  |  |
| TYD72* | Activity/Achieve goal | | I did something to help me achieve my goals | 9.6% | 4.3% | 11.9% | Goals/Plans \| 0.729 | Goals/Plans \| 0.703 | Goals/Plans\| 0.758 | -- |  |  |
| TYD59∆ | Activity/Meaning | | I worked on an activity that was meaningful to me | 9.9% | 5.4% | 9.4% | Activity/Meaning \| 0.548 | Activity/Enjoyable \| 0.548 | -- | -- |  |  |
| TYD34* | Activity/Interesting | | I did a hobby or something that was of interest to me | 9.7% | 7.3% | 5.5% | Activity/Enjoyable \| 0.753 | Activity/Enjoyable \| 0.774 | -- | -- |  |  |
| TYD13∆ | Activity/Meaning | | I read, listened, or watched something I enjoyed | 7.8% | 6.9% | 6.3% | Activity/Enjoyable \| 0.513 | λ≥0.5 | -- | -- |  |  |
| TYD10∆ | Activity/Meaning | | I practiced a skill or did a hobby | 5.7% | 4.5% | 3.9% | Activity/Enjoyable \| 0.684 | Activity/Enjoyable \| 0.724 | -- | -- |  |  |
| TYD23* | Activity/Meaning | | I put effort and time into something I wanted to change | 4.4% | 2.2% | 5.2% | Activity/Meaning \| 0.606 | Activity/Meaning\| 0.614 | -- | -- |  |  |
| TYD71*† | Activity/Learn new | | I tried to learn something new | 1.8% | 1.2% | 2.6% | λ≥0.5 | -- | -- | -- |  |  |
|  |  | |  |  |  |  |  |  |  |  |  |  |
| TYD70* | Cognitive/Perspective | | I kept a realistic perspective on things | 18.1% | 21.1% | 12.2% | Realistic Thinking \| 0.686 | Realistic Thinking \| 0.68 | Realistic Thinking \| 0.743 | Composite \| 0.621 |  |  |
| TYD17* | Cognitive/Future | | Instead of worrying about the past, I focused on my preferred future | 13.8% | 12.9% | 14.2% | Realistic Thinking \| 0.513 | λ≥0.5 | Realistic Thinking \| 0.549 | -- |  |  |
| TYD32∆ | Cognitive/Perspective | | I tried to keep things in perspective | 12.1% | 11.6% | 11.4% | Realistic Thinking \| 0.627 | Realistic Thinking \| 0.616 | Realistic Thinking \| 0.685 | -- |  |  |
| TYD68* | Cognitive/Challenging | | I stopped myself from thinking unhelpful or unrealistic thoughts | 11.2% | 11.1% | 7.7% | Realistic Thinking \| 0.62 | Realistic Thinking \| 0.568 | Realistic Thinking \| 0.619 | -- |  |  |
| TYD38∆ | Cognitive/Problem solving | | Instead of thinking about my worries, I focused on doing something about them | 10.2% | 7.2% | 10.0% | Realistic Thinking \| 0.508 | Realistic Thinking \| 0.535 | Realistic Thinking \| 0.556 | -- |  |  |
| TYD61* | Cognitive/Perspective | | I allowed myself to be less than perfect | 7.7% | 11.0% | 4.1% | Realistic Thinking \| 0.637 | Realistic Thinking \| 0.623 | Realistic Thinking \| 0.674 | -- |  |  |
| TYD55 | Cognitive/Challenging | | I talked myself out of negative thinking | 5.9% | 4.0% | 5.5% | Cognitive/Self-perception \| 0.508 | Cognitive/Challenging \| 0.513 | -- | -- |  |  |
| TYD27 | Cognitive/Challenging | | I identified unhelpful thoughts and tried to replace them with more helpful ones | 5.5% | 3.8% | 5.6% | λ≥0.5 | Cognitive/Challenging \| 0.56 | -- | -- |  |  |
|  |  | |  |  |  |  |  |  |  |  |  |  |
| TYD22* | Emotion Regulation/Coping | | I dealt with feelings of frustration or impatience in a healthy way | 12.6% | 10.7% | 9.8% | λ≥0.5 | λ≥0.5 | Realistic Thinking \| 0.528 | -- |  |  |
| TYD12∆ | Emotion Regulation/No excuses | | I didn't make excuses | 8.7% | 7.2% | 5.6% | λ≥0.5 | λ≥0.5 | -- | -- |  |  |
| TYD92* | Emotion Regulation/Expression | | I expressed my feelings honestly, instead of suppressing them | 5.7% | 4.7% | 8.0% | λ≥0.5 | λ≥0.5 | -- | -- |  |  |
| TYD20∆ | Emotion Regulation/Avoid chaos | | I avoided chaos in my life | 5.5% | 6.8% | 3.7% | λ≥0.5 | Emotion Regulation \| 0.574 | -- | -- |  |  |
| TYD95∆ | Emotion Regulation/Expression | | I was able to say no when I did not want to do something | 5.0% | 5.2% | 4.6% | λ≥0.5 | Emotion Regulation \| 0.529 | -- | -- |  |  |
| TYD25* | Emotion Regulation/Coping | | I dealt with things that were creating stress | 4.9% | 2.3% | 6.7% | λ≥0.5 | λ≥0.5 | -- | -- |  |  |
| TYD41 | Emotion Regulation/Patience | | I practiced being patient | 2.9% | 3.3% | 3.5% | λ≥0.5 | -- | -- | -- |  |  |
| TYD43*† | Emotion Regulation/Pushing through | | I made myself do something because I knew it would be beneficial | 3.6% | 1.6% | 4.6% | λ≥0.5 | -- | -- | -- |  |  |
| TYD52 | Emotion Regulation/Realistic promises | | I didn't promise or commit to doing things I couldn't do | 2.0% | 2.3% | 1.4% | λ≥0.5 | -- | -- | -- |  |  |
| TYD28*† | Emotion Regulation/Pushing through | | I faced a situation that was unpleasant but necessary | 0.4% | 1.6% | 0.0% | Emotion Regulation \| 0.63 | -- | -- | -- |  |  |
| TYD60*† | Emotion Regulation/Pushing through | | I pushed myself to do things that were difficult or triggered some stress | 0.0% | 1.1% | 0.7% | Emotion Regulation \| 0.734 | -- | -- | -- |  |  |
| TYD57 | Emotion Regulation/Coping | | I fulfilled my responsibilities even though I didn't want to | 0.3% | 0.0% | 1.3% | Emotion Regulation \| 0.566 | -- | -- | -- |  |  |
| TYD15 | Emotion Regulation/Pushing through | | I pushed myself to do things that I didn’t feel like doing | 0.1% | 0.2% | 0.8% | Emotion Regulation \| 0.653 | -- | -- | -- |  |  |
|  |  | |  |  |  |  |  |  |  |  |  |  |
| TYD80*† | Environment | | I did something to improve the quality of the physical environment | 3.9% | 1.8% | 4.8% | λ≥0.5 | -- | -- | -- |  |  |
|  |  | |  |  |  |  |  |  |  |  |  |  |
| TYD65∆ | Gratitude/Acceptance | | I tried to accept things that I couldn’t control or change | 7.9% | 9.2% | 7.6% | Cognitive/Self-perception \| 0.712 | Cognitive/Self-perception \| 0.714 | -- | -- |  |  |
| TYD08* | Gratitude/Acceptance | | I accepted a situation for what it is | 7.4% | 9.6% | 7.3% | Cognitive/Self-perception \| 0.635 | Cognitive/Self-perception \| 0.667 | -- | -- |  |  |
| TYD01* | Gratitude/Acceptance | | I thought about things that I am grateful for | 6.8% | 4.2% | 12.3% | λ≥0.5 | λ≥0.5 | -- | -- |  |  |
| TYD94*† | Gratitude/Acceptance | | I accepted my symptoms by allowing them to peak and pass | 1.2% | 1.0% | 1.6% | λ≥0.5 | -- | -- | -- |  |  |
|  |  | |  |  |  |  |  |  |  |  |  |  |
| TYD48* | Healthy Routine/Mental wellbeing | | I did things which are good for my mental wellbeing | 14.1% | 10.8% | 12.1% | λ≥0.5 | λ≥0.5 | λ≥0.5 | -- |  |  |
| TYD02* | Healthy Routine/General | | I kept a healthy daily routine | 15.8% | 8.2% | 11.8% | λ≥0.5 | λ≥0.5 | Healthy Routine \| 0.729 | Composite \| 0.598 |  |  |
| TYD81∆ | Healthy Routine/General satisfaction | | I did something to improve my satisfaction with my life | 10.4% | 6.0% | 12.0% | Meaningful Activity \| 0.671 | Meaningful Activity \| 0.592 | Meaningful Activity \| 0.696 | -- |  |  |
| TYD05* | Healthy Routine/Sleep | | I went to bed and woke up at a regular time | 12.5% | 6.6% | 7.4% | Healthy Routine/Sleep \| 0.559 | Healthy Routine/Chores \| 0.557 | Healthy Routine \| 0.727 | -- |  |  |
| TYD64* | Healthy Routine/Nutrition | | I prepared and ate a healthy meal | 10.8% | 5.7% | 6.4% | λ≥0.5 | λ≥0.5 | Healthy Routine \| 0.671 | -- |  |  |
| TYD91* | Healthy Routine/Physical health | | I did something to improve or maintain my physical health | 8.2% | 5.1% | 5.3% | Healthy Routine/exercise \| 0.787 | Healthy Routine/Physical health \| 0.789 | -- | -- |  |  |
| TYD30* | Healthy Routine/Outside | | I spent time outside | 7.3% | 5.2% | 4.2% | Healthy Routine/Outside \| 0.69 | Healthy Routine/Outside \| 0.749 | -- | -- |  |  |
| TYD85* | Healthy Routine/Sunlight | | I got regular exposure to sunlight (e.g., 15-30 mins) | 6.9% | 4.8% | 4.7% | Healthy Routine/Outside \| 0.73 | Healthy Routine/Outside \| 0.74 | -- | -- |  |  |
| TYD74* | Healthy Routine/Exercise | | I did some form of exercise (e.g. swimming, went for a walk, etc) | 6.5% | 3.8% | 4.2% | Healthy Routine/exercise \| 0.836 | Healthy Routine/Physical health \| 0.836 | -- | -- |  |  |
| TYD07∆ | Healthy Routine/Exercise | | I planned or stuck to an exercise routine | 6.4% | 3.5% | 4.2% | Healthy Routine/exercise \| 0.845 | Healthy Routine/Physical health \| 0.853 | -- | -- |  |  |
| TYD18* | Healthy Routine/Electronics | | I kept my use of electronic devices or games to a healthy level | 5.4% | 3.8% | 4.1% | Healthy Routine/social media \| 0.821 | λ≥0.5 | -- | -- |  |  |
| TYD67* | Healthy Routine/Hygiene | | I had a bath or shower | 6.1% | 2.6% | 4.0% | λ≥0.5 | λ≥0.5 | -- | -- |  |  |
| TYD50∆ | Healthy Routine/Exercise | | I did 30 minutes of exercise | 5.6% | 3.2% | 3.8% | Healthy Routine/exercise \| 0.862 | Healthy Routine/Physical health \| 0.859 | -- | -- |  |  |
| TYD73* | Healthy Routine/Chores | | I did work or chores around where I live (e.g., house, apartment, etc) | 4.9% | 2.1% | 5.3% | Healthy Routine/Chores \| 0.704 | Healthy Routine/Chores \| 0.588 | -- | -- |  |  |
| TYD24* | Healthy Routine/Organised | | I kept my home, living space, or workspace clean and organised | 4.9% | 2.1% | 5.2% | Healthy Routine/Chores \| 0.662 | Healthy Routine/Chores \| 0.668 | -- | -- |  |  |
| TYD83∆ | Healthy Routine/Finances | | I did something to improve or maintain my financial health | 4.0% | 1.9% | 5.6% | λ≥0.5 | Activity/Meaning\| 0.561 | -- | -- |  |  |
| TYD36 | Healthy Routine/Social | | I kept my use of social media and entertainment to a healthy level | 4.4% | 3.4% | 3.4% | Healthy Routine/social media \| 0.817 | -- | -- | -- |  |  |
| TYD26 | Healthy Routine/Hydration | | I made sure I drank a healthy amount of water | 4.2% | 2.9% | 2.9% | λ≥0.5 | -- | -- | -- |  |  |
| TYD21*† | Healthy Routine/Sleep | | I kept a relaxing bedtime routine, that did not involve watching videos or checking social media | 4.0% | 3.2% | 2.6% | λ≥0.5 | -- | -- | -- |  |  |
| TYD49*† | Healthy Routine/Relax | | I did something to help me relax (e.g., slow breathing, stretching etc) | 3.0% | 1.6% | 3.9% | λ≥0.5 | -- | -- | -- |  |  |
| TYD46 | Healthy Routine/Electronics | | I spent some time without the phone, TV, or internet on | 2.9% | 2.2% | 2.8% | λ≥0.5 | -- | -- | -- |  |  |
| TYD84 | Healthy Routine/Nutrition | | I avoided foods that caused emotional or physical problems, such as anxiety or indigestion | 2.6% | 1.8% | 1.0% | λ≥0.5 | -- | -- | -- |  |  |
| TYD06*† | Healthy Routine/Excesses | | I avoided unhealthy habits (e.g., I chose not to have a drink, or gamble, etc) | 2.5% | 1.5% | 1.6% | Healthy Routine Substance \| 0.781 | -- | -- | -- |  |  |
| TYD19 | Healthy Routine/Substance | | I avoided illicit drugs, and did not misuse medications | 2.6% | 1.6% | 1.1% | λ≥0.5 | -- | -- | -- |  |  |
| TYD09 | Healthy Routine/Finances | | I created and stuck to my budget | 2.0% | 1.0% | 1.9% | Finances \| 0.547 | -- | -- | -- |  |  |
| TYD03*† | Healthy Routine/Substance | | I had an alcohol free day | 0.6% | 0.5% | 0.3% | Healthy Routine Substance \| 0.814 | -- | -- | -- |  |  |
| TYD96*† | Healthy Routine/Silence, solitude | | I spent time in Silence/ solitude | 0.3% | 0.0% | 0.2% | Reflection/solitude \| 0.598 | -- | -- | -- |  |  |
|  |  | |  |  |  |  |  |  |  |  |  |  |
| TYD54* | Plan/Future | | I had something to look forward to | 22.5% | 14.4% | 25.9% | λ≥0.5 | Goals/Plans \| 0.516 | Goals/Plans \| 0.595 | Composite \| 0.787 |  |  |
| TYD14∆ | Plan/Future | | I gave myself things to look forward to | 17.4% | 10.8% | 18.7% | λ≥0.5 | λ≥0.5 | Goals/Plans \| 0.592 | Composite \| 0.802 |  |  |
| TYD40* | Plan/Realistic goals | | I set realistic and achievable goals | 11.6% | 7.5% | 13.5% | λ≥0.5 | λ≥0.5 | λ≥0.5 | -- |  |  |
| TYD45* | Plan/Personal responsibility | | I took responsibility for the direction of my life | 9.4% | 7.2% | 11.8% | λ≥0.5 | λ≥0.5 | λ≥0.5 | -- |  |  |
| TYD47* | Plan/Execute | | I made a plan and stuck to it | 10.0% | 5.6% | 10.1% | λ≥0.5 | Goals/Plans \| 0.545 | Goals/Plans \| 0.543 | -- |  |  |
| TYD04* | Plan/Organise | | I took steps to organise what I did each day | 7.3% | 2.6% | 9.7% | λ≥0.5 | λ≥0.5 | -- | -- |  |  |
|  |  | |  |  |  |  |  |  |  |  |  |  |
| TYD42∆ | Problem Solving | | I broke a large problem into smaller, more manageable steps | 5.2% | 3.5% | 8.4% | λ≥0.5 | Activity/Meaning \| 0.549 | -- | -- |  |  |
| TYD11∆ | Problem Solving | | I took steps to solve a problem that was affecting me | 4.4% | 2.0% | 6.5% | λ≥0.5 | Activity/Meaning \| 0.597 | -- | -- |  |  |
|  |  | |  |  |  |  |  |  |  |  |  |  |
| TYD16* | Respect/Self | | I treated myself with respect | 25.5% | 19.8% | 17.1% | Realistic Thinking \| 0.518 | λ≥0.5 | Realistic Thinking \| 0.594 | Composite \| 0.699 |  |  |
| TYD69* | Respect/Self | | I praised myself when I did something well | 11.7% | 8.6% | 11.1% | λ≥0.5 | λ≥0.5 | λ≥0.5 | -- |  |  |
| TYD53 | Respect/Others | | I treated others with respect | 1.4% | 1.3% | 2.1% | Spiritual \| 0.667 | -- | -- | -- |  |  |
| TYD90*† | Respect/Reflection | | I took time to reflect on myself and how I felt | 0.6% | 0.3% | 2.3% | Reflection/solitude \| 0.629 | -- | -- | -- |  |  |
|  |  | |  |  |  |  |  |  |  |  |  |  |
| TYD29* | Social/Positive People | | I socialised with positive people | 9.4% | 6.6% | 13.2% | Social Connections \| 0.547 | Social Connections \| 0.667 | Social Connections \| 0.589 | -- |  |  |
| TYD62∆ | Social/Positive People | | I aimed to spend time with positive people | 9.5% | 5.7% | 12.3% | Social Connections \| 0.51 | Social Connections \| 0.611 | Social Connections \| 0.532 | -- |  |  |
| TYD33* | Social/Talking | | I had a meaningful conversation with someone | 8.4% | 4.5% | 11.6% | Social Connections \| 0.605 | Social Connections \| 0.555 | Social Connections \| 0.726 | -- |  |  |
| TYD93∆ | Social/Improve belonging | | I did something to improve my sense of belonging | 8.8% | 5.8% | 8.9% | λ≥0.5 | λ≥0.5 | -- | -- |  |  |
| TYD31* | Social/Talking | | I talked about my day with a friend or a family member | 6.7% | 3.5% | 12.4% | Social Connections \| 0.681 | Social Connections \| 0.646 | Social Connections \| 0.764 | -- |  |  |
| TYD87∆ | Social/Improve relationships | | I did something to improve my relationships with people who are important to me | 6.7% | 4.0% | 9.6% | λ≥0.5 | λ≥0.5 | -- | -- |  |  |
| TYD58∆ | Social/Positive People | | I arranged to see friends | 4.2% | 2.7% | 5.4% | Social/Positive People \| 0.568 | Activity/Laugh/fun \| 0.656 | -- | -- |  |  |
| TYD63 | Social/Talking | | I talked with a friend or family member on the phone | 2.5% | 1.4% | 3.9% | Social/Talking \| 0.572 | -- | -- | -- |  |  |
| TYD37*† | Social/Help others | | I did something to help others | 1.6% | 0.4% | 4.5% | Social/Help others \| 0.777 | -- | -- | -- |  |  |
| TYD51*† | Social/Praise others | | I encouraged or praised someone | 1.0% | 0.5% | 4.8% | Social/Help others \| 0.621 | -- | -- | -- |  |  |
| TYD79 | Social/Help others | | I did something to improve the quality of other people's lives | 1.7% | 0.4% | 4.1% | Social/Help others \| 0.751 | -- | -- | -- |  |  |
| TYD75 | Social/Social media | | I sent a personal email, text message, or made a post on social media to someone | 2.1% | 1.1% | 2.7% | Social/Talking \| 0.509 | -- | -- | -- |  |  |
| TYD56* | Social/Kindness others | | I did something kind for someone else | 0.7% | 0.1% | 2.7% | Social/Help others \| 0.751 | -- | -- | -- |  |  |
|  |  | |  |  |  |  |  |  |  |  |  |  |
| TYD82* | Values/Spiritual | | I did something to help me live my "ideal" life | 12.1% | 6.7% | 17.6% | Meaningful Activity \| 0.679 | Meaningful Activity \| 0.609 | Meaningful Activity \| 0.705 | Composite \| 0.7 |  |  |
| TYD76* | Values/Spiritual | | I acted in a way that is consistent with my personal values | 10.6% | 8.1% | 9.6% | Spiritual \| 0.615 | Spiritual \| 0.695 | λ≥0.5 | -- |  |  |
| TYD77* | Values/Spiritual | | I acted with integrity and dignity | 6.6% | 5.3% | 6.3% | Spiritual \| 0.698 | Spiritual \| 0.718 | -- | -- |  |  |
| TYD86* | Values/Spiritual | | I did something to improve or maintain my spiritual wellbeing | 5.1% | 3.1% | 5.5% | λ≥0.5 | Cognitive/Challenging \| 0.586 | -- | -- |  |  |
|  |  | |  |  |  |  |  |  |  |  |  |  |
| **Number of factors extracted** | | |  | **--** |  |  | **16 (56 items)** | **11 (45 items)** | **5 (27 items)** | **1 Factor (8 items)** |  |  |
| *Items also included in Study 2; † Items included in Study 2 on the merits of clinical consensus; ∆ Items removed in Study 2 on the merits of clinical consensus; Non-flagged items were explored in Study 1 only; TYD – Things You Do; λ denotes factor loading with weak loading items (<.5) suppressed from the Table and consequent CFA analyses; EFA – exploratory factor analysis; *R* denotes a correlation coefficient and % variance explained (*R^2^*); Colours indicate the magnitude of relationship with the outcome measures, with dark blue indicating the least observed relationship and dark red indicating the strongest observed relationship. | | | | | | | | | | | | |
|  | | |  | | | | | | | | | |
|  | | |  | | | | | | | | | |
