# Supplementary material for: Measures of Daily Activities Associated With Mental Health (Things You Do Questionnaire): Development of a Preliminary Psychometric Study and Replication Study
Source: JMIR Form Res. 2022 Jul 5;6(7):e38837. doi: 10.2196/38837 (PMC9297144; doi:10.2196/38837)
Supplement: Multimedia Appendix 4 [file formative_v6i7e38837_app4.docx]

**Multimedia Appendix 4.**

| **Table 1.** A heat map of the Identified factor association to PHQ-9, GAD-7 and SWLS outcomes, and the behavioural frequency thresholds associated with optimal mental health gains. | | | | | | | | | | | | | | | | | | | | | | | | | | |
| --- | --- | --- | --- | --- | --- | --- | --- | --- | --- | --- | --- | --- | --- | --- | --- | --- | --- | --- | --- | --- | --- | --- | --- | --- | --- | --- |
|  | | **Not at all** | **1-2 day \| Week** | **Half the week** | **Most** | **Daily** |  |  |  | **Not at all** | **1-2 day \| Week** | **Half the week** | **Most** | **Daily** |  |  |  | **Not at all** | **1-2 day \| Week** | **Half the week** | **Most** | **Daily** |  |  | |  |
|  | | **(0)** | **(1)** | **(2)** | **(3)** | **(4)** |  |  |  | **(0)** | **(1)** | **(2)** | **(3)** | **(4)** |  |  |  | **(0)** | **(1)** | **(2)** | **(3)** | **(4)** |  |  | |  |
| **Factor (number of items)** | | **Item → PHQ-9 mean scores** | | |  |  | ***R  bivariate*** | ***R  part*** | ***p-value  part*** | **Item → GAD-7 mean scores** | | | | | ***R  bivariate*** | ***R  part*** | ***p-value  part*** | **Item → SWLS mean scores** | | | |  | ***R  bivariate*** | ***R  part*** | | ***p-value  part*** |
| Activity/Meaning (8 items) | | 15.4 | 12.5 | 9.6 | 7.3 | 5.4 | -0.42 | -0.06 | <0.001 | 11.4 | 10 | 8.1 | 6.2 | 5.4 | -0.32 | 0.00 | 0.846 | 12.6 | 16.5 | 20 | 23.3 | 23.9 | 0.44 | 0.11 | | <0.001 |
| Cognitive (9 items) | | 16.9 | 14 | 11.4 | 8.1 | 6 | -0.48 | -0.22 | <0.001 | 13.4 | 11.8 | 9.1 | 6.7 | 4.6 | -0.49 | -0.31 | <0.001 | 12.4 | 15.1 | 17.7 | 21.5 | 23.2 | 0.43 | 0.13 | | <0.001 |
| Health Routine/exercise (4 items) | | 13.4 | 11.4 | 9.7 | 8.6 | 8.1 | -0.29 | -0.06 | <0.001 | 10.4 | 9.3 | 8.2 | 7.3 | 7 | -0.23 | -0.04 | 0.009 | 15.9 | 18.1 | 19.6 | 20.1 | 21.1 | 0.23 | 0.03 | | 0.041 |
| Activity/Enjoyable (5 items) | | 16.7 | 13.4 | 10 | 7.9 | 6.4 | -0.44 | -0.11 | <0.001 | 12.9 | 10.8 | 8.4 | 6.6 | 5 | -0.39 | -0.11 | <0.001 | 12.9 | 15.7 | 19.3 | 21 | 23.6 | 0.39 | 0.05 | | 0.001 |
| Social/Help others (4 items) | | 14.1 | 12 | 11 | 10.1 | 9.8 | -0.13 | 0.06 | <0.001 | 10.4 | 9.5 | 8.8 | 8.5 | 8.3 | -0.07 | 0.06 | <0.001 | 13.2 | 16 | 18 | 19.8 | 20.6 | 0.24 | 0.00 | | 0.822 |
| Social/Talking (4 items) | | 16.5 | 13.3 | 10.8 | 9 | 8.1 | -0.30 | -0.06 | <0.001 | 12.3 | 10.4 | 8.7 | 7.8 | 7 | -0.22 | -0.03 | 0.022 | 12.6 | 15 | 18.2 | 21 | 22.6 | 0.37 | 0.10 | | <0.001 |
| Emotion Regulation (4 items) | | 12.8 | 11.8 | 10 | 10.6 | 11.7 | -0.01 | 0.16 | <0.001 | 8.8 | 8.9 | 8 | 9.2 | 10.3 | 0.09 | 0.22 | <0.001 | 14.2 | 16.7 | 19.1 | 19.3 | 18.4 | 0.10 | -0.10 | | <0.001 |
| Reflection (2 items) | | 11.8 | 10.6 | 11.3 | 10.3 | 10.5 | -0.03 | 0.19 | <0.001 | 10 | 8.8 | 9.2 | 8.4 | 8.4 | -0.05 | 0.14 | <0.001 | 17.2 | 18 | 18 | 18.9 | 19.5 | 0.07 | -0.12 | | <0.001 |
| Social media (2 items) | | 13 | 11.7 | 10.2 | 10 | 8.3 | -0.24 | -0.01 | 0.308 | 10.3 | 9.7 | 8.5 | 8.3 | 6.9 | -0.20 | -0.02 | 0.268 | 16.3 | 17.6 | 18.8 | 19.7 | 20.8 | 0.20 | 0.00 | | 0.938 |
| Health Routine/domestic (2 items) | | 15.7 | 13.2 | 10.7 | 9.7 | 8.9 | -0.26 | -0.04 | 0.011 | 12.3 | 10 | 8.8 | 8.1 | 7.8 | -0.17 | 0.01 | 0.360 | 12.6 | 15.8 | 18.3 | 19.6 | 21 | 0.27 | 0.06 | | <0.001 |
| Social/Positive People (3 items) | | 14.2 | 11.8 | 9.5 | 7.4 | 6.7 | -0.35 | -0.03 | 0.065 | 11.1 | 9.6 | 7.9 | 6.4 | 6.3 | -0.28 | -0.01 | 0.460 | 13.9 | 17 | 20.1 | 23.3 | 23.7 | 0.40 | 0.08 | | <0.001 |
| Spiritual (3 items) | | 15.6 | 14.9 | 14 | 11.1 | 9.2 | -0.30 | -0.06 | <0.001 | 12.9 | 12 | 11.2 | 9.2 | 7.5 | -0.27 | -0.06 | <0.001 | 12 | 12.7 | 15.3 | 18 | 20.4 | 0.30 | 0.05 | | 0.001 |
| Health Routine/Sleep (1 item) | | 14.3 | 11.6 | 9.7 | 8.9 | 7.9 | -0.35 | -0.16 | <0.001 | 11 | 9.5 | 7.9 | 7.8 | 7 | -0.25 | -0.09 | <0.001 | 15.4 | 17.5 | 19.6 | 20.3 | 20.8 | 0.27 | 0.08 | | <0.001 |
| Health Routine/Substance (2 items) | | 12.3 | 11 | 11.2 | 10.3 | 10.5 | -0.05 | 0.02 | 0.114 | 9.9 | 8.8 | 9.1 | 8.4 | 8.7 | -0.04 | 0.02 | 0.164 | 16.6 | 18.1 | 18.3 | 19.1 | 18.6 | 0.04 | -0.04 | | 0.017 |
| Health Routine/Outside (2 items) | | 15.7 | 13.2 | 11.3 | 10.3 | 8.6 | -0.29 | 0.00 | 0.860 | 12.2 | 10.4 | 9.2 | 8.6 | 7.1 | -0.24 | -0.02 | 0.138 | 14.4 | 16.4 | 17.7 | 19.1 | 20.5 | 0.23 | -0.04 | | 0.005 |
| Finances (1 item) | | 11.9 | 11.6 | 10.4 | 9.5 | 9.8 | -0.14 | 0.01 | 0.008 | 9.5 | 9.4 | 8.8 | 8 | 8.1 | 0.14 | 0.00 | <0.001 | 17.1 | 17.9 | 19.3 | 19.6 | 19.6 | 0.14 | 0.00 | | <0.001 |
| **Model *R^2^* (16 factors – 56 items)** | |  |  |  |  |  |  | **40.7%** |  |  |  |  |  |  |  | **36.9%** |  |  |  |  |  |  |  | **31.8%** | |  |
|  | |  |  |  |  |  |  |  |  |  |  |  |  |  |  |  |  |  |  |  |  |  |  |  | |  |
| Activity Meaning (10 items) | | 15.5 | 12.2 | 9.8 | 6.9 | 6 | -0.39 | 0.06 | <0.001 | 11.5 | 9.9 | 8.1 | 6.2 | 5.7 | -0.30 | 0.10 | <0.001 | 12.3 | 16.6 | 20 | 23.5 | 23.7 | 0.43 | 0.04 | | 0.008 |
| Cognitive self rep (6 items) | | 18.1 | 14 | 12 | 8.8 | 6.7 | -0.43 | -0.14 | <0.001 | 15.1 | 11.8 | 9.9 | 7.1 | 5 | -0.47 | -0.26 | <0.001 | 11.4 | 14.9 | 17.4 | 20.7 | 22.4 | 0.38 | 0.07 | | <0.001 |
| Activity Enjoyable (6 items) | | 16.7 | 12.8 | 9.7 | 7.5 | 5.1 | -0.45 | -0.12 | <0.001 | 12.2 | 10.4 | 8.2 | 6.2 | 4.3 | -0.38 | -0.10 | <0.001 | 12.7 | 16.2 | 19.8 | 21.9 | 24.6 | 0.42 | 0.04 | | 0.010 |
| Activity Laugh fun (5 items) | | 16.3 | 12.5 | 9.6 | 6.2 | 5.1 | -0.46 | -0.13 | <0.001 | 12.5 | 10 | 8.1 | 5.3 | 4.7 | -0.38 | -0.10 | <0.001 | 12.5 | 15.9 | 20.3 | 24.4 | 25 | 0.50 | 0.18 | | <0.001 |
| Health Routine Physical (4 items) | | 13.4 | 11.4 | 9.5 | 8.4 | 7.7 | -0.29 | -0.07 | <0.001 | 10.3 | 9.3 | 8 | 7.2 | 6.6 | -0.23 | -0.05 | 0.001 | 15.9 | 18 | 20 | 20.3 | 21.3 | 0.23 | 0.03 | | 0.082 |
| Health Routine domestic (3 items) | | 16.9 | 13 | 10.5 | 9 | 7.5 | -0.36 | -0.13 | <0.001 | 12.7 | 10 | 8.5 | 7.8 | 6.9 | -0.25 | -0.04 | 0.005 | 12.8 | 15.9 | 18.8 | 20.7 | 21.7 | 0.33 | 0.10 | | <0.001 |
| Cognitive Challenging (2 items) | | 13.8 | 12.3 | 9.9 | 9.2 | 7.9 | -0.27 | 0.05 | 0.001 | 10.7 | 10 | 8.1 | 7.6 | 6.7 | -0.22 | 0.09 | <0.001 | 15.2 | 16.6 | 19.6 | 20.4 | 21.6 | 0.27 | -0.04 | | 0.004 |
| Social Talking (2 items) | | 15.4 | 13 | 10.8 | 9 | 8.1 | -0.32 | -0.03 | 0.097 | 11.7 | 10.3 | 8.6 | 7.7 | 7.1 | -0.23 | 0.00 | 0.859 | 12.5 | 15.4 | 18.2 | 20.8 | 22.7 | 0.39 | 0.09 | | <0.001 |
| Values Spiritual (2 items) | | 17.5 | 15.3 | 13.9 | 11 | 9.2 | -0.32 | -0.06 | <0.001 | 14 | 12.1 | 11.1 | 9.2 | 7.5 | -0.29 | -0.04 | 0.018 | 10.3 | 12.9 | 15.3 | 18.2 | 20.3 | 0.32 | 0.06 | | <0.001 |
| Health Routine Outside (2 items) | | 15.8 | 13.2 | 11.3 | 10.3 | 8.6 | -0.29 | -0.01 | 0.530 | 12.2 | 10.4 | 9.2 | 8.6 | 7.1 | -0.24 | -0.02 | 0.220 | 14.5 | 16.4 | 17.7 | 19.2 | 20.5 | 0.23 | -0.03 | | 0.035 |
| Emotion Regulation (2 items) | | 15.5 | 12.5 | 10.8 | 9.7 | 7.8 | -0.29 | -0.04 | 0.018 | 12.4 | 10.6 | 8.8 | 7.7 | 6 | -0.31 | -0.08 | <0.001 | 14.1 | 16.6 | 18.4 | 19.8 | 21.4 | 0.26 | 0.01 | | 0.401 |
| **Model *R^2^* (11 factors 45 items)** | |  |  |  |  |  |  | **32.6%** |  |  |  |  |  |  |  | **28.7%** |  |  |  |  |  |  |  | **31.6%** | |  |
|  | |  |  |  |  |  |  |  |  |  |  |  |  |  |  |  |  |  |  |  |  |  |  |  | |  |
| Goals and Plans (8 items) | | 16.2 | 12.8 | 10 | 7 | 5.9 | -0.44 | 0.07 | <0.001 | 12 | 10.2 | 8.5 | 6 | 5.6 | -0.33 | 0.12 | <0.001 | 11.7 | 16 | 19.5 | 23.2 | 24.2 | 0.47 | 0.03 | | 0.087 |
| Realistic Thinking (7 items) | | 18.1 | 14.2 | 11.1 | 7.8 | 4.5 | -0.52 | -0.19 | <0.001 | 14.6 | 11.9 | 9 | 6.2 | 3.4 | -0.52 | -0.30 | <0.001 | 11.9 | 14.9 | 18.2 | 21.7 | 24.4 | 0.45 | 0.08 | | <0.001 |
| Meaningful Activities (5 items) | | 17.7 | 13.1 | 9.7 | 7.2 | 4.3 | -0.53 | -0.21 | <0.001 | 13.4 | 10.5 | 8.3 | 5.9 | 3.9 | -0.45 | -0.19 | <0.001 | 11.7 | 15.6 | 19.5 | 22.9 | 26.1 | 0.53 | 0.18 | | <0.001 |
| Social Connections (4 items) | | 16 | 13.1 | 10.5 | 8.4 | 6.5 | -0.38 | 0.00 | 0.914 | 12 | 10.3 | 8.7 | 7.1 | 6 | -0.29 | 0.03 | 0.035 | 12 | 15.2 | 18.8 | 22 | 24.5 | 0.46 | 0.11 | | <0.001 |
| Healthy Routines (3 items) | | 16.9 | 13 | 10.3 | 7.8 | 5.8 | -0.46 | -0.21 | <0.001 | 12.5 | 10.2 | 8.5 | 7 | 5.6 | -0.33 | -0.10 | <0.001 | 13.3 | 16.3 | 19.1 | 21.1 | 22.9 | 0.37 | 0.09 | | <0.001 |
| **Model *R^2^* (5 factors – 27 items)** | |  |  |  |  |  |  | **38.1%** |  |  |  |  |  |  |  | **31.4** |  |  |  |  |  |  |  | **33.2%** | |  |
|  | |  |  |  |  |  |  |  |  |  |  |  |  |  |  |  |  |  |  |  |  |  |  |  | |  |
| Composite factor | | 18.9 | 14.2 | 10.2 | 6 | 3.6 | -- | -- | -- | 14.2 | 11.4 | 8.4 | 5.3 | 3.2 | -- | -- | -- | 10.5 | 14.5 | 19.2 | 23.5 | 27.1 | -- | -- | | -- |
| **Model *R^2^* (1 factor – 8 items)** | |  |  |  |  |  |  | **36.6%** |  |  |  |  |  |  |  | **26.7%** |  |  |  |  |  |  |  | **32.9%** | |  |
|  | |  |  |  |  |  |  |  |  |  |  |  |  |  |  |  |  |  |  |  |  |  |  |  | |  |
| Binary probability estimates | | Item → probability PHQ-9≥10 | | | | | AUC | OR | p-value | Item → probability GAD-7≥10 | | | | | AUC | OR | p-value | Item → probability SWLS≥10 | | | | | AUC | OR | | p-value |
| Activity/Meaning (8 items) | | 82% | 67% | 48% | 26% | 22% | 70.4% | 1.25 | 0.002 | 66% | 52% | 39% | 24% | 22% | 64.7% | 1.49 | <0.001 | 25% | 53% | 73% | 84% | 89% | 70.4% | 1.12 | | 0.137 |
| Cognitive (7 items) | | 88% | 77% | 55% | 33% | 11% | 74.1% | 0.52 | <0.001 | 82% | 66% | 42% | 23% | 9% | 73.2% | 0.39 | <0.001 | 28% | 46% | 66% | 79% | 89% | 74.1% | 1.22 | | 0.001 |
| Activity Enjoyable (5 items) | | 88% | 69% | 46% | 28% | 12% | 74.1% | 0.51 | <0.001 | 75% | 53% | 38% | 25% | 11% | 69.0% | 0.61 | <0.001 | 25% | 50% | 74% | 86% | 93% | 74.1% | 1.82 | | <0.001 |
| Social (4 items) | | 80% | 66% | 53% | 36% | 26% | 66.9% | 1.01 | 0.890 | 63% | 53% | 42% | 31% | 26% | 62.1% | 1.11 | 0.057 | 26% | 48% | 69% | 81% | 90% | 66.9% | 1.42 | | <0.001 |
| Health Routine General (3 items) | | 87% | 69% | 49% | 32% | 22% | 71.5% | 0.59 | <0.001 | 68% | 54% | 39% | 30% | 20% | 65.5% | 0.76 | <0.001 | 35% | 54% | 69% | 77% | 83% | 71.5% | 1.22 | | <0.001 |
|  | |  |  |  |  |  |  |  |  |  |  |  |  |  |  |  |  |  |  |  |  |  |  |  | |  |
| **Composite factor** | | **91%** | **77%** | **49%** | **20%** | **9%** | **77.5%** | **0.28** | **<0.001** | **81%** | **61%** | **38%** | **19%** | **7%** | **73.5%** | **0.38** | **<0.001** | **18%** | **44%** | **72%** | **87%** | **97%** | **75.2%** | **3.15** | | **<0.001** |
| Table cells with bolded outline denote the weekly TYD frequency threshold associated with incremental improved outcome scores (ie., the TYD frequency threshold beyond which there is no statistically significant improvement in scores); *R* denotes a correlation coefficient and % variance explained (*R^2^*); OR – odds ratio; AUC – area under the curve; Colours indicate the magnitude of relationship with the outcome measures, with dark blue indicating the least observed relationship and dark red indicating the strongest observed relationship. | | | | | | | | | | | | | | | | | | | | | | | | | |  |
