# Supplementary material for: Measures of Daily Activities Associated With Mental Health (Things You Do Questionnaire): Development of a Preliminary Psychometric Study and Replication Study
Source: JMIR Form Res. 2022 Jul 5;6(7):e38837. doi: 10.2196/38837 (PMC9297144; doi:10.2196/38837)
Supplement: Multimedia Appendix 5 [file formative_v6i7e38837_app5.docx]

| **Multimedia Appendix 5**  **Table 1.** Exploratory Factor analysis diagnostic statistics – item commonalities, factor eigenvalues, factor Cronbach’s alpha and item mean intercorrelations. | | | | | | | | | | | | |
| --- | --- | --- | --- | --- | --- | --- | --- | --- | --- | --- | --- | --- |
|  |  |  |  |  |  |  |  | Factor assigned label and item→factor loading | Item reproducibility by factor | Factor reliability analysis |  |  |
| Item order | Primary/Secondary Cluster | | Item wording | Item→PHQ9 *R^2^* | Item→GAD7 *R^2^* | Item→SWLS *R^2^* |  | (Factor label \| item loading) | Commonalities \| No Item Selection Criteria | Cronbach's Alpha if Item Deleted | Cronbach's Alpha | Mean Inter-Item Correlations |
| TYD59∆ | Activity/Meaning | | I worked on an activity that was meaningful to me | 9.90% | 5.40% | 9.40% |  | Activity/Meaning \| 0.548 | 56.54% | 0.707 | 0.759 | 0.383 |
| TYD34* | Activity/Interesting | | I did a hobby or something that was of interest to me | 9.70% | 7.30% | 5.50% |  | Activity/Enjoyable \| 0.753 | 70.75% | 0.669 |  |  |
| TYD13∆ | Activity/Meaning | | I read, listened, or watched something I enjoyed | 7.80% | 6.90% | 6.30% |  | Activity/Enjoyable \| 0.513 | 50.26% | 0.764 |  |  |
| TYD10∆ | Activity/Meaning | | I practiced a skill or did a hobby | 5.70% | 4.50% | 3.90% |  | Activity/Enjoyable \| 0.684 | 63.12% | 0.680 |  |  |
| TYD23* | Activity/Meaning | | I put effort and time into something I wanted to change | 4.40% | 2.20% | 5.20% |  | Activity/Meaning \| 0.606 | 54.90% | 0.746 |  |  |
|  |  |  |  |  |  |  |  |  |  |  |  |  |
| TYD65∆ | Gratitude/Acceptance | | I tried to accept things that I couldn’t control or change | 7.90% | 9.20% | 7.60% |  | Cognitive/Self-perception \| 0.712 | 59.67% | 0.502 | 0.697 | 0.433 |
| TYD08* | Gratitude/Acceptance | | I accepted a situation for what it is | 7.40% | 9.60% | 7.30% |  | Cognitive/Self-perception \| 0.635 | 50.70% | 0.582 |  |  |
| TYD55 | Cognitive/Challenging | | I talked myself out of negative thinking | 5.90% | 4.00% | 5.50% |  | Cognitive/Self-perception \| 0.508 | 54.95% | 0.710 |  |  |
|  |  |  |  |  |  |  |  |  |  |  |  |  |
| TYD28*† | Emotion Regulation/Pushing through | | I faced a situation that was unpleasant but necessary | 0.40% | 1.60% | 0.00% |  | Emotion Regulation \| 0.63 | 50.74% | 0.689 | 0.713 | 0.382 |
| TYD60*† | Emotion Regulation/Pushing through | | I pushed myself to do things that were difficult or triggered some stress | 0.00% | 1.10% | 0.70% |  | Emotion Regulation \| 0.734 | 62.51% | 0.591 |  |  |
| TYD57 | Emotion Regulation/Coping | | I fulfilled my responsibilities even though I didn't want to | 0.30% | 0.00% | 1.30% |  | Emotion Regulation \| 0.566 | 53.26% | 0.687 |  |  |
| TYD15 | Emotion Regulation/Pushing through | | I pushed myself to do things that I didn’t feel like doing | 0.10% | 0.20% | 0.80% |  | Emotion Regulation \| 0.653 | 54.56% | 0.626 |  |  |
|  |  |  |  |  |  |  |  |  |  |  |  |  |
| TYD09 | Healthy Routine/Finances | | I created and stuck to my budget | 2.00% | 1.00% | 1.90% |  | Finances \| 0.547 | 54.61% | -- | -- |  |
|  |  |  |  |  |  |  |  |  |  |  |  |  |
| TYD72* | Activity/Achieve goal | | I did something to help me achieve my goals | 9.60% | 4.30% | 11.90% |  | Goals/Plans \| 0.729 | 65.91% | -- | -- |  |
|  |  |  |  |  |  |  |  |  |  |  |  |  |
| TYD06*† | Healthy Routine/Excesses | | I avoided unhealthy habits (e.g., I chose not to have a drink, or gamble, etc) | 2.50% | 1.50% | 1.60% |  | Healthy Routine Substance \| 0.781 | 70.44% | -- | -- |  |
| TYD03*† | Healthy Routine/Substance | | I had an alcohol free day | 0.60% | 0.50% | 0.30% |  | Healthy Routine Substance \| 0.814 | 68.79% | -- |  |  |
|  |  |  |  |  |  |  |  |  |  |  |  |  |
| TYD24* | Healthy Routine/Organised | | I kept my home, living space, or workspace clean and organised | 4.90% | 2.10% | 5.20% |  | Healthy Routine/Chores \| 0.662 | 56.44% | -- | -- |  |
| TYD73* | Healthy Routine/Chores | | I did work or chores around where I live (e.g., house, apartment, etc) | 4.90% | 2.10% | 5.30% |  | Healthy Routine/Chores \| 0.704 | 65.32% | -- |  |  |
|  |  |  |  |  |  |  |  |  |  |  |  |  |
| TYD05* | Healthy Routine/Sleep | | I went to bed and woke up at a regular time | 12.50% | 6.60% | 7.40% |  | Healthy Routine/Sleep \| 0.559 | 47.26% | 0.882 | 0.856 | 0.466 |
| TYD91* | Healthy Routine/Physical health | | I did something to improve or maintain my physical health | 8.20% | 5.10% | 5.30% |  | Healthy Routine/exercise \| 0.787 | 76.01% | 0.821 |  |  |
| TYD30* | Healthy Routine/Outside | | I spent time outside | 7.30% | 5.20% | 4.20% |  | Healthy Routine/Outside \| 0.69 | 74.70% | 0.842 |  |  |
| TYD85* | Healthy Routine/Sunlight | | I got regular exposure to sunlight (e.g., 15-30 mins) | 6.90% | 4.80% | 4.70% |  | Healthy Routine/Outside \| 0.73 | 74.76% | 0.846 |  |  |
| TYD74* | Healthy Routine/Exercise | | I did some form of exercise (e.g. swimming, went for a walk, etc) | 6.50% | 3.80% | 4.20% |  | Healthy Routine/exercise \| 0.836 | 79.59% | 0.814 |  |  |
| TYD07∆ | Healthy Routine/Exercise | | I planned or stuck to an exercise routine | 6.40% | 3.50% | 4.20% |  | Healthy Routine/exercise \| 0.845 | 78.04% | 0.825 |  |  |
| TYD50∆ | Healthy Routine/Exercise | | I did 30 minutes of exercise | 5.60% | 3.20% | 3.80% |  | Healthy Routine/exercise \| 0.862 | 81.62% | 0.815 |  |  |
|  |  |  |  |  |  |  |  |  |  |  |  |  |
| TYD18* | Healthy Routine/Electronics | | I kept my use of electronic devices or games to a healthy level | 5.40% | 3.80% | 4.10% |  | Healthy Routine/social media \| 0.821 | 76.66% | -- | -- |  |
| TYD36 | Healthy Routine/Social | | I kept my use of social media and entertainment to a healthy level | 4.40% | 3.40% | 3.40% |  | Healthy Routine/social media \| 0.817 | 75.36% | -- |  |  |
|  |  |  |  |  |  |  |  |  |  |  |  |  |
| TYD88* | Activity/Enjoyable | | I did something enjoyable | 18.80% | 14.60% | 17.50% |  | Meaningful Activity \| 0.605 | 66.32% | 0.861 | 0.875 | 0.540 |
| TYD66* | Activity/Satisfying | | I did something that was very satisfying to me | 17.80% | 12.10% | 16.30% |  | Meaningful Activity \| 0.541 | 65.31% | 0.851 |  |  |
| TYD82* | Values/Spiritual | | I did something to help me live my "ideal" life | 12.10% | 6.70% | 17.60% |  | Meaningful Activity \| 0.679 | 62.62% | 0.851 |  |  |
| TYD35* | Activity/Meaning | | I spent time doing something I believed in | 12.50% | 7.40% | 13.50% |  | Meaningful Activity \| 0.516 | 63.87% | 0.865 |  |  |
| TYD78* | Activity/Improve quality of life | | I did something to improve or maintain the quality of my life | 10.40% | 6.90% | 11.60% |  | Meaningful Activity \| 0.637 | 64.47% | 0.846 |  |  |
| TYD81∆ | Healthy Routine/General satisfaction | | I did something to improve my satisfaction with my life | 10.40% | 6.00% | 12.00% |  | Meaningful Activity \| 0.671 | 67.79% | 0.844 |  |  |
|  |  |  |  |  |  |  |  |  |  |  |  |  |
| TYD16* | Respect/Self |  | I treated myself with respect | 25.50% | 19.80% | 17.10% |  | Realistic Thinking \| 0.518 | 56.36% | 0.824 | 0.848 | 0.447 |
| TYD70* | Cognitive/Perspective | | I kept a realistic perspective on things | 18.10% | 21.10% | 12.20% |  | Realistic Thinking \| 0.686 | 61.44% | 0.818 |  |  |
| TYD17* | Cognitive/Future | | Instead of worrying about the past, I focused on my preferred future | 13.80% | 12.90% | 14.20% |  | Realistic Thinking \| 0.513 | 49.13% | 0.827 |  |  |
| TYD32∆ | Cognitive/Perspective | | I tried to keep things in perspective | 12.10% | 11.60% | 11.40% |  | Realistic Thinking \| 0.627 | 59.45% | 0.819 |  |  |
| TYD68* | Cognitive/Challenging | | I stopped myself from thinking unhelpful or unrealistic thoughts | 11.20% | 11.10% | 7.70% |  | Realistic Thinking \| 0.62 | 58.71% | 0.829 |  |  |
| TYD38∆ | Cognitive/Problem solving | | Instead of thinking about my worries, I focused on doing something about them | 10.20% | 7.20% | 10.00% |  | Realistic Thinking \| 0.508 | 57.62% | 0.830 |  |  |
| TYD61* | Cognitive/Perspective | | I allowed myself to be less than perfect | 7.70% | 11.00% | 4.10% |  | Realistic Thinking \| 0.637 | 48.14% | 0.844 |  |  |
|  |  |  |  |  |  |  |  |  |  |  |  |  |
| TYD90*† | Respect/Reflection | | I took time to reflect on myself and how I felt | 0.60% | 0.30% | 2.30% |  | Reflection/solitude \| 0.629 | 55.57% | -- | -- |  |
| TYD96*† | Healthy Routine/Silence, solitude | | I spent time in Silence/ solitude | 0.30% | 0.00% | 0.20% |  | Reflection/solitude \| 0.598 | 50.48% | -- |  |  |
|  |  |  |  |  |  |  |  |  |  |  |  |  |
| TYD29* | Social/Positive People | | I socialised with positive people | 9.40% | 6.60% | 13.20% |  | Social Connections \| 0.547 | 61.06% | 0.698 | 0.769 | 0.460 |
| TYD62∆ | Social/Positive People | | I aimed to spend time with positive people | 9.50% | 5.70% | 12.30% |  | Social Connections \| 0.51 | 55.66% | 0.723 |  |  |
| TYD33* | Social/Talking |  | I had a meaningful conversation with someone | 8.40% | 4.50% | 11.60% |  | Social Connections \| 0.605 | 61.28% | 0.692 |  |  |
| TYD31* | Social/Talking |  | I talked about my day with a friend or a family member | 6.70% | 3.50% | 12.40% |  | Social Connections \| 0.681 | 58.65% | 0.745 |  |  |
|  |  |  |  |  |  |  |  |  |  |  |  |  |
| TYD37*† | Social/Help others | | I did something to help others | 1.60% | 0.40% | 4.50% |  | Social/Help others \| 0.777 | 71.59% | 0.782 | 0.846 | 0.578 |
| TYD51*† | Social/Praise others | | I encouraged or praised someone | 1.00% | 0.50% | 4.80% |  | Social/Help others \| 0.621 | 55.60% | 0.841 |  |  |
| TYD79 | Social/Help others | | I did something to improve the quality of other people's lives | 1.70% | 0.40% | 4.10% |  | Social/Help others \| 0.751 | 68.50% | 0.799 |  |  |
| TYD56* | Social/Kindness others | | I did something kind for someone else | 0.70% | 0.10% | 2.70% |  | Social/Help others \| 0.751 | 68.51% | 0.792 |  |  |
|  |  |  |  |  |  |  |  |  |  |  |  |  |
| TYD58∆ | Social/Positive People | | I arranged to see friends | 4.20% | 2.70% | 5.40% |  | Social/Positive People \| 0.568 | 51.61% | -- | -- |  |
|  |  |  |  |  |  |  |  |  |  |  |  |  |
| TYD63 | Social/Talking |  | I talked with a friend or family member on the phone | 2.50% | 1.40% | 3.90% |  | Social/Talking \| 0.572 | 42.75% | -- | -- |  |
| TYD75 | Social/Social media | | I sent a personal email, text message, or made a post on social media to someone | 2.10% | 1.10% | 2.70% |  | Social/Talking \| 0.509 | 41.71% | -- |  |  |
|  |  |  |  |  |  |  |  |  |  |  |  |  |
| TYD76* | Values/Spiritual | | I acted in a way that is consistent with my personal values | 10.60% | 8.10% | 9.60% |  | Spiritual \| 0.615 | 60.17% | 0.616 | 0.725 | 0.471 |
| TYD77* | Values/Spiritual | | I acted with integrity and dignity | 6.60% | 5.30% | 6.30% |  | Spiritual \| 0.698 | 64.84% | 0.510 |  |  |
| TYD53 | Respect/Others | | I treated others with respect | 1.40% | 1.30% | 2.10% |  | Spiritual \| 0.667 | 53.99% | 0.739 |  |  |
|  |  |  |  |  |  |  |  | **16 (56 items)** | **Σ Eigenvalues = 57.676** |  |  |  |
|  |  |  |  |  |  |  |  |  |  |  |  |  |
| EFA Solution \| Item R2>5% | |  |  |  |  |  |  | (Factor label \| item loading) | EFA Solution \| Item R2>5% | Cronbach's Alpha if Item Deleted | Cronbach's Alpha | Mean Inter-Item Correlations |
| TYD59∆ | Activity/Meaning | | I worked on an activity that was meaningful to me | 9.90% | 5.40% | 9.40% |  | Activity/Enjoyable \| 0.548 | 56.54% | 0.814 | 0.772 | 0.532 |
| TYD34* | Activity/Interesting | | I did a hobby or something that was of interest to me | 9.70% | 7.30% | 5.50% |  | Activity/Enjoyable \| 0.774 | 70.75% | 0.629 |  |  |
| TYD10∆ | Activity/Meaning | | I practiced a skill or did a hobby | 5.70% | 4.50% | 3.90% |  | Activity/Enjoyable \| 0.724 | 63.12% | 0.621 |  |  |
|  |  |  |  |  |  |  |  |  |  |  |  |  |
| TYD58∆ | Social/Positive People | | I arranged to see friends | 4.20% | 2.70% | 5.40% |  | Activity/Laugh/fun \| 0.656 | 51.61% | -- | -- |  |
|  |  |  |  |  |  |  |  |  |  |  |  |  |
| TYD42∆ | Problem Solving | | I broke a large problem into smaller, more manageable steps | 5.20% | 3.50% | 8.40% |  | Activity/Meaning \| 0.549 | 47.00% | 0.649 | 0.717 | 0.391 |
| TYD11∆ | Problem Solving | | I took steps to solve a problem that was affecting me | 4.40% | 2.00% | 6.50% |  | Activity/Meaning \| 0.597 | 52.93% | 0.611 |  |  |
| TYD23* | Activity/Meaning | | I put effort and time into something I wanted to change | 4.40% | 2.20% | 5.20% |  | Activity/Meaning\| 0.614 | 54.90% | 0.635 |  |  |
| TYD83∆ | Healthy Routine/Finances | | I did something to improve or maintain my financial health | 4.00% | 1.90% | 5.60% |  | Activity/Meaning\| 0.561 | 47.66% | 0.720 |  |  |
|  |  |  |  |  |  |  |  |  |  |  |  |  |
| TYD55 | Cognitive/Challenging | | I talked myself out of negative thinking | 5.90% | 4.00% | 5.50% |  | Cognitive/Challenging \| 0.513 | 54.95% | 0.553 | 0.681 | 0.417 |
| TYD27 | Cognitive/Challenging | | I identified unhelpful thoughts and tried to replace them with more helpful ones | 5.50% | 3.80% | 5.60% |  | Cognitive/Challenging \| 0.56 | 59.25% | 0.476 |  |  |
| TYD86* | Values/Spiritual | | I did something to improve or maintain my spiritual wellbeing | 5.10% | 3.10% | 5.50% |  | Cognitive/Challenging \| 0.586 | 51.59% | 0.715 |  |  |
|  |  |  |  |  |  |  |  |  |  |  |  |  |
| TYD08* | Gratitude/Acceptance | | I accepted a situation for what it is | 7.40% | 9.60% | 7.30% |  | Cognitive/Self-perception \| 0.667 | 50.70% | -- | -- |  |
| TYD65∆ | Gratitude/Acceptance | | I tried to accept things that I couldn’t control or change | 7.90% | 9.20% | 7.60% |  | Cognitive/Self-perception \| 0.714 | 59.67% | -- |  |  |
|  |  |  |  |  |  |  |  |  |  |  |  |  |
| TYD20∆ | Emotion Regulation/Avoid chaos | | I avoided chaos in my life | 5.50% | 6.80% | 3.70% |  | Emotion Regulation \| 0.574 | 42.05% | -- | -- |  |
| TYD95∆ | Emotion Regulation/Expression | | I was able to say no when I did not want to do something | 5.00% | 5.20% | 4.60% |  | Emotion Regulation \| 0.529 | 48.27% | -- |  |  |
|  |  |  |  |  |  |  |  |  |  |  |  |  |
| TYD54* | Plan/Future |  | I had something to look forward to | 22.50% | 14.40% | 25.90% |  | Goals/Plans \| 0.516 | 62.72% | -- | -- |  |
| TYD72* | Activity/Achieve goal | | I did something to help me achieve my goals | 9.60% | 4.30% | 11.90% |  | Goals/Plans \| 0.703 | 65.91% | -- |  |  |
| TYD47* | Plan/Execute |  | I made a plan and stuck to it | 10.00% | 5.60% | 10.10% |  | Goals/Plans \| 0.545 | 54.03% | -- |  |  |
|  |  |  |  |  |  |  |  |  |  |  |  |  |
| TYD05* | Healthy Routine/Sleep | | I went to bed and woke up at a regular time | 12.50% | 6.60% | 7.40% |  | Healthy Routine/Chores \| 0.557 | 47.26% | -- | -- |  |
| TYD73* | Healthy Routine/Chores | | I did work or chores around where I live (e.g., house, apartment, etc) | 4.90% | 2.10% | 5.30% |  | Healthy Routine/Chores \| 0.588 | 65.32% | -- |  |  |
|  |  |  |  |  |  |  |  |  |  |  |  |  |
| TYD24* | Healthy Routine/Organised | | I kept my home, living space, or workspace clean and organised | 4.90% | 2.10% | 5.20% |  | Healthy Routine/Chores \| 0.668 | 56.44% | -- | -- |  |
|  |  |  |  |  |  |  |  |  |  |  |  |  |
| TYD85* | Healthy Routine/Sunlight | | I got regular exposure to sunlight (e.g., 15-30 mins) | 6.90% | 4.80% | 4.70% |  | Healthy Routine/Outside \| 0.74 | 74.76% | -- | -- |  |
| TYD30* | Healthy Routine/Outside | | I spent time outside | 7.30% | 5.20% | 4.20% |  | Healthy Routine/Outside \| 0.749 | 74.70% | -- |  |  |
|  |  |  |  |  |  |  |  |  |  |  |  |  |
| TYD91* | Healthy Routine/Physical health | | I did something to improve or maintain my physical health | 8.20% | 5.10% | 5.30% |  | Healthy Routine/Physical health \| 0.789 | 76.01% | 0.943 | 0.943 | 0.767 |
| TYD74* | Healthy Routine/Exercise | | I did some form of exercise (e.g. swimming, went for a walk, etc) | 6.50% | 3.80% | 4.20% |  | Healthy Routine/Physical health \| 0.836 | 79.59% | 0.931 |  |  |
| TYD07∆ | Healthy Routine/Exercise | | I planned or stuck to an exercise routine | 6.40% | 3.50% | 4.20% |  | Healthy Routine/Physical health \| 0.853 | 78.04% | 0.938 |  |  |
| TYD50∆ | Healthy Routine/Exercise | | I did 30 minutes of exercise | 5.60% | 3.20% | 3.80% |  | Healthy Routine/Physical health \| 0.859 | 81.62% | 0.917 |  |  |
|  |  |  |  |  |  |  |  |  |  |  |  |  |
| TYD88* | Activity/Enjoyable | | I did something enjoyable | 18.80% | 14.60% | 17.50% |  | Meaningful Activity \| 0.61 | 66.32% | 0.864 | 0.882 | 0.517 |
| TYD66* | Activity/Satisfying | | I did something that was very satisfying to me | 17.80% | 12.10% | 16.30% |  | Meaningful Activity \| 0.593 | 65.31% | 0.859 |  |  |
| TYD44* | Activity/Laugh, fun | | I had a good laugh or did something that was fun | 14.30% | 11.70% | 14.40% |  | Meaningful Activity \| 0.52 | 55.78% | 0.875 |  |  |
| TYD82* | Values/Spiritual | | I did something to help me live my "ideal" life | 12.10% | 6.70% | 17.60% |  | Meaningful Activity \| 0.609 | 62.62% | 0.863 |  |  |
| TYD35* | Activity/Meaning | | I spent time doing something I believed in | 12.50% | 7.40% | 13.50% |  | Meaningful Activity \| 0.533 | 63.87% | 0.872 |  |  |
| TYD78* | Activity/Improve quality of life | | I did something to improve or maintain the quality of my life | 10.40% | 6.90% | 11.60% |  | Meaningful Activity \| 0.578 | 64.47% | 0.860 |  |  |
| TYD81∆ | Healthy Routine/General satisfaction | | I did something to improve my satisfaction with my life | 10.40% | 6.00% | 12.00% |  | Meaningful Activity \| 0.592 | 67.79% | 0.858 |  |  |
|  |  |  |  |  |  |  |  |  |  |  |  |  |
| TYD70* | Cognitive/Perspective | | I kept a realistic perspective on things | 18.10% | 21.10% | 12.20% |  | Realistic Thinking \| 0.68 | 61.44% | 0.736 | 0.797 | 0.444 |
| TYD32∆ | Cognitive/Perspective | | I tried to keep things in perspective | 12.10% | 11.60% | 11.40% |  | Realistic Thinking \| 0.616 | 59.45% | 0.738 |  |  |
| TYD68* | Cognitive/Challenging | | I stopped myself from thinking unhelpful or unrealistic thoughts | 11.20% | 11.10% | 7.70% |  | Realistic Thinking \| 0.568 | 58.71% | 0.758 |  |  |
| TYD38∆ | Cognitive/Problem solving | | Instead of thinking about my worries, I focused on doing something about them | 10.20% | 7.20% | 10.00% |  | Realistic Thinking \| 0.535 | 57.62% | 0.770 |  |  |
| TYD61* | Cognitive/Perspective | | I allowed myself to be less than perfect | 7.70% | 11.00% | 4.10% |  | Realistic Thinking \| 0.623 | 48.14% | 0.788 |  |  |
|  |  |  |  |  |  |  |  |  |  |  |  |  |
| TYD29* | Social/Positive People | | I socialised with positive people | 9.40% | 6.60% | 13.20% |  | Social Connections \| 0.667 | 61.06% | 0.698 | 0.769 | 0.460 |
| TYD62∆ | Social/Positive People | | I aimed to spend time with positive people | 9.50% | 5.70% | 12.30% |  | Social Connections \| 0.611 | 55.66% | 0.723 |  |  |
| TYD33* | Social/Talking |  | I had a meaningful conversation with someone | 8.40% | 4.50% | 11.60% |  | Social Connections \| 0.555 | 61.28% | 0.692 |  |  |
| TYD31* | Social/Talking |  | I talked about my day with a friend or a family member | 6.70% | 3.50% | 12.40% |  | Social Connections \| 0.646 | 58.65% | 0.745 |  |  |
|  |  |  |  |  |  |  |  |  |  |  |  |  |
| TYD76* | Values/Spiritual | | I acted in a way that is consistent with my personal values | 10.60% | 8.10% | 9.60% |  | Spiritual \| 0.695 | 60.17% | -- | -- |  |
| TYD77* | Values/Spiritual | | I acted with integrity and dignity | 6.60% | 5.30% | 6.30% |  | Spiritual \| 0.718 | 64.84% | -- |  |  |
|  |  |  |  |  |  |  |  | **11 (45 items)** | **Σ Eigenvalues = 56.331** |  |  |  |
|  |  |  |  |  |  |  |  |  |  |  |  |  |
| EFA solution \| Item R2>10% | | | | | | |  | (Factor label \| item loading) | Commonalities \| Item R2>10% | Cronbach's Alpha if Item Deleted | Cronbach's Alpha | Mean Inter-Item Correlations |
| TYD54* | Plan/Future |  | I had something to look forward to | 22.50% | 14.40% | 25.90% |  | Goals/Plans \| 0.595 | 61.56% | 0.707 | 0.786 | 0.478 |
| TYD14∆ | Plan/Future |  | I gave myself things to look forward to | 17.40% | 10.80% | 18.70% |  | Goals/Plans \| 0.592 | 61.31% | 0.704 |  |  |
| TYD72* | Activity/Achieve goal | | I did something to help me achieve my goals | 9.60% | 4.30% | 11.90% |  | Goals/Plans\| 0.758 | 67.15% | 0.744 |  |  |
| TYD47* | Plan/Execute |  | I made a plan and stuck to it | 10.00% | 5.60% | 10.10% |  | Goals/Plans \| 0.543 | 48.69% | 0.775 |  |  |
|  |  |  |  |  |  |  |  |  |  |  |  |  |
| TYD02* | Healthy Routine/General | | I kept a healthy daily routine | 15.80% | 8.20% | 11.80% |  | Healthy Routine \| 0.729 | 70.11% | 0.445 | 0.679 | 0.416 |
| TYD05* | Healthy Routine/Sleep | | I went to bed and woke up at a regular time | 12.50% | 6.60% | 7.40% |  | Healthy Routine \| 0.727 | 57.30% | 0.641 |  |  |
| TYD64* | Healthy Routine/Nutrition | | I prepared and ate a healthy meal | 10.80% | 5.70% | 6.40% |  | Healthy Routine \| 0.671 | 53.34% | 0.653 |  |  |
|  |  |  |  |  |  |  |  |  |  |  |  |  |
| TYD88* | Activity/Enjoyable | | I did something enjoyable | 18.80% | 14.60% | 17.50% |  | Meaningful Activity \| 0.728 | 67.61% | 0.880 | 0.893 | 0.511 |
| TYD66* | Activity/Satisfying | | I did something that was very satisfying to me | 17.80% | 12.10% | 16.30% |  | Meaningful Activity \| 0.679 | 67.65% | 0.875 |  |  |
| TYD44* | Activity/Laugh, fun | | I had a good laugh or did something that was fun | 14.30% | 11.70% | 14.40% |  | Meaningful Activity \| 0.651 | 60.28% | 0.888 |  |  |
| TYD82* | Values/Spiritual | | I did something to help me live my "ideal" life | 12.10% | 6.70% | 17.60% |  | Meaningful Activity \| 0.705 | 65.03% | 0.879 |  |  |
| TYD35* | Activity/Meaning | | I spent time doing something I believed in | 12.50% | 7.40% | 13.50% |  | Meaningful Activity \| 0.535 | 52.16% | 0.879 |  |  |
| TYD39∆ | Activity/Meaning | | I spent time doing something I think is important | 11.10% | 5.50% | 13.20% |  | Meaningful Activity \| 0.545 | 50.76% | 0.882 |  |  |
| TYD78* | Activity/Improve quality of life | | I did something to improve or maintain the quality of my life | 10.40% | 6.90% | 11.60% |  | Meaningful Activity \| 0.681 | 66.38% | 0.876 |  |  |
| TYD81∆ | Healthy Routine/General satisfaction | | I did something to improve my satisfaction with my life | 10.40% | 6.00% | 12.00% |  | Meaningful Activity \| 0.696 | 68.68% | 0.875 |  |  |
|  |  |  |  |  |  |  |  |  |  |  |  |  |
| TYD16* | Respect/Self |  | I treated myself with respect | 25.50% | 19.80% | 17.10% |  | Realistic Thinking \| 0.594 | 56.53% | 0.843 | 0.863 | 0.443 |
| TYD70* | Cognitive/Perspective | | I kept a realistic perspective on things | 18.10% | 21.10% | 12.20% |  | Realistic Thinking \| 0.743 | 62.80% | 0.839 |  |  |
| TYD17* | Cognitive/Future | | Instead of worrying about the past, I focused on my preferred future | 13.80% | 12.90% | 14.20% |  | Realistic Thinking \| 0.549 | 47.67% | 0.846 |  |  |
| TYD32∆ | Cognitive/Perspective | | I tried to keep things in perspective | 12.10% | 11.60% | 11.40% |  | Realistic Thinking \| 0.685 | 60.79% | 0.840 |  |  |
| TYD22* | Emotion Regulation/Coping | | I dealt with feelings of frustration or impatience in a healthy way | 12.60% | 10.70% | 9.80% |  | Realistic Thinking \| 0.528 | 45.65% | 0.848 |  |  |
| TYD68* | Cognitive/Challenging | | I stopped myself from thinking unhelpful or unrealistic thoughts | 11.20% | 11.10% | 7.70% |  | Realistic Thinking \| 0.619 | 49.05% | 0.846 |  |  |
| TYD38∆ | Cognitive/Problem solving | | Instead of thinking about my worries, I focused on doing something about them | 10.20% | 7.20% | 10.00% |  | Realistic Thinking \| 0.556 | 57.77% | 0.847 |  |  |
| TYD61* | Cognitive/Perspective | | I allowed myself to be less than perfect | 7.70% | 11.00% | 4.10% |  | Realistic Thinking \| 0.674 | 53.04% | 0.860 |  |  |
|  |  |  |  |  |  |  |  |  |  |  |  |  |
| TYD29* | Social/Positive People | | I socialised with positive people | 9.40% | 6.60% | 13.20% |  | Social Connections \| 0.589 | 56.22% | 0.698 | 0.769 | 0.460 |
| TYD62∆ | Social/Positive People | | I aimed to spend time with positive people | 9.50% | 5.70% | 12.30% |  | Social Connections \| 0.532 | 50.88% | 0.723 |  |  |
| TYD33* | Social/Talking |  | I had a meaningful conversation with someone | 8.40% | 4.50% | 11.60% |  | Social Connections \| 0.726 | 65.75% | 0.692 |  |  |
| TYD31* | Social/Talking |  | I talked about my day with a friend or a family member | 6.70% | 3.50% | 12.40% |  | Social Connections \| 0.764 | 63.81% | 0.745 |  |  |
|  |  |  |  |  |  |  |  | **5 (27 items)** | **Σ Eigenvalues = 56.090** |  |  |  |
|  |  |  |  |  |  |  |  |  |  |  |  |  |
| EFA solution \| Item R2>15% | | | | | | | | (Factor label \| item loading) | Commonalities \| Item R2>15% |  |  |  |
| TYD54* | Plan/Future |  | I had something to look forward to | 22.50% | 14.40% | 25.90% |  | Composite \| 0.787 | 62.01% | 0.843 | 0.867 | 0.450 |
| TYD16* | Respect/Self |  | I treated myself with respect | 25.50% | 19.80% | 17.10% |  | Composite \| 0.699 | 48.61% | 0.852 |  |  |
| TYD70* | Cognitive/Perspective | | I kept a realistic perspective on things | 18.10% | 21.10% | 12.20% |  | Composite \| 0.621 | 38.48% | 0.861 |  |  |
| TYD88* | Activity/Enjoyable | | I did something enjoyable | 18.80% | 14.60% | 17.50% |  | Composite \| 0.766 | 58.67% | 0.846 |  |  |
| TYD14∆ | Plan/Future |  | I gave myself things to look forward to | 17.40% | 10.80% | 18.70% |  | Composite \| 0.802 | 64.27% | 0.840 |  |  |
| TYD66* | Activity/Satisfying | | I did something that was very satisfying to me | 17.80% | 12.10% | 16.30% |  | Composite \| 0.785 | 61.60% | 0.844 |  |  |
| TYD82* | Values/Spiritual | | I did something to help me live my "ideal" life | 12.10% | 6.70% | 17.60% |  | Composite \| 0.7 | 48.98% | 0.853 |  |  |
| TYD02* | Healthy Routine/General | | I kept a healthy daily routine | 15.80% | 8.20% | 11.80% |  | Composite \| 0.598 | 35.53% | 0.864 |  |  |
|  |  |  |  |  |  |  |  | **1 Factor (8 items)** | **Σ Eigenvalues = 52.268** |  |  |  |
