# Supplementary material for: Measures of Daily Activities Associated With Mental Health (Things You Do Questionnaire): Development of a Preliminary Psychometric Study and Replication Study
Source: JMIR Form Res. 2022 Jul 5;6(7):e38837. doi: 10.2196/38837 (PMC9297144; doi:10.2196/38837)
Supplement: Multimedia Appendix 6 [file formative_v6i7e38837_app6.docx]

| **Multimedia Appendix 6.**  **Table 1.** Invariance test solutions examining dimensionality in Study 1 sample. | | | | | | | | | | | | |
| --- | --- | --- | --- | --- | --- | --- | --- | --- | --- | --- | --- | --- |
| Subgroup considered | Test statistic |  | Configural model |  | Metric model |  | Scalar model |  | Strict model |  | Conclusion drawn |  |
| Reliability CFA - (5 randomised  cross validation  sample spit) | Observed-expected Fit; (χ2:df) < 3:1 |  | 1.168 |  | 1.326 |  | 1.285 |  | 1.248 |  | CFA met Strict invariance criteria |  |
|  | Comparative fit index (CFI) > .90 |  | 0.998 |  | 0.996 |  | 0.997 |  | 0.997 |  |  |  |
|  | Tucker‐Lewis index (TLI) > .90 |  | 0.998 |  | 0.996 |  | 0.997 |  | 0.997 |  |  |  |
|  | RMSEA 90% CI value less than .08 |  | 0.013 |  | 0.021 |  | 0.019 |  | 0.018 |  |  |  |
|  | Comparative Model fit ꭓ^2^∆/df test (p-value) | | | 0.453 |  | 0.464 |  | 0.623 |  |  |  |  |
|  |  |  |  |  |  |  |  |  |  |  |  |  |
| Age groups - <30; 30-45;  45-65; 65+ | Observed-expected Fit; (χ2:df) < 3:1 |  | 1.356 |  | 1.589 |  | -- |  | -- |  | Similarities in factor formation & item loading across groups |  |
|  | Comparative fit index (CFI) > .90 |  | 0.997 |  | 0.959 |  | -- |  | -- |  |  |  |
|  | Tucker‐Lewis index (TLI) > .90 |  | 0.996 |  | 0.957 |  | -- |  | -- |  |  |  |
|  | RMSEA 90% CI value less than .08 |  | 0.022 |  | 0.034 |  | -- |  | -- |  |  |  |
|  | Comparative Model fit ꭓ^2^∆/df test (p-value) | | | 0.168 |  | <0.001 | | -- |  |  |  |  |
|  |  |  |  |  |  |  |  |  |  |  |  |  |
| PHQ-9 Severity groups - Min-Mild (<10);  Moderate (10-14);  Severe (15+) | Observed-expected Fit; (χ2:df) < 3:1 |  | 1.938 |  | 2.188 |  | -- |  | -- |  | Subgroup similarities in factor formation & item loading |  |
|  | Comparative fit index (CFI) > .90 |  | 0.991 |  | 0.946 |  | -- |  | -- |  |  |  |
|  | Tucker‐Lewis index (TLI) > .90 |  | 0.990 |  | 0.942 |  | -- |  | -- |  |  |  |
|  | RMSEA 90% CI value less than .08 |  | 0.031 |  | 0.038 |  | -- |  | -- |  |  |  |
|  | Comparative Model fit ꭓ^2^∆/df test (p-value) | | | 0.01 |  | -- |  | -- |  |  |  |  |
|  |  |  |  |  |  |  |  |  |  |  |  |  |
| GAD-7 Severity groups -  Min-Mild (<10);  Moderate (10-14);  Severe (15+) | Observed-expected Fit; (χ2:df) < 3:1 |  | 1.852 |  | -- |  | -- |  | -- |  | Subgroup similarities in factor formation only |  |
|  | Comparative fit index (CFI) > .90 |  | 0.993 |  | -- |  | -- |  | -- |  |  |  |
|  | Tucker‐Lewis index (TLI) > .90 |  | 0.992 |  | -- |  | -- |  | -- |  |  |  |
|  | RMSEA 90% CI value less than .08 |  | 0.030 |  | -- |  | -- |  | -- |  |  |  |
|  | Comparative Model fit ꭓ^2^∆/df test (p-value) | | | <0.001 | | -- |  | -- |  |  |  |  |
|  |  |  |  |  |  |  |  |  |  |  |  |  |
| Education reported - Tertiary ; Other | Observed-expected Fit; (χ2:df) < 3:1 |  | 2.376 |  | 2.948 |  | -- |  | -- |  | Subgroup similarities in factor formation & item loading |  |
|  | Comparative fit index (CFI) > .90 |  | 0.994 |  | 0.958 |  | -- |  | -- |  |  |  |
|  | Tucker‐Lewis index (TLI) > .90 |  | 0.993 |  | 0.954 |  | -- |  | -- |  |  |  |
|  | RMSEA 90% CI value less than .08 |  | 0.031 |  | 0.038 |  | -- |  | -- |  |  |  |
|  | Comparative Model fit ꭓ^2^∆/df test (p-value) | | | 0.459 |  | <0.001 | | -- |  |  |  |  |
|  |  |  |  |  |  |  |  |  |  |  |  |  |
| Employment reported - Employment; Other | Observed-expected Fit; (χ2:df) < 3:1 |  | 2.362 |  | -- |  | -- |  | -- |  | Subgroup similarities in factor formation only |  |
|  | Comparative fit index (CFI) > .90 |  | 0.994 |  | -- |  | -- |  | -- |  |  |  |
|  | Tucker‐Lewis index (TLI) > .90 |  | 0.993 |  | -- |  | -- |  | -- |  |  |  |
|  | RMSEA 90% CI value less than .08 |  | 0.031 |  | -- |  | -- |  | -- |  |  |  |
|  | Comparative Model fit ꭓ^2^∆/df test (p-value) | | | <0.001 | | -- |  | -- |  |  |  |  |
|  |  |  |  |  |  |  |  |  |  |  |  |  |
| Gender; Male, female (Other) | Observed-expected Fit; (χ2:df) < 3:1 |  | 2.382 |  | 2.471 |  | -- |  | -- |  | Similarities in factor formation & item loading across groups |  |
|  | Comparative fit index (CFI) > .90 |  | 0.994 |  | 0.951 |  | -- |  | -- |  |  |  |
|  | Tucker‐Lewis index (TLI) > .90 |  | 0.993 |  | 0.947 |  | -- |  | -- |  |  |  |
|  | RMSEA 90% CI value less than .08 |  | 0.031 |  | 0.038 |  | -- |  | -- |  |  |  |
|  | Comparative Model fit ꭓ^2^∆/df test (p-value) | | | 0.021 |  | <0.001 | | -- |  |  |  |  |

CFA – Confirmatory factor analysis. All models were based on weighted least square estimators
