# Supplementary material for: Measures of Daily Activities Associated With Mental Health (Things You Do Questionnaire): Development of a Preliminary Psychometric Study and Replication Study
Source: JMIR Form Res. 2022 Jul 5;6(7):e38837. doi: 10.2196/38837 (PMC9297144; doi:10.2196/38837)
Supplement: Multimedia Appendix 7 [file formative_v6i7e38837_app7.docx]

| **Multimedia Appendix 7**  **Table 1.** EFA parameter solution replications across key sample dimensions and methods of rotation. | | | | | | | | | | | | | | | | | | | | |
| --- | --- | --- | --- | --- | --- | --- | --- | --- | --- | --- | --- | --- | --- | --- | --- | --- | --- | --- | --- | --- |
|  |  |  |  |  | Subgroup analysis (analysis of factor relevance across sample dimensions) | | | | | | | | | |  |  | Measurement methodology (Rotation) |  | |  |
|  |  |  |  |  | Baseline symptoms | |  | Age | | | |  | Gender | |  | Oblimin rotation (accounting for factor intercorrelation) | | | |  |
| Item | Primary/Secondary Clusters | Item R2→PHQ9 | Item R2→GAD7 | Item R2→SWLS | Factors \| Non Clin range (PHQ9<10) | Factors \| Clin range (PHQ9 ≥10) |  | Factors \| Age <30 | Factors \| Age 30 to 45 | Factors \| Age 45 to 60 | Factors \| Age 60+ |  | Factors \| Male (& other) | Factors \| Female |  | EFA under no selection criteria (Factor \| item loading) | EFA under r>5% item selection criteria  (Factor \| item loading) | EFA under r>10% item selection criteria  (Factor \| item loading) |  |  |
| TYD88 | Activity/Enjoyable | 18.8% | 14.6% | 17.5% | 3 \| 0.733 | 3 \| 0.745 |  | 3 \| 0.749 | 3 \| 0.688 | 3 \| 0.767 | 1 \| 0.617 |  | 3 \| 0.726 | 3 \| 0.718 |  | 3 \| -0.578 | 6 \| 0.563 | 5 \| -0.61 |  |  |
| TYD66 | Activity/Satisfying | 17.8% | 12.1% | 16.3% | 3 \| 0.647 | 3 \| 0.71 |  | 3 \| 0.659 | 3 \| 0.627 | 3 \| 0.684 | 1 \| 0.664 |  | 3 \| 0.686 | 3 \| 0.672 |  | 3 \| -0.52 | 6 \| 0.547 | 5 \| -0.536 |  |  |
| TYD44 | Activity/Laugh, fun | 14.3% | 11.7% | 14.4% | 3 \| 0.661 | 3 \| 0.625 |  | 3 \| 0.685 | 3 \| 0.656 | 3 \| 0.627 | 1 \| 0.529 |  | 3 \| 0.687 | 3 \| 0.587 |  |  |  | 5 \| -0.519 |  |  |
| TYD35 | Activity/Meaning | 12.5% | 7.4% | 13.5% | 6 \| 0.527 | -- |  | 1 \| 0.58 | 1 \| 0.578 | -- | 1 \| 0.74 |  | -- | 1 \| 0.555 |  |  |  | 1 \| 0.524 |  |  |
| TYD89 | Activity/Avoid stagnant | 13.0% | 7.6% | 10.0% |  |  |  |  |  | 1 \| 0.521 | 1 \| 0.534 |  |  |  |  |  |  |  |  |  |
| TYD39 | Activity/Meaning | 11.1% | 5.5% | 13.2% | 6 \| 0.502 | 1 \| 0.517 |  | 1 \| 0.606 | 1 \| 0.543 |  | 1 \| 0.724 |  | 1 \| 0.517 | 1 \| 0.554 |  |  |  | 1 \| 0.529 |  |  |
| TYD78 | Activity/Improve quality of life | 10.4% | 6.9% | 11.6% | 1 \| 0.68 | 1 \| 0.68 |  | 1 \| 0.686 | 1 \| 0.656 | 1 \| 0.703 | 1 \| 0.662 |  | 1 \| 0.644 | 1 \| 0.684 |  |  |  | 1 \| 0.742 |  |  |
| TYD72 | Activity/Achieve goal | 9.6% | 4.3% | 11.9% | 1 \| 0.737 | 1 \| 0.749 |  | 1 \| 0.758 | 1 \| 0.755 | 1 \| 0.759 | 1 \| 0.653 |  | 1 \| 0.766 | 1 \| 0.751 |  | 1 \| 0.611 | 1 \| 0.659 | 1 \| 0.836 |  |  |
| TYD59 | Activity/Meaning | 9.9% | 5.4% | 9.4% | -- | -- |  | -- | -- | -- | -- |  | -- | -- |  |  | 6 \| 0.543 |  |  |  |
| TYD34 | Activity/Interesting | 9.7% | 7.3% | 5.5% | -- | -- |  | -- | -- | -- | -- |  | -- | -- |  | 3 \| -0.792 | 6 \| 0.806 |  |  |  |
| TYD13 | Activity/Meaning | 7.8% | 6.9% | 6.3% | -- | -- |  | -- | -- | -- | -- |  | -- | -- |  | 3 \| -0.501 |  |  |  |  |
| TYD10 | Activity/Meaning | 5.7% | 4.5% | 3.9% | -- | -- |  | -- | -- | -- | -- |  | -- | -- |  | 3 \| -0.731 | 6 \| 0.76 |  |  |  |
| TYD23 | Activity/Meaning | 4.4% | 2.2% | 5.2% | -- | -- |  | -- | -- | -- | -- |  | -- | -- |  |  | 1 \| 0.519 |  |  |  |
| TYD71 | Activity/Learn new | 1.8% | 1.2% | 2.6% | -- | -- |  | -- | -- | -- | -- |  | -- | -- |  |  |  |  |  |  |
|  |  |  |  |  |  |  |  |  |  |  |  |  |  |  |  |  |  |  |  |  |
| TYD70 | Cognitive/Perspective | 18.1% | 21.1% | 12.2% | 2 \| 0.763 | 2 \| 0.722 |  | 2 \| 0.761 | 2 \| 0.744 | 2 \| 0.69 | 3 \| 0.73 |  | 2 \| 0.743 | 2 \| 0.748 |  | 14 \| 0.63 | 4 \| 0.598 | 3 \| 0.788 |  |  |
| TYD17 | Cognitive/Future | 13.8% | 12.9% | 14.2% | 2 \| 0.526 | -- |  | 2 \| 0.588 | 2 \| 0.556 | 2 \| 0.558 | -- |  | 2 \| 0.533 | 2 \| 0.551 |  |  |  | 3 \| 0.516 |  |  |
| TYD32 | Cognitive/Perspective | 12.1% | 11.6% | 11.4% | 2 \| 0.704 | 2 \| 0.654 |  | 2 \| 0.682 | 2 \| 0.667 | 2 \| 0.625 | 3 \| 0.588 |  | 2 \| 0.702 | 2 \| 0.658 |  | 14 \| 0.532 | 4 \| 0.503 | 3 \| 0.673 |  |  |
| TYD68 | Cognitive/Challenging | 11.2% | 11.1% | 7.7% | 2 \| 0.521 | 2 \| 0.623 |  | 2 \| 0.595 | 2 \| 0.625 | 2 \| 0.661 | 2 \| 0.675 |  | 2 \| 0.546 | 2 \| 0.675 |  | 14 \| 0.559 |  | 3 \| 0.636 |  |  |
| TYD38 | Cognitive/Problem solving | 10.2% | 7.2% | 10.0% | 1 \| 0.585 | 1 \| 0.551 |  | 1 \| 0.518 | 1 \| 0.538 | 1 \| 0.552 | 2 \| 0.551 |  | 1 \| 0.591 | 1 \| 0.541 |  |  |  | 1 \| 0.51 |  |  |
| TYD61 | Cognitive/Perspective | 7.7% | 11.0% | 4.1% | 2 \| 0.664 | 2 \| 0.648 |  | 2 \| 0.615 | 2 \| 0.653 | 2 \| 0.723 | 3 \| 0.675 |  | 2 \| 0.667 | 2 \| 0.681 |  | 14 \| 0.646 | 4 \| 0.582 | 3 \| 0.799 |  |  |
| TYD55 | Cognitive/Challenging | 5.9% | 4.0% | 5.5% | -- | -- |  | -- | -- | -- | -- |  | -- | -- |  |  | 9 \| 0.559 |  |  |  |
| TYD27 | Cognitive/Challenging | 5.5% | 3.8% | 5.6% | -- | -- |  | -- | -- | -- | -- |  | -- | -- |  |  | 9 \| 0.598 |  |  |  |
|  |  |  |  |  |  |  |  |  |  |  |  |  |  |  |  |  |  |  |  |  |
| TYD22 | Emotion Regulation/Coping | 12.6% | 10.7% | 9.8% | -- | 2 \| 0.51 |  | -- | 2 \| 0.507 | 2 \| 0.533 | -- |  | -- | 2 \| 0.582 |  |  |  |  |  |  |
| TYD12 | Emotion Regulation/No excuses | 8.7% | 7.2% | 5.6% | -- | -- |  | -- | -- | -- | -- |  | -- | -- |  |  |  |  |  |  |
| TYD92 | Emotion Regulation/Expression | 5.7% | 4.7% | 8.0% | -- | -- |  | -- | -- | -- | -- |  | -- | -- |  |  |  |  |  |  |
| TYD20 | Emotion Regulation/Avoid chaos | 5.5% | 6.8% | 3.7% | -- | -- |  | -- | -- | -- | -- |  | -- | -- |  |  | 11 \| 0.596 |  |  |  |
| TYD95 | Emotion Regulation/Expression | 5.0% | 5.2% | 4.6% | -- | -- |  | -- | -- | -- | -- |  | -- | -- |  |  | 11 \| 0.506 |  |  |  |
| TYD25 | Emotion Regulation/Coping | 4.9% | 2.3% | 6.7% | -- | -- |  | -- | -- | -- | -- |  | -- | -- |  |  |  |  |  |  |
| TYD41 | Emotion Regulation/Patience | 2.9% | 3.3% | 3.5% | -- | -- |  | -- | -- | -- | -- |  | -- | -- |  |  |  |  |  |  |
| TYD43 | Emotion Regulation/Pushing through | 3.6% | 1.6% | 4.6% | -- | -- |  | -- | -- | -- | -- |  | -- | -- |  |  |  |  |  |  |
| TYD52 | Emotion Regulation/Realistic promises | 2.0% | 2.3% | 1.4% | -- | -- |  | -- | -- | -- | -- |  | -- | -- |  |  |  |  |  |  |
| TYD28 | Emotion Regulation/Pushing through | 0.4% | 1.6% | 0.0% | -- | -- |  | -- | -- | -- | -- |  | -- | -- |  | 10 \| 0.651 |  |  |  |  |
| TYD60 | Emotion Regulation/Pushing through | 0.0% | 1.1% | 0.7% | -- | -- |  | -- | -- | -- | -- |  | -- | -- |  | 10 \| 0.766 |  |  |  |  |
| TYD57 | Emotion Regulation/Coping | 0.3% | 0.0% | 1.3% | -- | -- |  | -- | -- | -- | -- |  | -- | -- |  | 10 \| 0.556 |  |  |  |  |
| TYD15 | Emotion Regulation/Pushing through | 0.1% | 0.2% | 0.8% | -- | -- |  | -- | -- | -- | -- |  | -- | -- |  | 10 \| 0.667 |  |  |  |  |
|  |  |  |  |  |  |  |  |  |  |  |  |  |  |  |  |  |  |  |  |  |
| TYD80 | Environment | 3.9% | 1.8% | 4.8% | -- | -- |  | -- | -- | -- | -- |  | -- | -- |  |  |  |  |  |  |
|  |  |  |  |  |  |  |  |  |  |  |  |  |  |  |  |  |  |  |  |  |
| TYD65 | Gratitude/Acceptance | 7.9% | 9.2% | 7.6% | -- | -- |  | -- | -- | -- | -- |  | -- | -- |  | 14 \| 0.681 | 4 \| 0.666 |  |  |  |
| TYD08 | Gratitude/Acceptance | 7.4% | 9.6% | 7.3% | -- | -- |  | -- | -- | -- | -- |  | -- | -- |  | 14 \| 0.604 | 4 \| 0.642 |  |  |  |
| TYD01 | Gratitude/Acceptance | 6.8% | 4.2% | 12.3% | -- | -- |  | -- | -- | -- | 1 \| 0.529 |  | -- | -- |  |  |  |  |  |  |
| TYD94 | Gratitude/Acceptance | 1.2% | 1.0% | 1.6% | -- | -- |  | -- | -- | -- | -- |  | -- | -- |  |  |  |  |  |  |
|  |  |  |  |  |  |  |  |  |  |  |  |  |  |  |  |  |  |  |  |  |
| TYD48 | Healthy Routine/Mental wellbeing | 14.1% | 10.8% | 12.1% | -- | -- |  | -- | 3 \| 0.534 | -- | -- |  | 3 \| 0.51 | -- |  |  |  |  |  |  |
| TYD02 | Healthy Routine/General | 15.8% | 8.2% | 11.8% | 5 \| 0.758 | 5 \| 0.731 |  | 5 \| 0.719 | 5 \| 0.727 | 5 \| 0.73 | 5 \| 0.696 |  | 5 \| 0.71 | 5 \| 0.738 |  |  |  | 4 \| 0.789 |  |  |
| TYD81 | Healthy Routine/General satisfaction | 10.4% | 6.0% | 12.0% | 1 \| 0.723 | 1 \| 0.684 |  | 1 \| 0.662 | 1 \| 0.707 | 1 \| 0.707 | 1 \| 0.645 |  | 1 \| 0.66 | 1 \| 0.701 |  | 1 \| 0.524 |  | 1 \| 0.769 |  |  |
| TYD05 | Healthy Routine/Sleep | 12.5% | 6.6% | 7.4% | 5 \| 0.707 | 5 \| 0.687 |  | 5 \| 0.793 | 5 \| 0.679 | 5 \| 0.713 | 5 \| 0.748 |  | 5 \| 0.663 | 5 \| 0.749 |  | 13 \| -0.518 | 7 \| 0.562 | 4 \| 0.816 |  |  |
| TYD64 | Healthy Routine/Nutrition | 10.8% | 5.7% | 6.4% | 5 \| 0.648 | 5 \| 0.668 |  | 5 \| 0.58 | 5 \| 0.662 | 5 \| 0.651 | 5 \| 0.689 |  | 5 \| 0.706 | 5 \| 0.654 |  |  |  | 4 \| 0.738 |  |  |
| TYD91 | Healthy Routine/Physical health | 8.2% | 5.1% | 5.3% | -- | -- |  | -- | -- | -- | -- |  | -- | -- |  | 2 \| 0.832 | 2 \| 0.821 |  |  |  |
| TYD30 | Healthy Routine/Outside | 7.3% | 5.2% | 4.2% | -- | -- |  | -- | -- | -- | -- |  | -- | -- |  | 15 \| 0.698 | 8 \| -0.813 |  |  |  |
| TYD85 | Healthy Routine/Sunlight | 6.9% | 4.8% | 4.7% | -- | -- |  | -- | -- | -- | -- |  | -- | -- |  | 15 \| 0.741 | 8 \| -0.803 |  |  |  |
| TYD74 | Healthy Routine/Exercise | 6.5% | 3.8% | 4.2% | -- | -- |  | -- | -- | -- | -- |  | -- | -- |  | 2 \| 0.89 | 2 \| 0.864 |  |  |  |
| TYD07 | Healthy Routine/Exercise | 6.4% | 3.5% | 4.2% | -- | -- |  | -- | -- | -- | -- |  | -- | -- |  | 2 \| 0.923 | 2 \| 0.922 |  |  |  |
| TYD18 | Healthy Routine/Electronics | 5.4% | 3.8% | 4.1% | -- | -- |  | -- | -- | -- | -- |  | -- | -- |  | 7 \| 0.907 |  |  |  |  |
| TYD67 | Healthy Routine/Hygiene | 6.1% | 2.6% | 4.0% | -- | -- |  | -- | -- | -- | -- |  | -- | -- |  |  |  |  |  |  |
| TYD50 | Healthy Routine/Exercise | 5.6% | 3.2% | 3.8% | -- | -- |  | -- | -- | -- | -- |  | -- | -- |  | 2 \| 0.928 | 2 \| 0.898 |  |  |  |
| TYD73 | Healthy Routine/Chores | 4.9% | 2.1% | 5.3% | -- | -- |  | -- | -- | -- | -- |  | -- | -- |  | 12 \| 0.735 | 7 \| 0.522 |  |  |  |
| TYD24 | Healthy Routine/Organised | 4.9% | 2.1% | 5.2% | -- | -- |  | -- | -- | -- | -- |  | -- | -- |  | 12 \| 0.674 | 7 \| 0.638 |  |  |  |
| TYD83 | Healthy Routine/Finances | 4.0% | 1.9% | 5.6% | -- | -- |  | -- | -- | -- | -- |  | -- | -- |  |  | 1 \| 0.604 |  |  |  |
| TYD36 | Healthy Routine/Social | 4.4% | 3.4% | 3.4% | -- | -- |  | -- | -- | -- | -- |  | -- | -- |  | 7 \| 0.909 |  |  |  |  |
| TYD26 | Healthy Routine/Hydration | 4.2% | 2.9% | 2.9% | -- | -- |  | -- | -- | -- | -- |  | -- | -- |  |  |  |  |  |  |
| TYD21 | Healthy Routine/Sleep | 4.0% | 3.2% | 2.6% | -- | -- |  | -- | -- | -- | -- |  | -- | -- |  |  |  |  |  |  |
| TYD49 | Healthy Routine/Relax | 3.0% | 1.6% | 3.9% | -- | -- |  | -- | -- | -- | -- |  | -- | -- |  |  |  |  |  |  |
| TYD46 | Healthy Routine/Electronics | 2.9% | 2.2% | 2.8% | -- | -- |  | -- | -- | -- | -- |  | -- | -- |  | 7 \| 0.506 |  |  |  |  |
| TYD84 | Healthy Routine/Nutrition | 2.6% | 1.8% | 1.0% | -- | -- |  | -- | -- | -- | -- |  | -- | -- |  |  |  |  |  |  |
| TYD06 | Healthy Routine/Excesses | 2.5% | 1.5% | 1.6% | -- | -- |  | -- | -- | -- | -- |  | -- | -- |  | 9 \| 0.81 |  |  |  |  |
| TYD19 | Healthy Routine/Substance | 2.6% | 1.6% | 1.1% | -- | -- |  | -- | -- | -- | -- |  | -- | -- |  |  |  |  |  |  |
| TYD09 | Healthy Routine/Finances | 2.0% | 1.0% | 1.9% | -- | -- |  | -- | -- | -- | -- |  | -- | -- |  | 16 \| 0.606 |  |  |  |  |
| TYD03 | Healthy Routine/Substance | 0.6% | 0.5% | 0.3% | -- | -- |  | -- | -- | -- | -- |  | -- | -- |  | 9 \| 0.859 |  |  |  |  |
| TYD96 | Healthy Routine/Silence, solitude | 0.3% | 0.0% | 0.2% | -- | -- |  | -- | -- | -- | -- |  | -- | -- |  | 6 \| -0.645 |  |  |  |  |
|  |  |  |  |  |  |  |  |  |  |  |  |  |  |  |  |  |  |  |  |  |
| TYD54 | Plan/Future | 22.5% | 14.4% | 25.9% | 3 \| 0.567 | 3 \| 0.604 |  | 3 \| 0.659 | 3 \| 0.59 | 3 \| 0.512 | 1 \| 0.537 |  | 3 \| 0.575 | 3 \| 0.61 |  |  |  |  |  |  |
| TYD14 | Plan/Future | 17.4% | 10.8% | 18.7% | 3 \| 0.527 | 3 \| 0.633 |  | 3 \| 0.619 | 3 \| 0.606 | 3 \| 0.508 | 1 \| 0.543 |  | 3 \| 0.55 | 3 \| 0.617 |  |  |  |  |  |  |
| TYD40 | Plan/Realistic goals | 11.6% | 7.5% | 13.5% | -- | -- |  | -- | -- | 1 \| 0.548 | -- |  | 1 \| 0.514 | -- |  |  |  |  |  |  |
| TYD45 | Plan/Personal responsibility | 9.4% | 7.2% | 11.8% | -- | -- |  | 1 \| 0.572 | -- | -- | -- |  | 2 \| 0.509 | 1 \| 0.528 |  |  |  |  |  |  |
| TYD47 | Plan/Execute | 10.0% | 5.6% | 10.1% | 1 \| 0.549 | 1 \| 0.532 |  | 1 \| 0.592 | -- | 1 \| 0.532 | -- |  | 1 \| 0.526 | 1 \| 0.558 |  |  |  |  |  |  |
| TYD04 | Plan/Organise | 7.3% | 2.6% | 9.7% | -- | -- |  | -- | -- | -- | -- |  | -- | -- |  |  |  |  |  |  |
|  |  |  |  |  |  |  |  |  |  |  |  |  |  |  |  |  |  |  |  |  |
| TYD42 | Problem Solving | 5.2% | 3.5% | 8.4% | -- | -- |  | -- | -- | -- | -- |  | -- | -- |  |  |  |  |  |  |
| TYD11 | Problem Solving | 4.4% | 2.0% | 6.5% | -- | -- |  | -- | -- | -- | -- |  | -- | -- |  |  | 1 \| 0.501 |  |  |  |
|  |  |  |  |  |  |  |  |  |  |  |  |  |  |  |  |  |  |  |  |  |
| TYD16 | Respect/Self | 25.5% | 19.8% | 17.1% | 2 \| 0.647 | 2 \| 0.534 |  | 2 \| 0.544 | 2 \| 0.599 | 2 \| 0.593 | 3 \| 0.537 |  | 2 \| 0.579 | 2 \| 0.602 |  |  |  | 3 \| 0.567 |  |  |
| TYD69 | Respect/Self | 11.7% | 8.6% | 11.1% | -- | -- |  | -- | 3 \| 0.502 | -- | 2 \| 0.518 |  | -- | -- |  |  |  |  |  |  |
| TYD53 | Respect/Others | 1.4% | 1.3% | 2.1% | -- | -- |  | -- | -- | -- | -- |  | -- | -- |  | 5 \| 0.709 |  |  |  |  |
| TYD90 | Respect/Reflection | 0.6% | 0.3% | 2.3% | -- | -- |  | -- | -- | -- | -- |  | -- | -- |  | 6 \| -0.646 |  |  |  |  |
|  |  |  |  |  |  |  |  |  |  |  |  |  |  |  |  |  |  |  |  |  |
| TYD29 | Social/Positive People | 9.4% | 6.6% | 13.2% | 4 \| 0.573 | 4 \| 0.604 |  | 4 \| 0.685 | -- | 4 \| 0.604 | 4 \| 0.642 |  | -- | 4 \| 0.669 |  |  | 10 \|-0.573 | 2 \| -0.649 |  |  |
| TYD62 | Social/Positive People | 9.5% | 5.7% | 12.3% | 4 \| 0.529 | 4 \| 0.558 |  | 4 \| 0.654 | -- | 4 \| 0.535 | 4 \| 0.565 |  | -- | 4 \| 0.587 |  |  | 10 \|-0.53 | 2 \| -0.567 |  |  |
| TYD33 | Social/Talking | 8.4% | 4.5% | 11.6% | 4 \| 0.699 | 4 \| 0.735 |  | 4 \| 0.745 | 4 \| 0.678 | 4 \| 0.735 | 4 \| 0.785 |  | 4 \| 0.735 | 4 \| 0.71 |  | 8 \| 0.579 | 5 \| 0.62 | 2 \| -0.81 |  |  |
| TYD93 | Social/Improve belonging | 8.8% | 5.8% | 8.9% | -- | -- |  | -- | -- | -- | -- |  | -- | -- |  |  |  |  |  |  |
| TYD31 | Social/Talking | 6.7% | 3.5% | 12.4% | 4 \| 0.769 | 4 \| 0.728 |  | 4 \| 0.668 | 4 \| 0.688 | 4 \| 0.758 | 4 \| 0.725 |  | 4 \| 0.776 | 4 \| 0.727 |  | 8 \| 0.693 | 5 \| 0.738 | 2 \| -0.864 |  |  |
| TYD87 | Social/Improve relationships | 6.7% | 4.0% | 9.6% | -- | -- |  | -- | -- | -- | -- |  | -- | -- |  |  |  |  |  |  |
| TYD58 | Social/Positive People | 4.2% | 2.7% | 5.4% | -- | -- |  | -- | -- | -- | -- |  | -- | -- |  | 11 \|-0.547 | 10 \|-0.624 |  |  |  |
| TYD63 | Social/Talking | 2.5% | 1.4% | 3.9% | -- | -- |  | -- | -- | -- | -- |  | -- | -- |  | 8 \| 0.578 |  |  |  |  |
| TYD37 | Social/Help others | 1.6% | 0.4% | 4.5% | -- | -- |  | -- | -- | -- | -- |  | -- | -- |  | 4 \| 0.814 |  |  |  |  |
| TYD51 | Social/Praise others | 1.0% | 0.5% | 4.8% | -- | -- |  | -- | -- | -- | -- |  | -- | -- |  | 4 \| 0.63 |  |  |  |  |
| TYD79 | Social/Help others | 1.7% | 0.4% | 4.1% | -- | -- |  | -- | -- | -- | -- |  | -- | -- |  | 4 \| 0.792 |  |  |  |  |
| TYD75 | Social/Social media | 2.1% | 1.1% | 2.7% | -- | -- |  | -- | -- | -- | -- |  | -- | -- |  | 8 \| 0.522 |  |  |  |  |
| TYD56 | Social/Kindness others | 0.7% | 0.1% | 2.7% | -- | -- |  | -- | -- | -- | -- |  | -- | -- |  | 4 \| 0.782 |  |  |  |  |
|  |  |  |  |  |  |  |  |  |  |  |  |  |  |  |  |  |  |  |  |  |
| TYD82 | Values/Spiritual | 12.1% | 6.7% | 17.6% | 1 \| 0.723 | 1 \| 0.688 |  | 1 \| 0.732 | 1 \| 0.701 | 1 \| 0.711 | 1 \| 0.675 |  | 1 \| 0.698 | 1 \| 0.697 |  | 1 \| 0.554 | 1 \| 0.549 | 1 \| 0.781 |  |  |
| TYD76 | Values/Spiritual | 10.6% | 8.1% | 9.6% | 2 \| 0.507 | -- |  | -- | -- | 6 \| 0.654 | 3 \| 0.677 |  | 2 \| 0.523 | -- |  | 5 \| 0.641 | 3 \| 0.773 |  |  |  |
| TYD77 | Values/Spiritual | 6.6% | 5.3% | 6.3% | -- | -- |  | -- | -- | -- | -- |  | -- | -- |  | 5 \| 0.731 | 3 \| 0.801 |  |  |  |
| TYD86 | Values/Spiritual | 5.1% | 3.1% | 5.5% | -- | -- |  | -- | -- | -- | -- |  | -- | -- |  |  | 9 \| 0.596 |  |  |  |
|  |  |  |  |  |  |  |  |  |  |  |  |  |  |  |  |  |  |  |  |  |
| Number of factors extracted | | -- | | | 6 | 5 |  | 5 | 5 | 6 | 5 |  | 5 | 5 |  |  |  |  |  |  |
| Model prediction *R^2^* (PHQ9\|GAD7\|SWLS) | | -- | | | 26%\| 19%\| 24% | 11%\| 10%\| 16% |  | 30%\| 26%\| 33% | 35%\| 30%\| 31% | 44%\| 34%\| 33% | 48%\| 38%\| 41% |  | 38%\| 34%\| 35% | 39%\| 30%\| 32% |  |  |  |  |  |  |

TYD: Things You Do; Colours indicate the magnitude of relationship with the outcome measures, with dark blue indicating the least observed relationship and dark red indicating the strongest observed relationship.
