# Supplementary material for: Measures of Daily Activities Associated With Mental Health (Things You Do Questionnaire): Development of a Preliminary Psychometric Study and Replication Study
Source: JMIR Form Res. 2022 Jul 5;6(7):e38837. doi: 10.2196/38837 (PMC9297144; doi:10.2196/38837)
Supplement: Multimedia Appendix 8 [file formative_v6i7e38837_app8.docx]

| **Multimedia Appendix 8**  **Table 1.** Means, Study 1 and Study 2 sample group differences and association of TYDQ items with each of the PHQ-9, GAD-7 and SWLS outcomes | | | | | | | | | | | | | | | | | | | | | |  |  |  |  |
| --- | --- | --- | --- | --- | --- | --- | --- | --- | --- | --- | --- | --- | --- | --- | --- | --- | --- | --- | --- | --- | --- | --- | --- | --- | --- |
|  |  |  | Mean TYD item weekly scores | | | | |  | Item → PHQ9 *R^2^* | |  |  | Item → GAD7 *R^2^* | |  | |  | | Item → SWLS *R^2^* | |  | | |  |  |
| Item order | Primary/Secondary Cluster | Item | Study 1 | Study 2 | ∆% | *p-value* of samples TYD ∆* | Study 1-Study 2 sample ∆ (*R^2^*) |  | Study 1 | Study 2 | *p-value* of samples  *R^2^*∆* |  | Study 1 | Study 2 | *p-value* of samples  *R^2^*∆* | |  | | Study 1 | Study 2 | *p-value* of samples  *R^2^*∆* | | |  |  |
| TYD88 | Activity/Enjoyable | I did something enjoyable | 1.999 | 2.341 | 17% | *<0.001* | 2.0% |  | 19% | 23% | *0.045* |  | 15% | 17% | *0.335* | |  | | 17% | 21% | *0.009* | | |  |  |
| TYD66 | Activity/Satisfying | I did something that was very satisfying to me | 1.665 | 2.028 | 22% | *<0.001* | 2.1% |  | 18% | 23% | *0.187* |  | 12% | 16% | *0.208* | |  | | 16% | 20% | *0.061* | | |  |  |
| TYD44 | Activity/Laugh, fun | I had a good laugh or did something that was fun | 1.669 | 1.929 | 16% | *<0.001* | 1.2% |  | 14% | 21% | *0.024* |  | 12% | 16% | *0.242* | |  | | 14% | 19% | *0.012* | | |  |  |
| TYD35 | Activity/Meaning | I spent time doing something I believed in | 1.894 | 2.181 | 15% | *<0.001* | 1.1% |  | 12% | 18% | *0.001* |  | 7% | 12% | *0.001* | |  | | 13% | 16% | *0.008* | | |  |  |
| TYD89 | Activity/Avoid stagnant | I avoided being 'stagnant' | 1.581 | 1.968 | 24% | *<0.001* | 1.9% |  | 13% | 22% | *<0.001* |  | 8% | 14% | *<0.001* | |  | | 10% | 14% | *0.002* | | |  |  |
| TYD78 | Activity/Improve quality of life | I did something to improve or maintain the quality of my life | 1.623 | 2.294 | 41% | *<0.001* | 6.5% |  | 10% | 18% | *<0.001* |  | 7% | 11% | *0.005* | |  | | 12% | 15% | *0.004* | | |  |  |
| TYD72 | Activity/Achieve goal | I did something to help me achieve my goals | 1.682 | 2.022 | 20% | *<0.001* | 1.7% |  | 10% | 15% | *0.001* |  | 4% | 9% | *<0.001* | |  | | 12% | 15% | *0.007* | | |  |  |
| TYD34 | Activity/Interesting | I did a hobby or something that was of interest to me | 1.658 | 2.050 | 24% | *<0.001* | 2.2% |  | 10% | 14% | *0.036* |  | 7% | 10% | *0.103* | |  | | 5% | 10% | *0.000* | | |  |  |
| TYD23 | Activity/Meaning | I put effort and time into something I wanted to change | 1.549 | 1.803 | 16% | *<0.001* | 1.1% |  | 4% | 8% | *0.007* |  | 2% | 3% | *0.227* | |  | | 5% | 8% | *0.004* | | |  |  |
| TYD71 | Activity/Learn new | I tried to learn something new | 1.484 | 1.574 | 6% | *0.008* | 0.1% |  | 2% | 5% | *<0.001* |  | 1% | 4% | *<0.001* | |  | | 3% | 4% | *0.014* | | |  |  |
|  |  |  |  |  |  |  |  |  |  |  |  |  |  |  |  | |  | |  |  |  | | |  |  |
| TYD70 | Cognitive/Perspective | I kept a realistic perspective on things | 2.325 | 2.564 | 10% | *<0.001* | 1.0% |  | 18% | 24% | *<0.001* |  | 21% | 25% | *<0.001* | |  | | 12% | 17% | *0.000* | | |  |  |
| TYD17 | Cognitive/Future | Instead of worrying about the past, I focused on my preferred future | 1.862 | 2.050 | 10% | *<0.001* | 0.5% |  | 14% | 23% | *<0.001* |  | 13% | 21% | *<0.001* | |  | | 14% | 21% | *0.000* | | |  |  |
| TYD68 | Cognitive/Challenging | I stopped myself from thinking unhelpful or unrealistic thoughts | 1.693 | 1.925 | 14% | *<0.001* | 0.8% |  | 11% | 16% | *0.007* |  | 11% | 15% | *0.029* | |  | | 8% | 10% | *0.033* | | |  |  |
| TYD61 | Cognitive/Perspective | I allowed myself to be less than perfect | 2.385 | 2.485 | 4% | *0.004* | 0.1% |  | 8% | 11% | *0.001* |  | 11% | 15% | *0.002* | |  | | 4% | 7% | *0.000* | | |  |  |
|  |  |  |  |  |  |  |  |  |  |  |  |  |  |  |  | |  | |  |  |  | | |  |  |
| TYD22 | Emotion Regulation/Coping | I dealt with feelings of frustration or impatience in a healthy way | 1.690 | 2.237 | 32% | *<0.001* | 4.6% |  | 13% | 25% | *<0.001* |  | 11% | 24% | *<0.001* | |  | | 10% | 18% | *0.000* | | |  |  |
| TYD92 | Emotion Regulation/Expression | I expressed my feelings honestly, instead of suppressing them | 1.758 | 2.072 | 18% | *<0.001* | 1.4% |  | 6% | 11% | *<0.001* |  | 5% | 8% | *0.017* | |  | | 8% | 11% | *0.011* | | |  |  |
| TYD25 | Emotion Regulation/Coping | I dealt with things that were creating stress | 1.645 | 1.755 | 7% | *<0.001* | 0.2% |  | 5% | 6% | *0.200* |  | 2% | 3% | *0.271* | |  | | 7% | 5% | *0.621* | | |  |  |
| TYD43 | Emotion Regulation/Pushing through | I made myself do something because I knew it would be beneficial | 1.963 | 2.208 | 12% | *<0.001* | 1.0% |  | 4% | 6% | *0.038* |  | 2% | 2% | *0.290* | |  | | 5% | 6% | *0.018* | | |  |  |
| TYD28 | Emotion Regulation/Pushing through | I faced a situation that was unpleasant but necessary | 1.627 | 1.578 | -3% | *0.112* | 0.0% |  | 0% | 0% | *0.483* |  | 2% | 1% | *0.510* | |  | | 0% | 0% | *0.524* | | |  |  |
| TYD60 | Emotion Regulation/Pushing through | I pushed myself to do things that were difficult or triggered some stress | 2.044 | 1.811 | -11% | *<0.001* | 0.9% |  | 0% | 0% | *0.946* |  | 1% | 1% | *0.892* | |  | | 1% | 1% | *0.913* | | |  |  |
|  |  |  |  |  |  |  |  |  |  |  |  |  |  |  |  | |  | |  |  |  | | |  |  |
| TYD80 | Environment | I did something to improve the quality of the physical environment | 1.446 | 1.832 | 27% | *<0.001* | 2.3% |  | 4% | 8% | *0.003* |  | 2% | 4% | *0.025* | |  | | 5% | 9% | *0.002* | | |  |  |
|  |  |  |  |  |  |  |  |  |  |  |  |  |  |  |  | |  | |  |  |  | | |  |  |
| TYD08 | Gratitude/Acceptance | I accepted a situation for what it is | 2.398 | 2.575 | 7% | *<0.001* | 0.5% |  | 7% | 19% | *<0.001* |  | 10% | 21% | *<0.001* | |  | | 7% | 14% | *0.000* | | |  |  |
| TYD01 | Gratitude/Acceptance | I thought about things that I am grateful for | 1.867 | 2.211 | 18% | *<0.001* | 1.7% |  | 7% | 12% | *<0.001* |  | 4% | 8% | *0.001* | |  | | 12% | 16% | *0.000* | | |  |  |
| TYD94 | Gratitude/Acceptance | I accepted my symptoms by allowing them to peak and pass | 1.855 | 2.046 | 10% | *<0.001* | 0.5% |  | 1% | 4% | *<0.001* |  | 1% | 4% | *<0.001* | |  | | 2% | 3% | *0.030* | | |  |  |
|  |  |  |  |  |  |  |  |  |  |  |  |  |  |  |  | |  | |  |  |  | | |  |  |
| TYD48 | Healthy Routine/Mental wellbeing | I did things which are good for my mental wellbeing | 1.919 | 2.295 | 20% | *<0.001* | 2.2% |  | 14% | 22% | *<0.001* |  | 11% | 16% | *0.001* | |  | | 12% | 17% | *0.000* | | |  |  |
| TYD02 | Healthy Routine/General | I kept a healthy daily routine | 1.709 | 2.070 | 21% | *<0.001* | 1.9% |  | 16% | 23% | *<0.001* |  | 8% | 13% | *<0.001* | |  | | 12% | 15% | *0.000* | | |  |  |
| TYD05 | Healthy Routine/Sleep | I went to bed and woke up at a regular time | 1.861 | 2.186 | 17% | *<0.001* | 1.3% |  | 12% | 21% | *<0.001* |  | 7% | 12% | *<0.001* | |  | | 7% | 15% | *0.000* | | |  |  |
| TYD64 | Healthy Routine/Nutrition | I prepared and ate a healthy meal | 2.159 | 2.538 | 18% | *<0.001* | 2.3% |  | 11% | 17% | *<0.001* |  | 6% | 10% | *<0.001* | |  | | 6% | 10% | *0.000* | | |  |  |
| TYD91 | Healthy Routine/Physical health | I did something to improve or maintain my physical health | 1.635 | 2.117 | 29% | *<0.001* | 3.1% |  | 8% | 14% | *<0.001* |  | 5% | 8% | *0.028* | |  | | 5% | 8% | *0.006* | | |  |  |
| TYD30 | Healthy Routine/Outside | I spent time outside | 2.344 | 2.496 | 6% | *<0.001* | 0.3% |  | 7% | 12% | *0.001* |  | 5% | 7% | *0.046* | |  | | 4% | 8% | *0.000* | | |  |  |
| TYD85 | Healthy Routine/Sunlight | I got regular exposure to sunlight (e.g., 15-30 mins) | 2.487 | 2.479 | 0% | *0.811* | 0.0% |  | 7% | 10% | *0.030* |  | 5% | 6% | *0.407* | |  | | 5% | 6% | *0.071* | | |  |  |
| TYD74 | Healthy Routine/Exercise | I did some form of exercise (e.g. swimming, went for a walk, etc) | 1.550 | 2.075 | 34% | *<0.001* | 3.6% |  | 6% | 10% | *0.015* |  | 4% | 6% | *0.090* | |  | | 4% | 7% | *0.021* | | |  |  |
| TYD18 | Healthy Routine/Electronics | I kept my use of electronic devices or games to a healthy level | 1.703 | 1.832 | 8% | *<0.001* | 0.2% |  | 5% | 11% | *<0.001* |  | 4% | 9% | *<0.001* | |  | | 4% | 6% | *0.011* | | |  |  |
| TYD67 | Healthy Routine/Hygiene | I had a bath or shower | 3.450 | 3.410 | -1% | *0.091* | 0.0% |  | 6% | 7% | *0.420* |  | 3% | 2% | *0.659* | |  | | 4% | 4% | *0.589* | | |  |  |
| TYD73 | Healthy Routine/Chores | I did work or chores around where I live (e.g., house, apartment, etc) | 2.498 | 2.803 | 12% | *<0.001* | 1.5% |  | 5% | 8% | *0.001* |  | 2% | 3% | *0.187* | |  | | 5% | 7% | *0.012* | | |  |  |
| TYD24 | Healthy Routine/Organised | I kept my home, living space, or workspace clean and organised | 1.921 | 2.256 | 17% | *<0.001* | 1.5% |  | 5% | 9% | *<0.001* |  | 2% | 4% | *0.005* | |  | | 5% | 7% | *0.013* | | |  |  |
| TYD21 | Healthy Routine/Sleep | I kept a relaxing bedtime routine, that did not involve watching videos or checking social media | 0.997 | 1.276 | 28% | *<0.001* | 1.0% |  | 4% | 8% | *0.009* |  | 3% | 6% | *0.022* | |  | | 3% | 6% | *0.004* | | |  |  |
| TYD49 | Healthy Routine/Relax | I did something to help me relax (e.g., slow breathing, stretching etc) | 1.524 | 1.803 | 18% | *<0.001* | 1.1% |  | 3% | 5% | *0.026* |  | 2% | 3% | *0.146* | |  | | 4% | 5% | *0.066* | | |  |  |
| TYD06 | Healthy Routine/Excesses | I avoided unhealthy habits (e.g., I chose not to have a drink, or gamble, etc) | 2.270 | 2.488 | 10% | *<0.001* | 0.6% |  | 2% | 5% | *0.001* |  | 1% | 3% | *0.004* | |  | | 2% | 3% | *0.013* | | |  |  |
| TYD03 | Healthy Routine/Substance | I had an alcohol free day | 2.855 | 2.776 | -3% | *0.015* | 0.1% |  | 1% | 0% | *0.713* |  | 1% | 0% | *0.351* | |  | | 0% | 0% | *0.638* | | |  |  |
| TYD96 | Healthy Routine/Silence, solitude | I spent time in Silence/ solitude | 2.415 | 2.496 | 3% | *0.025* | 0.1% |  | 0% | 0% | *0.138* |  | 0% | 0% | *0.174* | |  | | 0% | 0% | *0.808* | | |  |  |
|  |  |  |  |  |  |  |  |  |  |  |  |  |  |  |  | |  | |  |  |  | | |  |  |
| TYD54 | Plan/Future | I had something to look forward to | 1.864 | 2.024 | 9% | *<0.001* | 0.4% |  | 22% | 27% | *0.017* |  | 14% | 19% | *0.005* | |  | | 26% | 29% | *0.003* | | |  |  |
| TYD40 | Plan/Realistic goals | I set realistic and achievable goals | 1.683 | 1.973 | 17% | *<0.001* | 1.2% |  | 12% | 18% | *<0.001* |  | 7% | 12% | *0.002* | |  | | 14% | 17% | *0.003* | | |  |  |
| TYD45 | Plan/Personal responsibility | I took responsibility for the direction of my life | 2.402 | 2.596 | 8% | *<0.001* | 0.5% |  | 9% | 16% | *<0.001* |  | 7% | 13% | *<0.001* | |  | | 12% | 16% | *0.000* | | |  |  |
| TYD47 | Plan/Execute | I made a plan and stuck to it | 1.675 | 1.948 | 16% | *<0.001* | 1.2% |  | 10% | 15% | *0.001* |  | 6% | 7% | *0.123* | |  | | 10% | 13% | *0.004* | | |  |  |
| TYD04 | Plan/Organise | I took steps to organise what I did each day | 2.005 | 2.317 | 16% | *<0.001* | 1.4% |  | 7% | 15% | *<0.001* |  | 3% | 7% | *<0.001* | |  | | 10% | 12% | *0.000* | | |  |  |
|  |  |  |  |  |  |  |  |  |  |  |  |  |  |  |  | |  | |  |  |  | | |  |  |
| TYD16 | Respect/Self | I treated myself with respect | 2.175 | 2.506 | 15% | *<0.001* | 1.5% |  | 26% | 38% | *<0.001* |  | 20% | 31% | *0.000* | |  | | 17% | 23% | *0.000* | | |  |  |
| TYD69 | Respect/Self | I praised myself when I did something well | 1.340 | 1.628 | 22% | *<0.001* | 1.2% |  | 12% | 14% | *0.543* |  | 9% | 10% | *0.689* | |  | | 11% | 14% | *0.108* | | |  |  |
| TYD90 | Respect/Reflection | I took time to reflect on myself and how I felt | 1.951 | 2.164 | 11% | *<0.001* | 0.6% |  | 1% | 2% | *0.004* |  | 0% | 1% | *0.049* | |  | | 2% | 3% | *0.185* | | |  |  |
|  |  |  |  |  |  |  |  |  |  |  |  |  |  |  |  | |  | |  |  |  | | |  |  |
| TYD29 | Social/Positive People | I socialised with positive people | 1.679 | 1.885 | 12% | *<0.001* | 0.7% |  | 9% | 13% | *0.121* |  | 7% | 8% | *0.428* | |  | | 13% | 18% | *0.014* | | |  |  |
| TYD33 | Social/Talking | I had a meaningful conversation with someone | 1.883 | 2.092 | 11% | *<0.001* | 0.7% |  | 8% | 14% | *0.001* |  | 4% | 9% | *0.002* | |  | | 12% | 18% | *0.000* | | |  |  |
| TYD31 | Social/Talking | I talked about my day with a friend or a family member | 2.109 | 2.258 | 7% | *<0.001* | 0.3% |  | 7% | 11% | *0.001* |  | 3% | 6% | *0.039* | |  | | 12% | 19% | *0.000* | | |  |  |
| TYD37 | Social/Help others | I did something to help others | 2.281 | 2.238 | -2% | *0.171* | 0.0% |  | 2% | 4% | *0.006* |  | 0% | 2% | *0.003* | |  | | 4% | 6% | *0.069* | | |  |  |
| TYD51 | Social/Praise others | I encouraged or praised someone | 2.550 | 2.554 | 0% | *0.893* | 0.0% |  | 1% | 2% | *0.072* |  | 0% | 1% | *0.543* | |  | | 5% | 6% | *0.032* | | |  |  |
| TYD56 | Social/Kindness others | I did something kind for someone else | 2.231 | 2.210 | -1% | *0.480* | 0.0% |  | 1% | 2% | *0.004* |  | 0% | 1% | *0.005* | |  | | 3% | 4% | *0.018* | | |  |  |
|  |  |  |  |  |  |  |  |  |  |  |  |  |  |  |  | |  | |  |  |  | | |  |  |
| TYD82 | Values/Spiritual | I did something to help me live my "ideal" life | 1.423 | 1.712 | 20% | *<0.001* | 1.2% |  | 12% | 16% | *0.078* |  | 7% | 11% | *0.015* | |  | | 18% | 24% | *0.002* | | |  |  |
| TYD76 | Values/Spiritual | I acted in a way that is consistent with my personal values | 2.992 | 3.111 | 4% | *<0.001* | 0.3% |  | 11% | 15% | *<0.001* |  | 8% | 11% | *<0.001* | |  | | 10% | 12% | *0.000* | | |  |  |
| TYD77 | Values/Spiritual | I acted with integrity and dignity | 3.172 | 3.191 | 1% | *0.450* | 0.0% |  | 7% | 9% | *0.004* |  | 5% | 7% | *0.020* | |  | | 6% | 7% | *0.095* | | |  |  |
| TYD86 | Values/Spiritual | I did something to improve or maintain my spiritual wellbeing | 1.132 | 1.433 | 27% | *<0.001* | 1.2% |  | 5% | 7% | *0.517* |  | 3% | 6% | *0.138* | |  | | 6% | 6% | *0.771* | | |  |  |
| *Analysis of variance (ANOVA) comparing group means (Study 1 vs. Study 2); GAD-7: Generalized Anxiety Disorder-7 Item Scale; PHQ-9: Patient Health Questionnaire-9 Item; SWLS: Satisfaction with Life Scale. ∆ denotes group differences | | | | | | | | | | | | | | | |  | |  | | | | |  | |  |
