# Supplementary material for: Measures of Daily Activities Associated With Mental Health (Things You Do Questionnaire): Development of a Preliminary Psychometric Study and Replication Study
Source: JMIR Form Res. 2022 Jul 5;6(7):e38837. doi: 10.2196/38837 (PMC9297144; doi:10.2196/38837)
Supplement: Multimedia Appendix 9 [file formative_v6i7e38837_app9.docx]

**Multimedia Appendix 9**

| **Table 1** – Exploratory factor analysis solution and assigned factor labels for items grouped with no item selection criteria (96 items), and under *R^2^*>/10% outcomes association criteria | | | | | | | | | | | | | | | | | | |  |
| --- | --- | --- | --- | --- | --- | --- | --- | --- | --- | --- | --- | --- | --- | --- | --- | --- | --- | --- | --- |
|  |  |  | |  | | |  | | | |  | | | EFA solution with no item selection criteria | | | EFA solution under *R^2^*>10 item selection criteria | |  |
|  |  |  | | Item→PHQ9 *R^2^* | | | Item→GAD7 *R^2^* | | | | Item→SWLS *R^2^* | | | (Factor \| item loading) | | (Factor \| item loading) | | |  |
| Item order | Primary/Secondary Cluster | Item |  | | Study 1 | Study 2 | | Study 1 | Study 2 | Study 1 | | Study 2 | Study 1 Re-Analysed (Common items with Study 2) | | Study 2 | Study 1 Re-Analysed  (Common items with Study 2) | | Study 2 | |
| TYD88 | Activity/Enjoyable | I did something enjoyable | | | 19% | 23% | | 15% | 17% | 17% | | 21% | Activity/Enjoyable \| 0.605 | | Activity/Enjoyable \| 0.61 | Activity \| 0.728 | | Activity \| 0.586 | |
| TYD66 | Activity/Satisfying | I did something that was very satisfying to me | | | 18% | 23% | | 12% | 16% | 16% | | 20% | Activity/Enjoyable \| 0.541 | | Activity/Enjoyable \| 0.593 | Activity \| 0.679 | | Activity \| 0.673 | |
| TYD44 | Activity/Laugh, fun | I had a good laugh or did something that was fun | | | 14% | 21% | | 12% | 16% | 14% | | 19% | λ≥0.5 | | Mixed social/future \| 0.52 | Activity \| 0.651 | | λ≥0.5 | |
| TYD35 | Activity/Meaning | I spent time doing something I believed in | | | 12% | 18% | | 7% | 12% | 13% | | 16% | Activity/Meaning \| 0.516 | | Activity/Enjoyable \| 0.533 | Meaningful activity \| 0.535 | | Activity \| 0.566 | |
| TYD89 | Activity/Avoid stagnant | I avoided being 'stagnant' | | | 13% | 22% | | 8% | 14% | 10% | | 14% | λ≥0.5 | | λ≥0.5 | λ≥0.5 | | λ≥0.5 | |
| TYD78 | Activity/Improve quality of life | I did something to improve or maintain the quality of my life | | | 10% | 18% | | 7% | 11% | 12% | | 15% | Activity/Meaning \| 0.637 | | Activity/Meaning \| 0.578 | Meaningful activity \| 0.681 | | Activity \| 0.573 | |
| TYD72 | Activity/Achieve goal | I did something to help me achieve my goals | | | 10% | 15% | | 4% | 9% | 12% | | 15% | Activity/Meaning \| 0.729 | | Activity/Meaning \| 0.703 | Meaningful activity \| 0.758 | | Activity \| 0.674 | |
| TYD34 | Activity/Interesting | I did a hobby or something that was of interest to me | | | 10% | 14% | | 7% | 10% | 5% | | 10% | Activity/Enjoyable \| 0.753 | | Activity/Enjoyable \| 0.774 | λ≥0.5 | | Activity \| 0.565 | |
| TYD23 | Activity/Meaning | I put effort and time into something I wanted to change | | | 4% | 8% | | 2% | 3% | 5% | | 8% | Activity/Meaning \| 0.606 | | Activity/Meaning \| 0.614 | -- | | -- | |
| TYD71 | Activity/Learn new | I tried to learn something new | | | 2% | 5% | | 1% | 4% | 3% | | 4% | λ≥0.5 | | λ≥0.5 | -- | | -- | |
|  |  |  | | |  |  | |  |  |  | |  |  | |  |  | |  | |
| TYD70 | Cognitive/Perspective | I kept a realistic perspective on things | | | 18% | 24% | | 21% | 25% | 12% | | 17% | Cognitive/Self-perception \| 0.686 | | Cognitive/Self-perception \| 0.68 | Cognitive \| 0.743 | | Cognitive \| 0.696 | |
| TYD17 | Cognitive/Future | Instead of worrying about the past, I focused on my preferred future | | | 14% | 23% | | 13% | 21% | 14% | | 21% | Cognitive/Self-perception \| 0.513 | | λ≥0.5 | Cognitive \| 0.549 | | Cognitive \| 0.517 | |
| TYD68 | Cognitive/Challenging | I stopped myself from thinking unhelpful or unrealistic thoughts | | | 11% | 16% | | 11% | 15% | 8% | | 10% | Cognitive/Self-perception \| 0.62 | | Cognitive/Self-perception \| 0.568 | Cognitive \| 0.619 | | Cognitive \| 0.554 | |
| TYD61 | Cognitive/Perspective | I allowed myself to be less than perfect | | | 8% | 11% | | 11% | 15% | 4% | | 7% | Cognitive/Self-perception \| 0.637 | | Cognitive/Self-perception \| 0.623 | Cognitive \| 0.674 | | Cognitive \| 0.693 | |
|  |  |  | | |  |  | |  |  |  | |  |  | |  |  | |  | |
| TYD22 | Emotion Regulation/Coping | I dealt with feelings of frustration or impatience in a healthy way | | | 13% | 25% | | 11% | 24% | 10% | | 18% | λ≥0.5 | | λ≥0.5 | Cognitive \| 0.528 | | Cognitive \| 0.619 | |
| TYD92 | Emotion Regulation/Expression | I expressed my feelings honestly, instead of suppressing them | | | 6% | 11% | | 5% | 8% | 8% | | 11% | λ≥0.5 | | λ≥0.5 | -- | | -- | |
| TYD25 | Emotion Regulation/Coping | I dealt with things that were creating stress | | | 5% | 6% | | 2% | 3% | 7% | | 5% | λ≥0.5 | | λ≥0.5 | -- | | -- | |
| TYD43 | Emotion Regulation/Pushing through | I made myself do something because I knew it would be beneficial | | | 4% | 6% | | 2% | 2% | 5% | | 6% | λ≥0.5 | | λ≥0.5 | -- | | -- | |
| TYD28 | Emotion Regulation/Pushing through | I faced a situation that was unpleasant but necessary | | | 0% | 0% | | 2% | 1% | 0% | | 0% | Emotion Regulation \| 0.63 | | λ≥0.5 | -- | | -- | |
| TYD60 | Emotion Regulation/Pushing through | I pushed myself to do things that were difficult or triggered some stress | | | 0% | 0% | | 1% | 1% | 1% | | 1% | Emotion Regulation \| 0.734 | | λ≥0.5 | -- | | -- | |
|  |  |  | | |  |  | |  |  |  | |  |  | |  |  | |  | |
| TYD80 | Environment | I did something to improve the quality of the physical environment | | | 4% | 8% | | 2% | 4% | 5% | | 9% | λ≥0.5 | | λ≥0.5 |  | |  | |
|  |  |  | | |  |  | |  |  |  | |  |  | |  |  | |  | |
| TYD08 | Gratitude/Acceptance | I accepted a situation for what it is | | | 7% | 19% | | 10% | 21% | 7% | | 14% | Cognitive/Self-perception \| 0.635 | | Cognitive/Self-perception \| 0.667 | λ≥0.5 | | Cognitive \| 0.707 | |
| TYD01 | Gratitude/Acceptance | I thought about things that I am grateful for | | | 7% | 12% | | 4% | 8% | 12% | | 16% | λ≥0.5 | | λ≥0.5 | -- | | -- | |
| TYD94 | Gratitude/Acceptance | I accepted my symptoms by allowing them to peak and pass | | | 1% | 4% | | 1% | 4% | 2% | | 3% | λ≥0.5 | | λ≥0.5 | -- | | -- | |
|  |  |  | | |  |  | |  |  |  | |  |  | |  |  | |  | |
| TYD48 | Healthy Routine/Mental wellbeing | I did things which are good for my mental wellbeing | | | 14% | 22% | | 11% | 16% | 12% | | 17% | λ≥0.5 | | λ≥0.5 | λ≥0.5 | | Activity \| 0.501 | |
| TYD02 | Healthy Routine/General | I kept a healthy daily routine | | | 16% | 23% | | 8% | 13% | 12% | | 15% | λ≥0.5 | | λ≥0.5 | Health routine \| 0.729 | | Health routine \| 0.58 | |
| TYD05 | Healthy Routine/Sleep | I went to bed and woke up at a regular time | | | 12% | 21% | | 7% | 12% | 7% | | 15% | Healthy Routine/Sleep \| 0.559 | | Mixed Routine/Sleep/chores \| 0.557 | Health routine \| 0.727 | | λ≥0.5 | |
| TYD64 | Healthy Routine/Nutrition | I prepared and ate a healthy meal | | | 11% | 17% | | 6% | 10% | 6% | | 10% | λ≥0.5 | | λ≥0.5 | Health routine \| 0.671 | | λ≥0.5 | |
| TYD91 | Healthy Routine/Physical health | I did something to improve or maintain my physical health | | | 8% | 14% | | 5% | 8% | 5% | | 8% | Healthy Routine/exercise \| 0.787 | | Healthy Routine/Exercise \| 0.789 | λ≥0.5 | | Health routine \| 0.748 | |
| TYD30 | Healthy Routine/Outside | I spent time outside | | | 7% | 12% | | 5% | 7% | 4% | | 8% | Healthy Routine/Outside \| 0.69 | | Healthy Routine/Outside \| 0.749 | λ≥0.5 | | Health routine \| 0.709 | |
| TYD85 | Healthy Routine/Sunlight | I got regular exposure to sunlight (e.g., 15-30 mins) | | | 7% | 10% | | 5% | 6% | 5% | | 6% | Healthy Routine/Outside \| 0.73 | | Healthy Routine/Outside \| 0.74 | λ≥0.5 | | λ≥0.5 | |
| TYD74 | Healthy Routine/Exercise | I did some form of exercise (e.g. swimming, went for a walk, etc) | | | 6% | 10% | | 4% | 6% | 4% | | 7% | Healthy Routine/exercise \| 0.836 | | Healthy Routine/Exercise \| 0.836 | λ≥0.5 | | Health routine \| 0.819 | |
| TYD18 | Healthy Routine/Electronics | I kept my use of electronic devices or games to a healthy level | | | 5% | 11% | | 4% | 9% | 4% | | 6% | Healthy Routine/social media \| 0.821 | | λ≥0.5 | -- | | -- | |
| TYD67 | Healthy Routine/Hygiene | I had a bath or shower | | | 6% | 7% | | 3% | 2% | 4% | | 4% | λ≥0.5 | | λ≥0.5 | -- | | -- | |
| TYD73 | Healthy Routine/Chores | I did work or chores around where I live (e.g., house, apartment, etc) | | | 5% | 8% | | 2% | 3% | 5% | | 7% | Healthy Routine/Chores \| 0.704 | | Mixed Routine/Sleep/chores \| 0.588 | -- | | -- | |
| TYD24 | Healthy Routine/Organised | I kept my home, living space, or workspace clean and organised | | | 5% | 9% | | 2% | 4% | 5% | | 7% | Healthy Routine/Chores \| 0.662 | | Mixed Routine/Sleep/chores \| 0.668 | -- | | -- | |
| TYD21 | Healthy Routine/Sleep | I kept a relaxing bedtime routine, that did not involve watching videos or checking social media | | | 4% | 8% | | 3% | 6% | 3% | | 6% | λ≥0.5 | | λ≥0.5 | -- | | -- | |
| TYD49 | Healthy Routine/Relax | I did something to help me relax (e.g., slow breathing, stretching etc) | | | 3% | 5% | | 2% | 3% | 4% | | 5% | λ≥0.5 | | λ≥0.5 | -- | | -- | |
| TYD06 | Healthy Routine/Excesses | I avoided unhealthy habits (e.g., I chose not to have a drink, or gamble, etc) | | | 2% | 5% | | 1% | 3% | 2% | | 3% | Healthy Routine Substance \| 0.781 | | λ≥0.5 | -- | | -- | |
| TYD03 | Healthy Routine/Substance | I had an alcohol free day | | | 1% | 0% | | 1% | 0% | 0% | | 0% | Healthy Routine Substance \| 0.814 | | λ≥0.5 | -- | | -- | |
| TYD96 | Healthy Routine/Silence, solitude | I spent time in Silence/ solitude | | | 0% | 0% | | 0% | 0% | 0% | | 0% | Reflection/solitude \| 0.598 | | λ≥0.5 | -- | | -- | |
|  |  |  | | |  |  | |  |  |  | |  |  | |  |  | |  | |
| TYD54 | Plan/Future | I had something to look forward to | | | 22% | 27% | | 14% | 19% | 26% | | 29% | λ≥0.5 | | Mixed social/future \| 0.516 | Activity \| 0.595 | | Activity \| 0.564 | |
| TYD40 | Plan/Realistic goals | I set realistic and achievable goals | | | 12% | 18% | | 7% | 12% | 14% | | 17% | λ≥0.5 | | λ≥0.5 | λ≥0.5 | | Meaningful activity \| 0.556 | |
| TYD45 | Plan/Personal responsibility | I took responsibility for the direction of my life | | | 9% | 16% | | 7% | 13% | 12% | | 16% | λ≥0.5 | | λ≥0.5 | λ≥0.5 | | λ≥0.5 | |
| TYD47 | Plan/Execute | I made a plan and stuck to it | | | 10% | 15% | | 6% | 7% | 10% | | 13% | λ≥0.5 | | Activity/Meaning \| 0.545 | Meaningful activity \| 0.543 | | Meaningful activity \| 0.664 | |
| TYD04 | Plan/Organise | I took steps to organise what I did each day | | | 7% | 15% | | 3% | 7% | 10% | | 12% | λ≥0.5 | | λ≥0.5 | -- | | Meaningful activity \| 0.751 | |
|  |  |  | | |  |  | |  |  |  | |  |  | |  |  | |  | |
| TYD16 | Respect/Self | I treated myself with respect | | | 26% | 38% | | 20% | 31% | 17% | | 23% | Cognitive/Self-perception \| 0.518 | | λ≥0.5 | Cognitive \| 0.594 | | Cognitive \| 0.656 | |
| TYD69 | Respect/Self | I praised myself when I did something well | | | 12% | 14% | | 9% | 10% | 11% | | 14% | λ≥0.5 | | λ≥0.5 | λ≥0.5 | | λ≥0.5 | |
| TYD90 | Respect/Reflection | I took time to reflect on myself and how I felt | | | 1% | 2% | | 0% | 1% | 2% | | 3% | Reflection/solitude \| 0.629 | | λ≥0.5 | -- | | -- | |
|  |  |  | | |  |  | |  |  |  | |  |  | |  |  | |  | |
| TYD29 | Social/Positive People | I socialised with positive people | | | 9% | 13% | | 7% | 8% | 13% | | 18% | Social/Positive People \| 0.547 | | Mixed social/future \| 0.667 | Social \| 0.589 | | Social \| 0.672 | |
| TYD33 | Social/Talking | I had a meaningful conversation with someone | | | 8% | 14% | | 4% | 9% | 12% | | 18% | Social/Talking \| 0.605 | | Social/talking \| 0.555 | Social \| 0.726 | | Social \| 0.766 | |
| TYD31 | Social/Talking | I talked about my day with a friend or a family member | | | 7% | 11% | | 3% | 6% | 12% | | 19% | Social/Talking \| 0.681 | | Social/talking \| 0.646 | Social \| 0.764 | | Social \| 0.781 | |
| TYD37 | Social/Help others | I did something to help others | | | 2% | 4% | | 0% | 2% | 4% | | 6% | Social/Help others \| 0.777 | | λ≥0.5 | -- | | -- | |
| TYD51 | Social/Praise others | I encouraged or praised someone | | | 1% | 2% | | 0% | 1% | 5% | | 6% | Social/Help others \| 0.621 | | λ≥0.5 | -- | | -- | |
| TYD56 | Social/Kindness others | I did something kind for someone else | | | 1% | 2% | | 0% | 1% | 3% | | 4% | Social/Help others \| 0.751 | | λ≥0.5 | -- | | -- | |
|  |  |  | | |  |  | |  |  |  | |  |  | |  |  | |  | |
| TYD82 | Values/Spiritual | I did something to help me live my "ideal" life | | | 12% | 16% | | 7% | 11% | 18% | | 24% | Activity/Meaning \| 0.679 | | Activity/Meaning \| 0.609 | Meaningful activity \| 0.705 | | Activity \| 0.673 | |
| TYD76 | Values/Spiritual | I acted in a way that is consistent with my personal values | | | 11% | 15% | | 8% | 11% | 10% | | 12% | Spiritual \| 0.615 | | Spiritual a \| 0.695 | λ≥0.5 | | λ≥0.5 | |
| TYD77 | Values/Spiritual | I acted with integrity and dignity | | | 7% | 9% | | 5% | 7% | 6% | | 7% | Spiritual \| 0.698 | | Spiritual a \| 0.718 | -- | | -- | |
| TYD86 | Values/Spiritual | I did something to improve or maintain my spiritual wellbeing | | | 5% | 7% | | 3% | 6% | 6% | | 6% | λ≥0.5 | | Spiritual b \| 0.586 | -- | | -- | |
| **Number of factors extracted** | |  | | | | **--** | | |  |  | | **15 (36 items)** | | | **10 (28 items)** | **5 (21 items)** | | **5 (26 items)** | |
|  |  |  |  | |  |  | |  |  |  | |  | | |  |  | |  | |

*Items retained in Study 1 and Study 2; non-flagged items were explored in Study 1 only; TYD – things you do; λ denotes factor loading with weak loading items (<.5) suppressed from the table and consequent CFA analyses; EFA – exploratory factor analysis; *R* denotes a correlation coefficient and % variance explained (*R^2^*)
