# Supplementary material for: Measures of Daily Activities Associated With Mental Health (Things You Do Questionnaire): Development of a Preliminary Psychometric Study and Replication Study
Source: JMIR Form Res. 2022 Jul 5;6(7):e38837. doi: 10.2196/38837 (PMC9297144; doi:10.2196/38837)
Supplement: Multimedia Appendix 10 [file formative_v6i7e38837_app10.docx]

| **Multimedia Appendix 10**  **Table 1.** A heat map of the Identified factor association to PHQ-9, GAD-7 and SWLS outcomes across Study 1 and Study 2 samples. | | | | | | | | | | | | | | | | | | | | | | | | | | | |
| --- | --- | --- | --- | --- | --- | --- | --- | --- | --- | --- | --- | --- | --- | --- | --- | --- | --- | --- | --- | --- | --- | --- | --- | --- | --- | --- | --- |
|  | |  | Not at all | 1-2 day \| Week | Half the week | Most | Daily |  |  |  | Not at all | 1-2 day \| Week | Half the week | Most | Daily |  |  |  | Not at all | 1-2 day \| Week | Half the week | Most | Daily |  |  | |  |
|  | |  | (0) | (1) | (2) | (3) | (4) |  |  |  | (0) | (1) | (2) | (3) | (4) |  |  |  | (0) | (1) | (2) | (3) | (4) |  |  | |  |
| Factor (number of items) | | |  | |  |  |  | *R* | *R* | *p-value* |  | |  |  |  | *R* | *R* | *p-value* |  | |  |  |  | *R* | *R* | | *p-value* |
|  | |  | Item → PHQ-9 mean scores | | | | | *bivariate* | *part* | *part* | Item → GAD-7 mean scores | | | | | *bivariate* | *part* | *part* | Item → SWLS mean scores | | | | | *bivariate* | *part* | | *part* |
| Goals and Plans | | Study 1 (Testing) | 15.4 | 12.5 | 9.7 | 7.1 | 5.9 | -0.41 | 0.03 | 0.050 | 11.5 | 10.0 | 8.2 | 6.2 | 5.6 | -0.312 | 0.074 | <0.001 | 12.7 | 16.4 | 19.8 | 23.1 | 24.6 | 0.444 | 0.061 | | <0.001 |
| (5 items) | | Study 2 (Replication) | 15.4 | 11.7 | 8.4 | 5.5 | 3.7 | -0.49 | 0.03 | 0.016 | 11.1 | 8.9 | 6.8 | 4.7 | 3.2 | -0.382 | 0.073 | <0.001 | 13.0 | 17.0 | 20.9 | 24.4 | 27.2 | 0.487 | 0.036 | | 0.010 |
|  | |  |  |  |  |  |  |  |  |  |  |  |  |  |  |  |  |  |  |  |  |  |  |  |  | |  |
| Realistic Thinking | | Study 1 (Testing) | 17.9 | 14.3 | 11.2 | 7.7 | 4.6 | -0.51 | -0.21 | <0.001 | 14.5 | 11.9 | 9.2 | 6.1 | 3.6 | -0.503 | -0.295 | <0.001 | 11.9 | 14.9 | 17.9 | 21.7 | 24.5 | 0.433 | 0.112 | | <0.001 |
| (6 items) | | Study 2 (Replication) | 19.8 | 14.5 | 10.0 | 5.6 | 3.1 | -0.61 | -0.24 | <0.001 | 15.5 | 11.7 | 8.0 | 4.4 | 2.0 | -0.594 | -0.341 | <0.001 | 12.3 | 14.9 | 19.0 | 23.7 | 26.9 | 0.503 | 0.122 | | <0.001 |
|  | |  |  |  |  |  |  |  |  |  |  |  |  |  |  |  |  |  |  |  |  |  |  |  |  | |  |
| Meaningful Activities | | Study 1 (Testing) | 17.9 | 13.6 | 10.2 | 7.6 | 4.6 | -0.50 | -0.20 | <0.001 | 13.8 | 10.8 | 8.6 | 6.3 | 4.1 | -0.431 | -0.182 | <0.001 | 11.3 | 15.1 | 18.8 | 22.3 | 25.7 | 0.504 | 0.177 | | <0.001 |
| (4 items) | | Study 2 (Replication) | 17.0 | 12.5 | 9.1 | 5.6 | 3.2 | -0.55 | -0.14 | <0.001 | 12.1 | 9.6 | 7.4 | 4.7 | 2.6 | -0.470 | -0.127 | <0.001 | 12.3 | 16.2 | 19.8 | 24.3 | 27.4 | 0.535 | 0.128 | | <0.001 |
|  | |  |  |  |  |  |  |  |  |  |  |  |  |  |  |  |  |  |  |  |  |  |  |  |  | |  |
| Social Connections | | Study 1 (Testing) | 15.4 | 12.6 | 10.2 | 8.6 | 6.8 | -0.34 | 0.00 | 0.917 | 11.7 | 10.1 | 8.3 | 7.5 | 6.0 | -0.258 | 0.029 | 0.065 | 12.4 | 15.7 | 19.0 | 21.9 | 24.2 | 0.419 | 0.114 | | <0.001 |
| (3 items) | | Study 2 (Replication) | 14.5 | 11.1 | 8.4 | 6.3 | 4.6 | -0.42 | -0.02 | 0.057 | 10.7 | 8.4 | 6.8 | 5.4 | 4.0 | -0.322 | 0.020 | 0.156 | 13.6 | 16.8 | 20.8 | 24.1 | 27.0 | 0.496 | 0.160 | | <0.001 |
|  | |  |  |  |  |  |  |  |  |  |  |  |  |  |  |  |  |  |  |  |  |  |  |  |  | |  |
| Healthy Routines | | Study 1 (Testing) | 16.8 | 13.1 | 10.3 | 7.8 | 5.8 | -0.45 | -0.20 | <0.001 | 12.4 | 10.2 | 8.5 | 7.0 | 5.5 | -0.324 | -0.104 | <0.001 | 13.3 | 16.2 | 19.1 | 21.1 | 23.0 | 0.349 | 0.084 | | <0.001 |
| (3 items) | | Study 2 (Replication) | 16.4 | 12.9 | 9.7 | 5.7 | 3.4 | -0.55 | -0.22 | <0.001 | 11.5 | 9.5 | 7.7 | 4.9 | 3.2 | -0.419 | -0.108 | <0.001 | 13.2 | 16.5 | 19.9 | 23.4 | 26.1 | 0.444 | 0.105 | | <0.001 |
| **Model *R^2^* (5 factors – 21 items)** | | | |  |  |  |  | ***R^2^ (*Study 1*)* 37.1%** | | |  |  |  |  |  | ***R^2^ (*Study 1*) 30.0%*** | | |  |  |  |  |  | ***R^2^ (*Study 1*) 32.1%*** | | | |
|  | |  |  |  |  |  |  | ***R^2^ (Study 2) 46.8%*** | | |  |  |  |  |  | ***R^2^ (Study 2) 38.2%*** | | |  |  |  |  |  | ***R^2^ (Study 2) 38.1%*** | | | |
|  | |  |  |  |  |  |  |  |  |  |  |  |  |  |  |  |  |  |  |  |  |  |  |  |  | |  |
|  | |  |  |  |  |  |  |  |  |  |  |  |  |  |  |  |  |  |  |  |  |  |  |  |  | |  |
| Composite factor | | Study 1 (Testing) | 18.8 | 14.3 | 10.2 | 6.0 | 3.6 | -0.58 | -0.58 | 0.00 | 14.1 | 11.4 | 8.4 | 5.4 | 3.2 | -0.493 | -0.493 | <0.001 | 10.6 | 14.5 | 19.1 | 23.5 | 26.9 | 0.552 | 0.552 | | <0.001 |
| (7 items) | | Study 2 (Replication) | 18.6 | 13.9 | 8.9 | 4.8 | 2.4 | -0.63 | -0.63 | 0.00 | 13.7 | 10.5 | 7.3 | 4.0 | 1.9 | -0.541 | -0.541 | <0.001 | 11.2 | 15.1 | 19.9 | 25.1 | 28.4 | 0.583 | 0.583 | | <0.001 |
|  | |  |  |  |  |  |  |  |  |  |  |  |  |  |  |  |  |  |  |  |  |  |  |  |  | |  |
| **Model *R^2^* (1 factor – 7 items)** | | |  |  |  |  |  | ***R^2^ (*Study 1*) 33.5%*** | | |  |  |  |  |  | ***R^2^ (*Study 1*) -*24.3%** | | |  |  |  |  |  | ***R^2^ (*Study 1*)* 30.5%** | | | |
|  | |  |  |  |  |  |  | ***R^2^ (*Study 2*) 39.8%*** | | |  |  |  |  |  | ***R^2^ (*Study 2*) -*29.2%** | | |  |  |  |  |  | ***R^2^ (*Study 2*)* 34.0%** | | | |
|  | |  |  |  |  |  |  |  |  |  |  |  |  |  |  |  |  |  |  |  |  |  |  |  |  | |  |
|  | | |  | | | | |  |  |  |  | | | | |  |  |  |  | | | | |  |  | |  |
| Binary probability estimates | | | Item → probability PHQ-9≥10 | | | | | AUC | OR | p-value | Item → probability GAD-7≥10 | | | | | AUC | OR | p-value | Item → probability SWLS≥10 | | | | | AUC | OR | | p-value |
| Goals and Plans | | Study 1 (Testing) | 82% | 67% | 48% | 26% | 22% | 70% | 1.249 | 0.002 | 66% | 52% | 39% | 24% | 22% | 65% | 1.494 | <0.001 | 25% | 53% | 73% | 84% | 89% | 70% | 1.117 | | 0.137 |
| (5 items) | | Study 2 (Replication) | 78.1% | 58.4% | 36.3% | 16.0% | 8.0% | 74% | 1.079 | 0.252 | 56.7% | 41.4% | 27.6% | 13.9% | 8.9% | 69% | 0.013 | <0.001 | 66.2% | 40.5% | 20.9% | 10.4% | 6.8% | 73% | 1.050 | | 0.477 |
|  | |  |  |  |  |  |  |  |  |  |  |  |  |  |  |  |  |  |  |  |  |  |  |  |  | |  |
| Realistic Thinking | | Study 1 (Testing) | 88% | 77% | 55% | 33% | 11% | 74% | 0.521 | <0.001 | 82% | 66% | 42% | 23% | 9% | 73% | 0.392 | <0.001 | 28% | 46% | 66% | 79% | 89% | 74% | 1.223 | | <0.001 |
| (6 items) | | Study 2 (Replication) | 95.3% | 76.8% | 47.6% | 16.2% | 4.8% | 80% | 0.415 | <0.001 | 87.5% | 63.7% | 33.7% | 10.6% | 3.7% | 79% | 0.327 | <0.001 | 73.4% | 53.0% | 30.3% | 11.3% | 4.2% | 75% | 0.016 | | <0.001 |
|  | |  |  |  |  |  |  |  |  |  |  |  |  |  |  |  |  |  |  |  |  |  |  |  |  | |  |
| Meaningful Activities | | Study 1 (Testing) | 88% | 69% | 46% | 28% | 12% | 74% | 0.507 | <0.001 | 75% | 53% | 38% | 25% | 11% | 69% | 0.607 | <0.001 | 25% | 50% | 74% | 86% | 93% | 74% | 1.818 | | <0.001 |
| (4 items) | | Study 2 (Replication) | 81.4% | 64.6% | 40.6% | 16.6% | 5.8% | 77% | 0.618 | <0.001 | 62.8% | 47.3% | 30.3% | 13.3% | 5.3% | 73% | 0.675 | <0.001 | 72.9% | 45.4% | 26.6% | 7.7% | 4.1% | 76% | 1.616 | | <0.001 |
|  | |  |  |  |  |  |  |  |  |  |  |  |  |  |  |  |  |  |  |  |  |  |  |  |  | |  |
| Social Connections | | Study 1 (Testing) | 80% | 66% | 53% | 36% | 26% | 67% | 1.008 | 0.890 | 63% | 53% | 42% | 31% | 26% | 62% | 1.114 | 0.057 | 26% | 48% | 69% | 81% | 90% | 67% | 1.423 | | <0.001 |
| (3 items) | | Study 2 (Replication) | 71.5% | 56.2% | 35.3% | 21.8% | 12.7% | 71% | 0.913 | 0.081 | 54.1% | 38.6% | 27.7% | 18.3% | 9.8% | 66% | 0.010 | 0.843 | 62.4% | 42.5% | 21.2% | 10.5% | 5.5% | 74% | 0.015 | | <0.001 |
|  | |  |  |  |  |  |  |  |  |  |  |  |  |  |  |  |  |  |  |  |  |  |  |  |  | |  |
| Healthy Routines | | Study 1 (Testing) | 87% | 69% | 49% | 32% | 22% | 72% | 0.593 | <0.001 | 68% | 54% | 39% | 30% | 20% | 66% | 0.756 | <0.001 | 35% | 54% | 69% | 77% | 83% | 72% | 1.216 | | <0.001 |
| (3 items) | | Study 2 (Replication) | 83.5% | 67.5% | 44.8% | 16.7% | 7.7% | 77% | 0.519 | <0.001 | 59.5% | 46.6% | 31.6% | 16.3% | 8.3% | 70% | 0.788 | <0.001 | 63.3% | 43.5% | 26.1% | 13.9% | 8.6% | 70% | 0.012 | | <0.001 |
|  | |  |  |  |  |  |  |  |  |  |  |  |  |  |  |  |  |  |  |  |  |  |  |  |  | |  |
| Composite factor | | Study 1 (Testing) | 91% | 77% | 49% | 20% | 9% | 78% | 0.283 | <0.001 | 81% | 61% | 38% | 19% | 7% | 74% | 0.384 | <0.001 | 18% | 44% | 72% | 87% | 97% | 75% | 3.147711 | | <0.001 |
| (7 items) | | Study 2 (Replication) | 91.8% | 73.4% | 39.1% | 11.6% | 2.6% | 81% | 0.22 | <0.001 | 76.5% | 53.7% | 28.5% | 9.9% | 3.4% | 76% | 0.317 | <0.001 | 76.5% | 53.5% | 23.4% | 6.5% | 3.0% | 79% | 4.204 | | <0.001 |
| Table cells with bolded outline denote the weekly TYD frequency threshold associated with incremental improved outcome scores (ie., the TYD frequency threshold beyond which there is no statistically significant improvement in scores); *R* denotes a correlation coefficient and % variance explained (*R^2^*). OR – odds ratio; AUC – area under the curve. | | | | | | | | | | | | | | | | | | | | | | | | |  |  |  |
