# Supplementary material for: Measures of Daily Activities Associated With Mental Health (Things You Do Questionnaire): Development of a Preliminary Psychometric Study and Replication Study
Source: JMIR Form Res. 2022 Jul 5;6(7):e38837. doi: 10.2196/38837 (PMC9297144; doi:10.2196/38837)
Supplement: Multimedia Appendix 11 [file formative_v6i7e38837_app11.docx]

| **Multimedia Appendix 11**  **Table 1.** Invariance test solutions examining dimensionality in Study 2 sample. | | | | | | | | | | | |
| --- | --- | --- | --- | --- | --- | --- | --- | --- | --- | --- | --- |
| Subgroup considered | Test statistic |  | Configural model |  | Metric model | | Scalar model | | Strict model |  | Conclusion drawn |
| Reliability CFA -  Study 1 (Testing)  Study 2 (Replication)  Split | Observed-expected Fit; (χ2:df) < 3:1 |  | 3.501 |  | 3.582 |  | -- |  | -- |  | Sample CFA met factor formation & item loading only |
|  | Comparative fit index (CFI) > .90 |  | 0.995 |  | 0.995 |  | -- |  | -- |  |  |
|  | Tucker‐Lewis index (TLI) > .90 |  | 0.995 |  | 0.994 |  | -- |  | -- |  |  |
|  | RMSEA 90% CI value less than .08 |  | 0.027 |  | 0.027 |  | -- |  | -- |  |  |
|  | Comparative Model fit ꭓ^2^∆/df test (p-value) | | | 0.052 |  | <0.001 |  | -- |  |  |  |
|  |  |  |  |  |  |  |  |  |  |  |  |
| Age groups - | (χ2:df) < 3:1 |  | 1.149 |  | 1.382 |  | 1.537 |  | -- |  | Similarities in factor formation, item loading & mean scores across groups |
| <30; 30-44; | Comparative fit index (CFI) > .90 |  | 0.999 |  | 0.997 |  | 0.996 |  | -- |  |  |
| 45-65; >65 | Tucker‐Lewis index (TLI) > .90 |  | 0.999 |  | 0.997 |  | 0.996 |  | -- |  |  |
|  | RMSEA 90% CI value less than .08 |  | 0.008 |  | 0.019 |  | 0.023 |  | -- |  |  |
|  | Comparative Model fit ꭓ^2^∆/df test (p-value) | | | 0.146 |  | 0.031 |  | <0.001 | |  |  |
|  |  |  |  |  |  |  |  |  |  |  |  |
| PHQ-9 Severity groups; | (χ2:df) < 3:1 |  | 1.708 |  | 1.966 |  | -- |  | -- |  | Subgroup similarities in factor formation & item loading only |
| Min-Mild (<10); | Comparative fit index (CFI) > .90 |  | 0.994 |  | 0.992 |  | -- |  | -- |  |  |
| Moderate (10-14); | Tucker‐Lewis index (TLI) > .90 |  | 0.993 |  | 0.991 |  | -- |  | -- |  |  |
| Severe (15+) | RMSEA 90% CI value less than .08 |  | 0.023 |  | 0.028 |  | -- |  | -- |  |  |
|  | Comparative Model fit ꭓ^2^∆/df test (p-value) | | | 0.031 |  | <0.001 |  | -- |  |  |  |
|  |  |  |  |  |  |  |  |  |  |  |  |
| GAD-7 Severity groups | (χ2:df) < 3:1 |  | 1.532 |  | 1.77 |  | -- |  | -- |  | Subgroup similarities in factor formation & item loading only |
| Min-Mild (<10); | Comparative fit index (CFI) > .90 |  | 0.997 |  | 0.995 |  | -- |  | -- |  |  |
| Moderate (10-14); | Tucker‐Lewis index (TLI) > .90 |  | 0.996 |  | 0.994 |  | -- |  | -- |  |  |
| Severe (15+) | RMSEA 90% CI value less than .08 |  | 0.019 |  | 0.024 |  | -- |  | -- |  |  |
|  | Comparative Model fit ꭓ^2^∆/df test (p-value) | | | 0.1086 |  | <0.001 |  | -- |  |  |  |
|  |  |  |  |  |  |  |  |  |  |  |  |
| Education reported - | (χ2:df) < 3:1 |  | 1.89 |  | 2.03 |  | 1.999 |  | 1.955 |  | Similarities in subgroup met Strict invariance criteria |
| Tertiary; Other | Comparative fit index (CFI) > .90 |  | 0.997 |  | 0.997 |  | 0.997 |  | 0.997 |  |  |
|  | Tucker‐Lewis index (TLI) > .90 |  | 0.997 |  | 0.996 |  | 0.996 |  | 0.997 |  |  |
|  | RMSEA 90% CI value less than .08 |  | 0.021 |  | 0.023 |  | 0.023 |  | 0.022 |  |  |
|  | Comparative Model fit ꭓ^2^∆/df test (p-value) | | | 0.243 |  | 0.038 |  | 0.1732 |  |  |  |
|  |  |  |  |  |  |  |  |  |  |  |  |
| Employment reported - | (χ2:df) < 3:1 |  | 1.824 |  | 1.958 |  | -- |  | -- |  | Subgroup similarities in factor formation & item loading only |
| Employment; Other | Comparative fit index (CFI) > .90 |  | 0.998 |  | 0.997 |  | -- |  | -- |  |  |
|  | Tucker‐Lewis index (TLI) > .90 |  | 0.997 |  | 0.997 |  | -- |  | -- |  |  |
|  | RMSEA 90% CI value less than .08 |  | 0.02 |  | 0.022 |  | -- |  | -- |  |  |
|  | Comparative Model fit ꭓ^2^∆/df test (p-value) | | | 0.1204 |  | <0.001 | | -- |  |  |  |
|  |  |  |  |  |  |  |  |  |  |  |  |
| Gender; | (χ2:df) < 3:1 |  | 2.382 |  | 1.93 |  | -- |  | -- |  | Subgroup similarities in factor formation & item loading only |
| Male, female (Other) | Comparative fit index (CFI) > .90 |  | 0.997 |  | 0.997 |  | -- |  | -- |  |  |
|  | Tucker‐Lewis index (TLI) > .90 |  | 0.997 |  | 0.997 |  | -- |  | -- |  |  |
|  | RMSEA 90% CI value less than .08 |  | 0.021 |  | 0.022 |  | -- |  | -- |  |  |
|  | Comparative Model fit ꭓ^2^∆/df test (p-value) | | | 0.598 |  | <0.001 | | -- |  |  |  |

CFA – Confirmatory factor analysis. All models were based on weighted least square estimators
