# Supplementary material for: Measures of Daily Activities Associated With Mental Health (Things You Do Questionnaire): Development of a Preliminary Psychometric Study and Replication Study
Source: JMIR Form Res. 2022 Jul 5;6(7):e38837. doi: 10.2196/38837 (PMC9297144; doi:10.2196/38837)
Supplement: Multimedia Appendix 12 [file formative_v6i7e38837_app12.docx]

**Item Lists**

| **Study 1 - 96 Item List** | |  |
| --- | --- | --- |
| **Item Number** | **Primary/Secondary Clusters** | **Items** |
|  |  |  |
| TYD01 | Gratitude/Acceptance | I thought about things that I am grateful for |
| TYD02 | Healthy Routine/General | I kept a healthy daily routine |
| TYD03 | Healthy Routine/Substance | I had an alcohol free day |
| TYD04 | Plan/Organise | I took steps to organise what I did each day |
| TYD05 | Healthy Routine/Sleep | I went to bed and woke up at a regular time |
| TYD06 | Healthy Routine/Excesses | I avoided unhealthy habits (e.g., I chose not to have a drink, or gamble, etc) |
| TYD07 | Healthy Routine/Exercise | I planned or stuck to an exercise routine |
| TYD08 | Gratitude/Acceptance | I accepted a situation for what it is |
| TYD09 | Healthy Routine/Finances | I created and stuck to my budget |
| TYD10 | Activity/Meaning | I practiced a skill or did a hobby |
| TYD11 | Problem Solving | I took steps to solve a problem that was affecting me |
| TYD12 | Emotion Regulation/No excuses | I didn't make excuses |
| TYD13 | Activity/Meaning | I read, listened, or watched something I enjoyed |
| TYD14 | Plan/Future | I gave myself things to look forward to |
| TYD15 | Emotion Regulation/Pushing through | I pushed myself to do things that I didn’t feel like doing |
| TYD16 | Respect/Self | I treated myself with respect |
| TYD17 | Cognitive/Future | Instead of worrying about the past, I focused on my preferred future |
| TYD18 | Healthy Routine/Electronics | I kept my use of electronic devices or games to a healthy level |
| TYD19 | Healthy Routine/Substance | I avoided illicit drugs, and did not misuse medications |
| TYD20 | Emotion Regulation/Avoid chaos | I avoided chaos in my life |
| TYD21 | Healthy Routine/Sleep | I kept a relaxing bedtime routine, that did not involve watching videos or checking social media |
| TYD22 | Emotion Regulation/Coping | I dealt with feelings of frustration or impatience in a healthy way |
| TYD23 | Activity/Meaning | I put effort and time into something I wanted to change |
| TYD24 | Healthy Routine/Organised | I kept my home, living space, or workspace clean and organised |
| TYD25 | Emotion Regulation/Coping | I dealt with things that were creating stress |
| TYD26 | Healthy Routine/Hydration | I made sure I drank a healthy amount of water |
| TYD27 | Cognitive/Challenging | I identified unhelpful thoughts and tried to replace them with more helpful ones |
| TYD28 | Emotion Regulation/Pushing through | I faced a situation that was unpleasant but necessary |
| TYD29 | Social/Positive People | I socialised with positive people |
| TYD30 | Healthy Routine/Outside | I spent time outside |
| TYD31 | Social/Talking | I talked about my day with a friend or a family member |
| TYD32 | Cognitive/Perspective | I tried to keep things in perspective |
| TYD33 | Social/Talking | I had a meaningful conversation with someone |
| TYD34 | Activity/Interesting | I did a hobby or something that was of interest to me |
| TYD35 | Activity/Meaning | I spent time doing something I believed in |
| TYD36 | Healthy Routine/Social | I kept my use of social media and entertainment to a healthy level |
| TYD37 | Social/Help others | I did something to help others |
| TYD38 | Cognitive/Problem solving | Instead of thinking about my worries, I focused on doing something about them |
| TYD39 | Activity/Meaning | I spent time doing something I think is important |
| TYD40 | Plan/Realistic goals | I set realistic and achievable goals |
| TYD41 | Emotion Regulation/Patience | I practiced being patient |
| TYD42 | Problem Solving | I broke a large problem into smaller, more manageable steps |
| TYD43 | Emotion Regulation/Pushing through | I made myself do something because I knew it would be beneficial |
| TYD44 | Activity/Laugh, fun | I had a good laugh or did something that was fun |
| TYD45 | Plan/Personal responsibility | I took responsibility for the direction of my life |
| TYD46 | Healthy Routine/Electronics | I spent some time without the phone, TV, or internet on |
| TYD47 | Plan/Execute | I made a plan and stuck to it |
| TYD48 | Healthy Routine/Mental wellbeing | I did things which are good for my mental wellbeing |
| TYD49 | Healthy Routine/Relax | I did something to help me relax (e.g., slow breathing, stretching etc) |
| TYD50 | Healthy Routine/Exercise | I did 30 minutes of exercise |
| TYD51 | Social/Praise others | I encouraged or praised someone |
| TYD52 | Emotion Regulation/Realistic promises | I didn't promise or commit to doing things I couldn't do |
| TYD53 | Respect/Others | I treated others with respect |
| TYD54 | Plan/Future | I had something to look forward to |
| TYD55 | Cognitive/Challenging | I talked myself out of negative thinking |
| TYD56 | Social/Kindness others | I did something kind for someone else |
| TYD57 | Emotion Regulation/Coping | I fulfilled my responsibilities even though I didn't want to |
| TYD58 | Social/Positive People | I arranged to see friends |
| TYD59 | Activity/Meaning | I worked on an activity that was meaningful to me |
| TYD60 | Emotion Regulation/Pushing through | I pushed myself to do things that were difficult or triggered some stress |
| TYD61 | Cognitive/Perspective | I allowed myself to be less than perfect |
| TYD62 | Social/Positive People | I aimed to spend time with positive people |
| TYD63 | Social/Talking | I talked with a friend or family member on the phone |
| TYD64 | Healthy Routine/Nutrition | I prepared and ate a healthy meal |
| TYD65 | Gratitude/Acceptance | I tried to accept things that I couldn’t control or change |
| TYD66 | Activity/Satisfying | I did something that was very satisfying to me |
| TYD67 | Healthy Routine/Hygiene | I had a bath or shower |
| TYD68 | Cognitive/Challenging | I stopped myself from thinking unhelpful or unrealistic thoughts |
| TYD69 | Respect/Self | I praised myself when I did something well |
| TYD70 | Cognitive/Perspective | I kept a realistic perspective on things |
| TYD71 | Activity/Learn new | I tried to learn something new |
| TYD72 | Activity/Achieve goal | I did something to help me achieve my goals |
| TYD73 | Healthy Routine/Chores | I did work or chores around where I live (e.g., house, apartment, etc) |
| TYD74 | Healthy Routine/Exercise | I did some form of exercise (e.g. swimming, went for a walk, etc) |
| TYD75 | Social/Social media | I sent a personal email, text message, or made a post on social media to someone |
| TYD76 | Values/Spiritual | I acted in a way that is consistent with my personal values |
| TYD77 | Values/Spiritual | I acted with integrity and dignity |
| TYD78 | Activity/Improve quality of life | I did something to improve or maintain the quality of my life |
| TYD79 | Social/Help others | I did something to improve the quality of other people's lives |
| TYD80 | Environment | I did something to improve the quality of the physical environment |
| TYD81 | Healthy Routine/General satisfaction | I did something to improve my satisfaction with my life |
| TYD82 | Values/Spiritual | I did something to help me live my "ideal" life |
| TYD83 | Healthy Routine/Finances | I did something to improve or maintain my financial health |
| TYD84 | Healthy Routine/Nutrition | I avoided foods that caused emotional or physical problems, such as anxiety or indigestion |
| TYD85 | Healthy Routine/Sunlight | I got regular exposure to sunlight (e.g., 15-30 mins) |
| TYD86 | Values/Spiritual | I did something to improve or maintain my spiritual wellbeing |
| TYD87 | Social/Improve relationships | I did something to improve my relationships with people who are important to me |
| TYD88 | Activity/Enjoyable | I did something enjoyable |
| TYD89 | Activity/Avoid stagnant | I avoided being 'stagnant' |
| TYD90 | Respect/Reflection | I took time to reflect on myself and how I felt |
| TYD91 | Healthy Routine/Physical health | I did something to improve or maintain my physical health |
| TYD92 | Emotion Regulation/Expression | I expressed my feelings honestly, instead of suppressing them |
| TYD93 | Social/Improve belonging | I did something to improve my sense of belonging |
| TYD94 | Gratitude/Acceptance | I accepted my symptoms by allowing them to peak and pass |
| TYD95 | Emotion Regulation/Expression | I was able to say no when I did not want to do something |
| TYD96 | Healthy Routine/Silence, solitude | I spent time in Silence/ solitude |
|  |  |  |

| **Study 2 - 59 Item List** | |  |  |
| --- | --- | --- | --- |
| **Item Number** | **Primary/Secondary Clusters** | **Item** | |
|  |  |  | |
| TYD01 | Gratitude/Acceptance | I thought about things that I am grateful for | |
| TYD02 | Healthy Routine/General | I kept a healthy daily routine | |
| TYD03 | Healthy Routine/Substance | I had an alcohol free day | |
| TYD04 | Plan/Organise | I took steps to organise what I did each day | |
| TYD05 | Healthy Routine/Sleep | I went to bed and woke up at a regular time | |
| TYD06 | Healthy Routine/Excesses | I avoided unhealthy habits (e.g., I chose not to have a drink, or gamble, etc) | |
| TYD08 | Gratitude/Acceptance | I accepted a situation for what it is | |
| TYD16 | Respect/Self | I treated myself with respect | |
| TYD17 | Cognitive/Future | Instead of worrying about the past, I focused on my preferred future | |
| TYD18 | Healthy Routine/Electronics | I kept my use of electronic devices or games to a healthy level | |
| TYD21 | Healthy Routine/Sleep | I kept a relaxing bedtime routine, that did not involve watching videos or checking social media | |
| TYD22 | Emotion Regulation/Coping | I dealt with feelings of frustration or impatience in a healthy way | |
| TYD23 | Activity/Meaning | I put effort and time into something I wanted to change | |
| TYD24 | Healthy Routine/Organised | I kept my home, living space, or workspace clean and organised | |
| TYD25 | Emotion Regulation/Coping | I dealt with things that were creating stress | |
| TYD28 | Emotion Regulation/Pushing through | I faced a situation that was unpleasant but necessary | |
| TYD29 | Social/Positive People | I socialised with positive people | |
| TYD30 | Healthy Routine/Outside | I spent time outside | |
| TYD31 | Social/Talking | I talked about my day with a friend or a family member | |
| TYD33 | Social/Talking | I had a meaningful conversation with someone | |
| TYD34 | Activity/Interesting | I did a hobby or something that was of interest to me | |
| TYD35 | Activity/Meaning | I spent time doing something I believed in | |
| TYD37 | Social/Help others | I did something to help others | |
| TYD40 | Plan/Realistic goals | I set realistic and achievable goals | |
| TYD43 | Emotion Regulation/Pushing through | I made myself do something because I knew it would be beneficial | |
| TYD44 | Activity/Laugh, fun | I had a good laugh or did something that was fun | |
| TYD45 | Plan/Personal responsibility | I took responsibility for the direction of my life | |
| TYD47 | Plan/Execute | I made a plan and stuck to it | |
| TYD48 | Healthy Routine/Mental wellbeing | I did things which are good for my mental wellbeing | |
| TYD49 | Healthy Routine/Relax | I did something to help me relax (e.g., slow breathing, stretching etc) | |
| TYD51 | Social/Praise others | I encouraged or praised someone | |
| TYD54 | Plan/Future | I had something to look forward to | |
| TYD56 | Social/Kindness others | I did something kind for someone else | |
| TYD60 | Emotion Regulation/Pushing through | I pushed myself to do things that were difficult or triggered some stress | |
| TYD61 | Cognitive/Perspective | I allowed myself to be less than perfect | |
| TYD64 | Healthy Routine/Nutrition | I prepared and ate a healthy meal | |
| TYD66 | Activity/Satisfying | I did something that was very satisfying to me | |
| TYD67 | Healthy Routine/Hygiene | I had a bath or shower | |
| TYD68 | Cognitive/Challenging | I stopped myself from thinking unhelpful or unrealistic thoughts | |
| TYD69 | Respect/Self | I praised myself when I did something well | |
| TYD70 | Cognitive/Perspective | I kept a realistic perspective on things | |
| TYD71 | Activity/Learn new | I tried to learn something new | |
| TYD72 | Activity/Achieve goal | I did something to help me achieve my goals | |
| TYD73 | Healthy Routine/Chores | I did work or chores around where I live (e.g., house, apartment, etc) | |
| TYD74 | Healthy Routine/Exercise | I did some form of exercise (e.g. swimming, went for a walk, etc) | |
| TYD76 | Values/Spiritual | I acted in a way that is consistent with my personal values | |
| TYD77 | Values/Spiritual | I acted with integrity and dignity | |
| TYD78 | Activity/Improve quality of life | I did something to improve or maintain the quality of my life | |
| TYD80 | Environment | I did something to improve the quality of the physical environment | |
| TYD82 | Values/Spiritual | I did something to help me live my "ideal" life | |
| TYD85 | Healthy Routine/Sunlight | I got regular exposure to sunlight (e.g., 15-30 mins) | |
| TYD86 | Values/Spiritual | I did something to improve or maintain my spiritual wellbeing | |
| TYD88 | Activity/Enjoyable | I did something enjoyable | |
| TYD89 | Activity/Avoid stagnant | I avoided being 'stagnant' | |
| TYD90 | Respect/Reflection | I took time to reflect on myself and how I felt | |
| TYD91 | Healthy Routine/Physical health | I did something to improve or maintain my physical health | |
| TYD92 | Emotion Regulation/Expression | I expressed my feelings honestly, instead of suppressing them | |
| TYD94 | Gratitude/Acceptance | I accepted my symptoms by allowing them to peak and pass | |
| TYD96 | Healthy Routine/Silence, solitude | I spent time in Silence/ solitude | |

| **Study 2 - 21 Item, 5 Factor Solution** | |  | |
| --- | --- | --- | --- |
| **Item Number** | **Primary/Secondary Clusters** | | **Item** |
|  |  |  | |
| TYD02 | Healthy Routines | I kept a healthy daily routine | |
| TYD05 | Healthy Routines | I went to bed and woke up at a regular time | |
| TYD16 | Realistic Thinking | I treated myself with respect | |
| TYD17 | Realistic Thinking | Instead of worrying about the past, I focused on my preferred future | |
| TYD22 | Realistic Thinking | I dealt with feelings of frustration or impatience in a healthy way | |
| TYD29 | Social Connections | I socialised with positive people | |
| TYD31 | Social Connections | I talked about my day with a friend or a family member | |
| TYD33 | Social Connections | I had a meaningful conversation with someone | |
| TYD35 | Goals and Plans | I spent time doing something I believed in | |
| TYD44 | Meaningful Activity | I had a good laugh or did something that was fun | |
| TYD47 | Goals and Plans | I made a plan and stuck to it | |
| TYD54 | Goals and Plans | I had something to look forward to | |
| TYD61 | Realistic Thinking | I allowed myself to be less than perfect | |
| TYD64 | Healthy Routines | I prepared and ate a healthy meal | |
| TYD66 | Meaningful Activity | I did something that was very satisfying to me | |
| TYD68 | Realistic Thinking | I stopped myself from thinking unhelpful or unrealistic thoughts | |
| TYD70 | Realistic Thinking | I kept a realistic perspective on things | |
| TYD72 | Goals and Plans | I did something to help me achieve my goals | |
| TYD78 | Goals and Plans | I did something to improve or maintain the quality of my life | |
| TYD82 | Goals and Plans | I did something to help me live my "ideal" life | |
| TYD88 | Meaningful Activity | I did something enjoyable | |

**Things You Do Questionnaire: 21 Item Version (From Study 1 and Study 2)**

| **The Things You Do Questionnaire (TYDQ: 21-Item)**  Please indicate how often you have performed each of the following actions in the last week (7 days) | | | | | | | |
| --- | --- | --- | --- | --- | --- | --- | --- |
|  |  |  | Frequency per Week | | | | |
|  |  |  |  | | | | |
| **Item Number** | **Action** | | **0**  **(Not at all; 0 days per week)** | **1**  **(1-2 days per week)** | **2**  **(3-4 days per week)** | **3**  **(5-6 days per week)** | **4**  **(Everyday; 7 days per week)** |
| 1 | I had a good laugh or did something that was fun | |  |  |  |  |  |
| 2 | I had something to look forward to | |  |  |  |  |  |
| 3 | I did something that was very satisfying to me | |  |  |  |  |  |
| 4 | I did something enjoyable | |  |  |  |  |  |
| 5 | I treated myself with respect | |  |  |  |  |  |
| 6 | Instead of worrying about the past, I focused on my preferred future | |  |  |  |  |  |
| 7 | I dealt with feelings of frustration or impatience in a healthy way | |  |  |  |  |  |
| 8 | I allowed myself to be less than perfect | |  |  |  |  |  |
| 9 | I stopped myself from thinking unhelpful or unrealistic thoughts | |  |  |  |  |  |
| 10 | I kept a realistic perspective on things | |  |  |  |  |  |
| 11 | I spent time doing something I believed in | |  |  |  |  |  |
| 12 | I made a plan and stuck to it | |  |  |  |  |  |
| 13 | I did something to help me achieve my goals | |  |  |  |  |  |
| 14 | I did something to improve or maintain the quality of my life | |  |  |  |  |  |
| 15 | I did something to help me live my "ideal" life | |  |  |  |  |  |
| 16 | I kept a healthy daily routine | |  |  |  |  |  |
| 17 | I went to bed and woke up at a regular time | |  |  |  |  |  |
| 18 | I prepared and ate a healthy meal | |  |  |  |  |  |
| 19 | I socialised with positive people | |  |  |  |  |  |
| 20 | I had a meaningful conversation with someone | |  |  |  |  |  |
| 21 | I talked about my day with a friend or a family member | |  |  |  |  |  |
